# Design, Synthesis, and Bioevaluation of Matrine Derivatives as Potential Anti–Hepatitis B Virus Agents

**DOI:** 10.3390/biom15030436

**Published:** 2025-03-18

**Authors:** Ting-Ting Liu, Meng-Fan Xie, Xin Liu, Rong-Tao Li, Yao Bai, Zhi-Jun Zhang

**Affiliations:** Faculty of Life Science and Technology, Kunming University of Science and Technology, Kunming 650500, China; liutt@kust.edu.cn (T.-T.L.); xiemf@kust.edu.cn (M.-F.X.); liux@kust.edu.cn (X.L.); lirt@kust.edu.cn (R.-T.L.)

**Keywords:** matrine, matrine derivatives, diversity-oriented synthesis, hepatitis B virus, anti-HBV agents

## Abstract

Hepatitis B virus (HBV) is a causative reagent that frequently causes progressive liver diseases, leading to the development of acute hepatitis, chronic hepatitis, cirrhosis, and eventually hepatocellular carcinoma. Despite several antiviral drugs, including interferon-*α* and nucleotide derivatives, being approved for clinical treatment of HBV, critical issues remain unresolved, e.g., their low-to-moderate efficacy and adverse side effects, as well as resistant strains. In this study, twenty-three matrine derivatives were synthesized, and their antiviral effects against HBV were evaluated. Of these, eleven compounds inhibited HBeAg secretion significantly more than the positive control, lamivudine (3TC). Among the compounds synthesized in this study, compounds **4a** and **4d** had the most potent inhibitory activity, with IC_50_ value of 41.78 and 33.68 μM, respectively. Compounds **1h**, **4a,** and **4d** were also subjected to molecular docking studies. These compounds inhibited viral gene expression and viral propagation in a cell culture model. Thus, we believe our compounds could serve as resource for antiviral drug development.

## 1. Introduction

**Figure 1:**
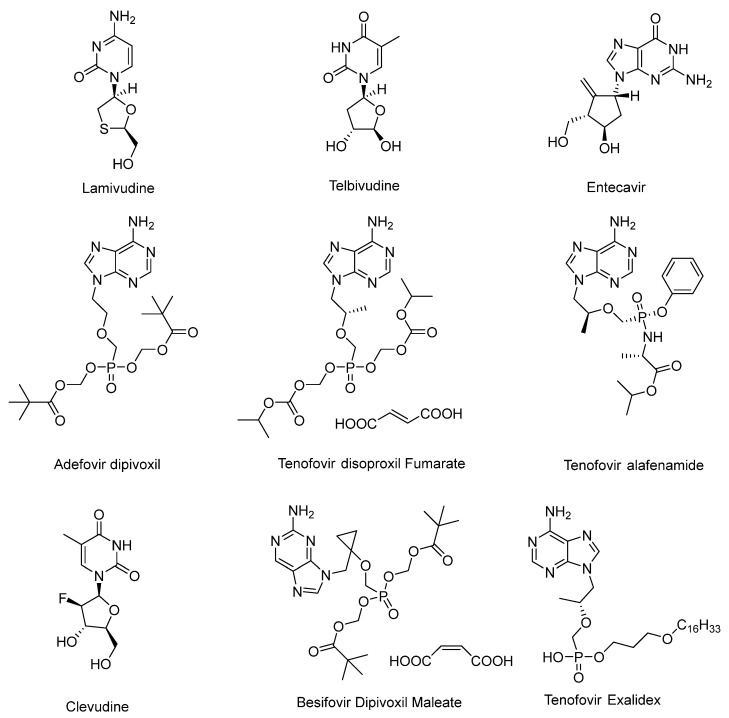
Nucleos(t)ide analogs that have been approved for marketing.

Chronic hepatitis B virus (HBV) infection is one of the greatest global healthcare threats and affects the lives of approximately 296 million people worldwide [1]. If left untreated, about one-third of patients die from end-stage liver diseases caused by chronic HBV infection, such as cirrhosis, hepatocellular carcinoma, and liver failure [2,3,4]. The current treatment options for hepatitis B include immune system modulation with two forms of interferon-*α* (IFN-*α*) and direct inhibition of the viral polymerase with five nucleos(t)ide analogs (Figure 1). These treatments can achieve suppression of HBV replication in the majority of patients [5,6]. However, hepatitis B surface antigen (HBsAg) loss or seroconversion and persistent low levels of intrahepatic covalently closed circular (ccc) DNA, which are considered the end points of “functional cure”, are rarely achieved, despite long-term antiviral treatment (HBsAg loss of less than 10% in 5 years) [7]. Therefore, it is crucial to develop potential treatment strategies and new agents that interrupt HBsAg production from (ccc) DNA, to achieve functional cure.

**Figure 2:**
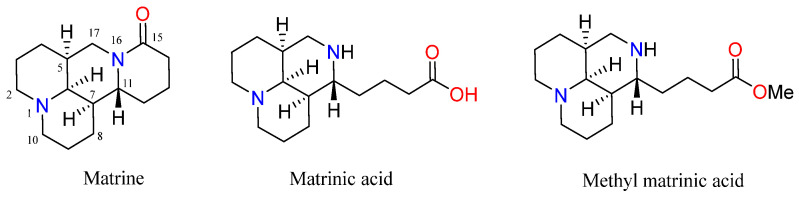
The chemical structures of matrine, matrinic acid, and methyl matrinic acid.

Matrine (Figure 2) is a naturally occurring small molecule compound that can be isolated from *Sophora favescens* (Kushen), *S. tonkinensis* (Shandougen), and *S. alopecuroides* (Kudouzi), which are widely distributed in Asia, Oceanica, and the Pacific islands [8,9]. Accumulating evidence has confirmed that matrine is an ideal multifunctional natural product for HBV patients [10]. Specifically, matrine exhibits good curative effect, few side effects, and low cost [11,12]. The chemical structure of matrine resembles purine, which directly leads to suppression of viral replication, immune regulation, and TNF reduction and, accordingly, ameliorates pathological changes in the liver [13]. Interferon (IFN) combined with matrine is the preferred treatment for HBV in China. Mechanistically, matrine enhance the ability of IFN to eliminate HBV [14]. However, as a natural product, matrine has some problems, such as relatively weak activity and severe toxicity. In addition, while matrine has good pharmacological activity, it has low lipid solubility, poor bioavailability, and a short half-life in vivo [15]. A pharmacokinetic study of matrine in rats showed that the bioavailability of matrine in rats was only 18.5% [16]. Therefore, further medicinal and chemical modifications are necessary. Many studies have shown that variants of matrine containing modifications to the D-ring, such as matrinic acid and methyl matrinic acid, can improve its deficiencies [17,18,19,20,21].

**Figure 3:**
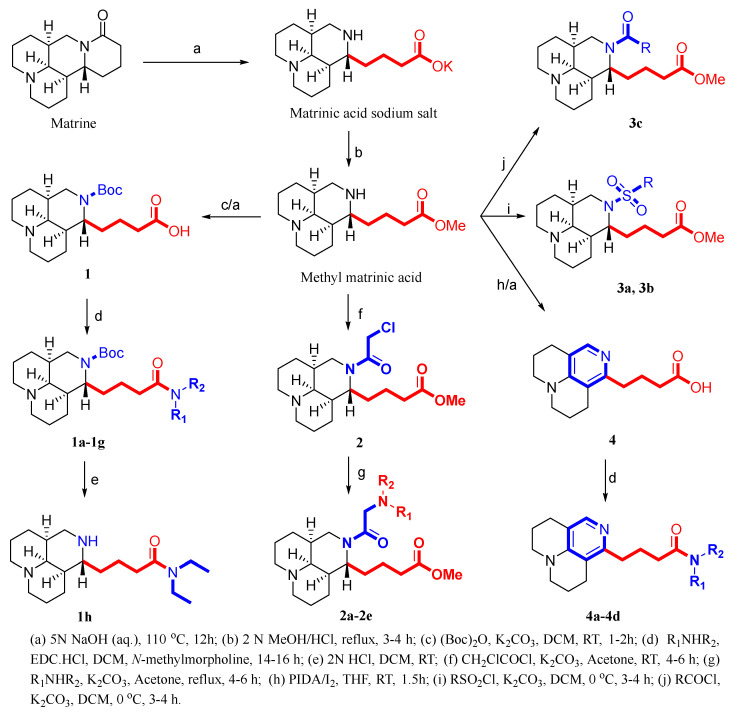
Design of the target matrinic acid derivatives.

Therefore, encouraged by the above results, and in continuation of our program aimed at the development of new anti-HBV agents, here we designed a series of novel methyl matrinic acid derivatives (Figure 3) by combining primary amines with matrinic acid at its C-15 position, by forming amide or sulfonamide bonds at the *N*-16 position of methyl matrinic acid, and by aromatizing the C-ring of methyl matrinic acid. The synthesized compounds were characterized with different spectroscopic techniques such as HRESIMS, ^1^H-NMR, and ^13^C-NMR. Moreover, these compounds were biologically tested for their antiviral effect against HBV (HBsAg, HBeAg, and HBV DNA). Additionally, a structure–activity relationship investigation was carried out to elucidate the relationship between the compounds’ activity and key structural features. Through this integrated approach, we gained valuable insights into the potential anti-HBV effects of the synthesized compounds, providing a path for future development.

## 2. Materials and Methods

### 2.1. Materials

All the chemicals used were of analytical grade. Iodobenzene diacetate (PIDA), iodine (I_2_), 1-(3-dimethylaminopropyl)-3-ethylcarbodiimide hydrochloride (EDC∙HCl), primary amines (R_1_NHR_2_), chloroacetyl chloride, di-*tert*-butyl decarbonate, acyl chlorides, sulfonyl chlorides, acetone, acetonitrile, and DCM were purchased from Sigma (St. Louis, MO, USA) and Sigma-Aldrich (Germany; Darmstadt), and were used as received. HBsAg and HBeAg secretion was measured using an ELISA diagnostic kit for HBsAg and HBeAg (Kehua Bio-Engineering Co., Ltd., Shanghai, China). Viral DNA was extracted for qPCR analysis using a TIANamp Virus DNA/RNA Kit (TianGen & Biotech Co., Ltd., Beijing, China).

### 2.2. Measurements

HRESIMS was performed using a LCMS-IT-TOF instrument (Applied Biosystems/MDS Sciex, Concord, ON, Canada). NMR spectra were obtained using a Bruker AV 600 MHz spectrometer with tetramethylsilane as an internal standard (Bruker BioSpin, Ettlingen, Germany) and methanol-*d*_4_ and chloroform-*d* as solvents, at 25 °C. Chemical shifts (*δ*) are expressed as parts per million (ppm).

### 2.3. Synthesis of the Compounds Under Investigation

#### 2.3.1. Synthesis of Key Intermediates of **1**, **2**, and **4**

As shown in Figure 3, matrine (10.0 g, 40 mmol) was added to a 5 N NaOH solution (60 mL), and the reaction mixture was refluxed for 12 h. After the reaction was complete, the solution was cooled and subsequently acidified with 10 N HCl to pH 3–5. The solvent was removed in vacuo, and the residue was dissolved with methanol (60 mL). The suspension was filtered, and the filtrate was concentrated. The residue was dissolved in 2 N HCl/MeOH (60 mL), and the mixture was refluxed for 3–4 h. The solvent was removed under reduced pressure to yield a crude product, which was purified by recrystallization from ethanol to achieve methyl matrinic acid (10.4 g, 92.8%).

Intermediate **1**. (Boc)_2_O (4.6 g, 21.3 mmol) and anhydrous K_2_CO_3_ (4.5 g, 32.1 mmol) were added to a solution of methyl matrinic acid (3.0 g, 10.7 mmol) in MeOH (40 mL) at room temperature. After being stirred at room temperature for 4 h, the reaction mixture was diluted with water and extracted three times with DCM. The organic layers were dried with anhydrous Na_2_SO_4_, and the solvent was removed under reduced pressure. The residue was added to a 5 N NaOH solution (50 mL). The mixture was refluxed for 12 h and cooled overnight to a precipitate, and many solids were precipitated. The solid was then transferred to concentrated HCl (10 N), and the pH of the solution was maintained at 3–5. The solvent was removed in vacuo and purified on a silica gel column to yield compound **1**, which was a white liquid (3.6 g) with 91.5% yield. ^1^H NMR (600 MHz, CD_3_OD): *δ*_H_ 3.72 (m, 1H), 3.38 (m, 2H), 3.12 (s, 1H), 2.98 (m, 2H), 2.71 (s, 1H), 2.37 (m, 2H), 2.15 (m, 2H), 1.96 (m, 1H), 1.80 (m, 1H), 1.71–1.64 (m, 4H), 1.61–1.48 (m, 8H), 1.39 (s, 9H). ^13^C NMR (150 MHz, CD_3_OD): *δ*_C_ 181.2, 157.4, 81.3, 64.6, 57.0, 57.0, 55.3, 44.2, 40.4, 37.3, 34.7, 33.4, 28.8, 28.8, 28.8, 28.3, 28.3, 23.9, 21.1, 20.9. The calculated HRMS (ESI) *m*/*z* for [C_20_H_34_N_2_O_4_ + H]^+^ was 367.2591, while the observed value was 367.2594. Purity: >99%.

Intermediate **2**. K_2_CO_3_ (2.8 g, 20 mmol) was added to a solution of methyl matrinic acid (3.0 g, 10.7 mmol) in acetone (50 mL). Chloroacetyl chloride (1.6 mL, 20 mmol) was added to this solution by dropping at 0 °C. The mixture was stirred for 3–4 h at room temperature. Then, water (200 mL) was added to the reaction mixture, followed by extraction with ethylactate (3 × 20 mL). The organic phase was dried using sodium sulfate, filtered, and removed under vacuum to obtain crude products. These crude products were further purified on a silica gel column to obtain compound **2**, which was a white liquid (2.6 g) with a 68.5% yield. ^1^H NMR (600 MHz, CDCl_3_): *δ*_H_ 4.95 (d, *J* = 14.1 Hz, 1H), 4.84 (d, *J* = 14.1 Hz, 1H), 4.21 (d, *J* = 6.2 Hz, 2H), 4.08 (s, 2H), 3.96 (t, *J* = 12.8 Hz, 1H), 3.64 (s, 3H), 3.60 (m, 2H), 3.46 (dd, *J* = 13.8, 5.4 Hz, 1H), 3.19 (s, 1H), 2.62 (m, 2H), 2.36 (m, 2H), 2.30 (m, 2H), 2.19 (m, 1H), 2.11 (m, 1H), 1.99 (m, 2H), 1.73 (m, 3H), 1.61 (m, 2H). ^13^C NMR (150 MHz, CDCl_3_): *δ*_C_ 174.1, 167.7, 64.2, 63.4, 56.3, 55.8, 51.7, 42.6, 40.8, 38.0, 37.8, 33.6, 26.8, 26.2, 25.9, 21.5, 18.9, 18.8. The calculated HRMS (ESI) *m*/*z* for [C_18_H_29_ClN_2_O_3_ + H]^+^ was 357.1939, while the observed value was 357.1942. Purity: >95%.

Intermediate **4**. PIDA (650 mg, 2.0 mmol) and I_2_ (506 mg, 2.0 mmol) were added to a stirred solution of methyl matrinic acid (300 mg, 1.0 mmol) in solvent [THF (100 mL), with no need for excluding H_2_O], and the reaction mixture was stirred at room temperature unless otherwise noted. After 90 min, the reaction was quenched with Na_2_S_2_O_3_ (aq) and stirred until the mixture was pale yellow (a few minutes). Finally, the reaction was extracted with DCM, and the combined organic layers were dried over anhydrous Na_2_SO_4_, filtered, and concentrated in vacuo. The residue was added to a 5 N NaOH solution (50 mL). The mixture was refluxed for 12 h and cooled overnight to a precipitate, and many solids were precipitated. The solids were then transferred to concentrated HCl (10 N), and the pH of the solution was maintained at 3–5. The solvent was removed in vacuo and purified on a silica gel column to give compound **4**, which was a white liquid (100 mg) with a 38.5% yield. ^1^H NMR (600 MHz, CD_3_OD): *δ*_H_ 7.57 (1H, s), 3.41 (m, 4H), 2.69 (m, 6H), 2.33 (m, 2H), 1.92 (m, 4H), 1.82 (m, 2H). ^13^C NMR (150 MHz, CD_3_OD): δ_C_ 176.6, 153.9, 148.8, 135.0, 117.0, 115.4, 51.3, 50.6, 34.0, 30.9, 25.0, 24.7, 23.1, 20.7, 20.5. The calculated HRMS (ESI) *m*/*z* for [C_15_H_21_N_2_O_2_ + H]^+^ was 261.1595, while the observed value was 261.1598. Purity: >99%.

#### 2.3.2. General Method for Synthesis of **1a**–**1h**

A mixture of compound **1** (0.3 mmol), different secondary amines (0.45 mmol), and EDC∙HCl (0.45 mmol) in DCM (5 mL) was added to a flame-dried flask at room temperature. After stirring evenly for 30–40 min, *N*-methylmorpholine (0.45 mmol) was added and allowed to react at room temperature for 14–16 h. Then, the mixture was diluted with DCM (10 mL). It was washed with saturated aq. Na_2_CO_3_ (20 mL × 2) and brine (20 mL) and dried over anhydrous Na_2_SO_4_. Finally, it was concentrated in vacuo and purified by silica gel column chromatography, eluting with DCM/MeOH (*v*/*v* = 9/1 or 4/1) to yield target compounds **1a**–**1g**.

Compound **1a** was synthesized from **1** (0.3 mmol) reacted with piperidine (0.45 mmol), as described in the general procedure. The obtained compound was a yellow liquid (39 mg) with a 30.0% yield. ^1^H NMR (600 MHz, CD_3_OD): *δ*_H_ 4.60 (s, 1H), 3.79 (m, 1H), 3.47 (m, 2H), 3.41 (m, 2H), 2.62 (s, 2H), 2.41 (m, 1H), 2.28 (m, 1H), 1.95 (m, 1H), 1.83–1.56 (m, 10H), 1.52–1.38 (m, 10H), 1.39 (s, 9H). ^13^C NMR (150 MHz, CD_3_OD): *δ*_C_ 173.8, 157.9, 80.7, 64.4, 57.7, 57.7, 55.2, 48.1, 48.1, 44.4, 43.9, 41.8, 35.7, 34.0, 29.4, 28.8, 28.8, 28.8, 27.8, 26.8, 25.5, 25.5, 23.7, 22.3, 22.0. The calculated HRMS (ESI) *m*/*z* for [C_25_H_43_N_3_O_3_ + H]^+^ was 434.3377, while the observed value was 434.3383. Purity: >93%.

Compound **1b** was synthesized from **1** (0.3 mmol) reacted with morpholine (0.45 mmol), as described in the general procedure. The obtained compound was a yellow liquid (69 mg) with a 52.6% yield. ^1^H NMR (600 MHz, CD_3_OD): *δ*_H_ 3.80 (m, 1H), 3.57 (m, 5H), 3.48 (m, 6H), 2.64 (m, 2H), 2.41 (m, 1H), 2.31 (m, 1H), 1.96 (s, 1H), 1.71 (m, 5H), 159 (m, 4H), 1.51 (m, 3H), 1.39 (s, 9H). ^13^C NMR (150 MHz, CD_3_OD): *δ*_C_ 174.2, 157.5, 80.6, 67.9, 67.8, 64.4, 57.9, 57.8, 55.2, 47.9, 44.4, 43.2, 41.7, 36.4, 34.0, 33.2, 30.5, 29.5, 28.9, 28.9, 28.9, 23.5, 22.3, 22.0. The calculated HRMS (ESI) *m*/*z* for [C_24_H_41_N_3_O_4_ + H]^+^ was 436.3170, while the observed value was 436.3177. Purity: >99%.

Compound **1c** was synthesized from **1** (0.3 mmol) reacted with thiomorpholine (0.45 mmol), as described in the general procedure. The obtained compound was a yellow liquid (69 mg) with a 50.3% yield. ^1^H NMR (600 MHz, CD_3_OD): *δ*_H_ 3.78 (m, 5H), 3.48 (m, 1H), 2.62 (m, 2H), 2.58 (m, 2H), 2.43 (m, 1H), 2.31 (m, 1H), 1.93 (s, 1H), 1.78 (m, 5H), 1.63 (m, 3H), 1.49 (m, 3H), 1.42 (m, 6H), 1.39 (s, 9H). ^13^C NMR (150 MHz, CD_3_OD): *δ*_C_ 174.0, 157.5, 80.6, 64.4, 57.9, 57.8, 55.2, 49.8, 45.7, 44.5, 41.8, 35.8, 33.9, 33.1, 30.1, 29.5, 28.9, 28.9, 28.9, 28.8, 28.2, 23.5, 22.3, 22.0. The calculated HRMS (ESI) *m*/*z* for [C_24_H_41_N_3_O_3_S + H]^+^ was 452.2941, while the observed value was 452.2943. Purity: >98%.

Compound **1d** was synthesized from **1** (0.3 mmol) reacted with pyrrolidine (0.45 mmol), as described in the general procedure. The obtained compound was a yellow liquid (15 mg) with a 12.5% yield. ^1^H NMR (600 MHz, CD_3_OD): *δ*_H_ 3.79 (m, 1H), 3.43 (m, 4H), 3.33 (m, 3H), 2.73 (m, 2H), 2.33 (m, 2H), 2.26 (m, 2H), 1.89 (m, 5H), 1.81 (m, 5H), 1.67 (m, 3H), 1.52 (m, 6H), 1.40 (s, 9H). ^13^C NMR (150 MHz, CD_3_OD): *δ*_C_ 174.5, 157.4, 81.6, 64.3, 57.6, 57.5, 55.2, 49.6, 48.0, 46.9, 43.5, 41.3, 35.1, 33.5, 29.3, 28.8, 28.8, 28.8, 28.7, 27.0, 25.4, 23.0, 22.0, 21.7. The calculated HRMS (ESI) *m*/*z* for [C_24_H_41_N_3_O_3_ + H]^+^ was 420.3221, while the observed value was 420.3224. Purity: >92%.

Compound **1e** was synthesized from **1** (0.3 mmol) reacted with *N*-Isopropylmethylamine (0.45 mmol), as described in the general procedure. The obtained compound was a yellow liquid (21 mg) with a 17.5% yield. ^1^H NMR (600 MHz, CD_3_OD): *δ*_H_ 4.70 (m, 1H), 3.80 (m, 1H), 3.50 (m, 1H), 2.81 (s, 3H), 2.62 (m, 2H), 2.33 (m, 2H), 1.93 (s, 1H), 1.79 (m, 6H), 1.61 (m, 2H), 1.45 (m, 3H), 1.52 (m, 6H), 1.38 (s, 9H), 1.21 (d, *J* = 6.8 Hz, 3H), 1.10 (d, *J* = 6.8 Hz, 3H). ^13^C NMR (150 MHz, CD_3_OD): *δ*_C_ 175.0, 157.5, 80.6, 64.4, 57.9, 57.8, 55.2, 45.5, 41.8, 35.8, 34.6, 33.3, 30.1, 29.5, 28.9, 28.9, 28.9, 26.4, 23.3, 22.3, 22.0, 20.6, 19.5. The calculated HRMS (ESI) *m*/*z* for [C_24_H_43_N_3_O_3_ + H]^+^ was 422.3377, while the observed value was 422.3381. Purity: >95%.

Compound **1f** was synthesized from **1** (0.3 mmol) reacted with *N*-propylmethylamine (0.45 mmol), as described in the general procedure. The obtained compound was a yellow liquid (63 mg) with a 49.2% yield. ^1^H NMR (600 MHz, CD_3_OD): *δ*_H_ 3.80 (m, 1H), 3.48 (m, 1H), 3.28 (m, 2H), 2.97 (s, 3H), 2.62 (m, 2H), 2.40 (m, 1H), 2.31 (m, 1H), 1.93 (m, 1H), 1.75 (m, 5H), 1.63 (m, 5H), 1.49 (m, 6H), 1.49 (m, 5H), 1.39 (s, 3H), 0.87 (t, *J* = 7.4 Hz, 3H). ^13^C NMR (150 MHz, CD_3_OD): *δ*_C_ 175.4, 157.5, 81.1, 64.4, 57.9, 57.8, 55.2, 52.7, 50.4, 44.2, 41.8, 36.1, 34.1, 33.5, 30.1, 29.5, 28.8, 28.8, 28.8, 23.7, 23.3, 22.6, 21.4, 11.5. The calculated HRMS (ESI) *m*/*z* for [C_24_H_43_N_3_O_3_ + H]^+^ was 422.3377, while the observed value was 422.3382. Purity: >96%.

Compound **1g** was synthesized from **1** (0.3 mmol) reacted with diethylamine (0.45 mmol), as described in the general procedure. The obtained compound was a yellow liquid (66 mg) with a 52.4% yield. ^1^H NMR (600 MHz, CD_3_OD): *δ*_H_ 3.80 (m, 1H), 3.48 (m, 1H), 335 (m, 2H), 3.31 (m, 2H), 2.63 (m, 2H), 2.40 (m, 1H), 2.28 (m, 1H), 1.93 (m, 1H), 1.78 (m, 6H), 1.72 (m, 2H), 1.48 (m, 2H), 1.42 (m, 4H), 1.39 (s, 3H), 1.35 (m, 3H), 1.13 (t, *J* = 7.2 Hz, 3H), 1.03 (t, *J* = 7.2 Hz, 3H). ^13^C NMR (150 MHz, CD_3_OD): *δ*_C_ 174.7, 157.5, 81.3, 64.4, 57.9, 57.8, 55.2, 44.3, 43.5, 42.2, 41.4, 36.2, 33.6, 33.3, 30.1, 29.5, 28.9, 28.9, 28.9, 23.8, 22.3, 22.0, 14.6, 13.3. The calculated HRMS (ESI) *m*/*z* for [C_24_H_43_N_3_O_3_ + H]^+^ was 422.3377, while the observed value was 422.3384. Purity: >93%.

Compound **1h**. 2N HCl gas was slowly added to a solution of compound **1g** (0.1 mmol) in DCM at room temperature until the end of the reaction. The reaction solution was neutralized to pH 8–9 by adding 10% Na_2_CO_3_ solution. The resulting mixture was filtered and concentrated under reduced pressure. The crude product was purified by silica column chromatography, eluting with DCM/MeOH (*v*/*v* = 4/1) to yield compound **1h**. The obtained compound was a yellow liquid (8 mg) with a 26.2% yield. ^1^H NMR (600 MHz, CD_3_OD): *δ*_H_ 3.48 (m, 1H), 3.35 (m, 4H), 2.94 (m, 1H), 2.80 (m, 2H), 2.40 (m, 2H), 2.19 (s, 1H), 2.01 (m, 3H), 1.89 (m, 2H), 1.77 (m, 2H), 1.70 (m, 2H), 1.61 (m, 4H), 1.51 (m, 2H), 1.43 (m, 3H),1.13 (t, *J* = 7.1 Hz, 3H), 1.05 (t, *J* = 7.1 Hz, 3H). ^13^C NMR (150 MHz, CD_3_OD): *δ*_C_ 173.9, 63.2, 58.0, 57.9, 54.0, 45.0, 43.4, 41.7, 39.6, 34.7, 33.4, 31.0, 27.8, 26.8, 21.6, 21.3, 20.4, 14.3, 13.2. The calculated HRMS (ESI) *m*/*z* for [C_19_H_35_N_3_O + H]^+^ was 322.2853, while the observed value was 322.2857. Purity: >97%.

#### 2.3.3. General Method for Synthesis of **2a**–**2e**

Different secondary amines (0.35 mmol) and **2** (0.3 mmol) were dissolved in acetone (10 mL), followed by the addition of K_2_CO_3_ (0.13 g, 1.0 mmol) to the solution. The mixture was refluxed for 4–6 h. The hot precipitate was filtered, and the filtrate was evaporated under reduced pressure to yield the crude product, which was purified by silica gel column chromatography, eluting with DCM/MeOH (*v*/*v* = 9/1 or 4/1) to yield oil compounds **2a**–**2e**.

Compound **2a** was synthesized from **2** (0.3 mmol) reacted with piperidine (0.35 mmol), as described in the general procedure. The obtained compound was a yellow liquid (67 mg) with a 55.5% yield. ^1^H NMR (600 MHz, CD_3_OD): 4.14 (s, 1H),3.64 (m, 1H), 3.57 (s, 3H), 3.38 (m, 1H), 3.17 (m, 2H), 2.64 (s, 1H), 2.60 (m, 2H), 2.46 (m, 5H), 2.31 (m, 3H), 1.96 (s, 1H), 1.88 (s, 2H), 1.76 (m, 6H), 1.63–1.33 (m, 11H). ^13^C NMR (150 MHz, CD_3_OD): δ_C_ 175.7, 173.8, 63.7, 62.9, 57.9, 57.4, 55.9, 55.9, 54.2, 52.0, 44.6, 41.2, 35.3, 34.4, 34.3, 30.6, 29.2, 26.5, 26.5, 24.9, 22.9, 22.4, 22.1. The calculated HRMS (ESI) *m*/*z* for [C_23_H_39_N_3_O_3_ + H]^+^ was 406.3064, while the observed value was 406.3065. Purity: >97%.

Compound **2b** was synthesized from **2** (0.3 mmol) reacted with diethylamine (0.35 mmol), as described in the general procedure. The obtained compound was a white liquid (54 mg) with a 45.5% yield. ^1^H NMR (600 MHz, CD_3_OD): *δ*_H_ 4.20 (m, 1H), 3.87 (m, 1H), 3.58 (s, 3H), 3.38 (m, 2H), 3.05 (s, 4H), 2.66 (m, 2H), 2.32 (m, 2H), 1.78 (m, 2H), 1.61 (m, 4H), 1.66–1.38 (m, 11H), 1.20 (t, *J* = 6.9 Hz, 6H). ^13^C NMR (150 MHz, CD_3_OD): *δ*_C_ 175.7, 165.6, 63.3, 57.7, 57.2, 54.2, 52.0, 50.8, 50.8, 43.3, 41.9, 40.9, 34.6, 34.3, 34.2, 30.7, 28.7, 22.7, 22.2, 21.8, 10.2, 10.2. The calculated HRMS (ESI) *m*/*z* for [C_22_H_39_N_3_O_3_ + H]^+^ was 394.3064, while the observed value was 394.3068. Purity: >97%.

Compound **2c** was synthesized from **2** (0.3 mmol) reacted with pyrrolidine (0.35 mmol), as described in the general procedure. The obtained compound was a yellow liquid (100 mg) with a 85.5% yield. ^1^H NMR (600 MHz, CD_3_OD): *δ*_H_ 4.15 (m, 1H), 3.84 (m, 2H), 3.58 (s, 3H), 3.36 (m, 2H), 3.00 (s, 4H), 2.67 (m, 2H), 2.32 (m, 2H), 1.89 (m, 6H), 1.78 (m, 3H), 1.66–1.38 (m, 11H). ^13^C NMR (150 MHz, CD_3_OD): *δ*_C_ 177.4, 168.5, 63.5, 58.7, 57.8, 57.3, 55.7, 55.7, 54.1, 52.0, 43.8, 40.9, 34.7, 34.4, 34.3, 30.6, 28.8, 24.3, 24.3, 22.8, 22.2, 21.9. The calculated HRMS (ESI) *m*/*z* for [C_22_H_37_N_3_O_3_ + H]^+^ was 392.2908, while the observed value was 392.2912. Purity: >97%.

Compound **2d** was synthesized from **2** (0.3 mmol) reacted with *N*-propylmethylamine (0.35 mmol), as described in the general procedure. The obtained compound was a yellow liquid (24 mg) with a 20.4% yield. ^1^H NMR (600 MHz, CD_3_OD): *δ*_H_ 4.13 (m, 1H), 3.58 (s, 3H), 3.41 (m, 2H), 3.28 (m, 1H), 3.20 (m, 1H), 2.61 (m, 2H), 2.33 (m, 4H), 2.22 (m, 2H), 1.95 (m, 2H),1.77 (m, 4H), 1.89 (m, 6H), 1.78 (m, 3H), 1.66–1.38 (m, 14H), 0.84 (t, *J* = 7.4 Hz, 3H). ^13^C NMR (150 MHz, CD_3_OD): *δ*_C_ 175.7, 171.7, 63.7, 61.8, 61.0, 57.8, 57.4, 54.1, 52.0, 44.6, 42.9, 41.3, 35.3, 34.4, 34.2, 30.6, 29.2, 23.0, 22.4, 22.1, 21.2, 12.2. The calculated HRMS (ESI) *m*/*z* for [C_22_H_39_N_3_O_3_ + H]^+^ was 394.3064, while the observed value was 394.3074. Purity: >92%.

Compound **2e** was synthesized from **2** (0.3 mmol) reacted with morpholine (0.35 mmol), as described in the general procedure. The obtained compound was a yellow liquid (19 mg) with a 15.7% yield. ^1^H NMR (600 MHz, CD_3_OD): *δ*_H_^1^H NMR (600 MHz, CD_3_OD): *δ*_H_ 3.63 (m, 3H), 3.58 (m, 3H), 3.19 (m, 4H), 2.60 (m, 2H), 2.45 (m, 4H), 2.28 (m, 2H), 1.93 (m, 3H), 1.76 (m, 3H), 1.65–1.33 (m, 11H). ^13^C NMR (150 MHz, CD_3_OD): *δ*_C_ 175.9, 171.0, 67.7, 63.7, 62.8, 58.3, 57.9, 57.4, 54.9, 54.7, 54.1, 52.0, 44.5, 41.2, 35.2, 34.4, 33.4, 30.7, 29.2, 23.0, 22.4, 22.1. The calculated HRMS (ESI) *m*/*z* for [C_22_H_37_N_3_O_4_ + H]^+^ was 408.2857, while the observed value was 408.2864. Purity: >90%.

#### 2.3.4. General Method for Synthesis of **3a**–**3c**

Different acyl/sulfonyl chlorides (0.35 mmol) and methyl matrinic acid (0.3 mmol) were dissolved in acetonitrile (10 mL), followed by the addition of K_2_CO_3_ (0.13 g, 1.0 mmol) to the solution. The mixture was reacted at 0 °C for 3–4 h. The precipitate was filtered, and the filtrate was evaporated under reduced pressure to yield a crude product, which was purified by silica gel column chromatography, eluting with DCM/MeOH (*v*/*v* = 9/1 or 4/1) to yield compounds **3a**–**3c**.

Compound **3a** was synthesized from methyl matrinic acid (0.3 mmol) reacted with *p*-toluenesulfonyl chloride (0.35 mmol), as described in the general procedure. The obtained compound was a yellow liquid (100 mg) with a 75.6% yield. ^1^H NMR (600 MHz, CDCl_3_): *δ*_H_ 7.71 (d, *J* = 7.9 Hz, 2H), 7.27 (d, *J* = 7.9 Hz, 2H), 3.64 (s, 3H), 3.55 (m, 1H), 3.51 (m, 1H), 3.23 (t, *J* = 12.0 Hz, 1H), 2.67 (d, *J* = 11.0 Hz, 1H), 2.62 (d, *J* = 11.0 Hz, 1H), 2.41 (s, 3H), 2.26 (m, 1H), 2.18 (m, 1H), 2.05 (s, 1H), 2.00 (m, 1H), 1.87 (m, 5H), 1.77 (m, 1H), 1.66 (m, 2H), 1.54 (m, 2H), 1.48 (m, 2H), 1.40 (m, 1H), 1.35 (m, 4H), 1.26 (m, 1H). ^13^C NMR (150 MHz, CDCl_3_): *δ*_C_ 174.2, 143.0, 137.6, 129.5, 129.5, 127.6, 127.6, 63.5, 57.9, 57.0, 57.0, 51.6, 48.5, 39.3, 35.1, 34.0, 29.8, 28.0, 27.8, 21.7, 21.1, 21.0, 20.8. The calculated HRMS (ESI) *m*/*z* for [C_23_H_34_N_2_O_4_S + H]^+^ was 435.2312, while the observed value was 435.2315. Purity: >97%.

Compound **3b** was synthesized from methyl matrinic acid (0.3 mmol) reacted with 4-*tert*-butylbenzenesulfonyl chloride (0.35 mmol), as described in the general procedure. The obtained compound was a yellow liquid (72 mg) with a 50.6% yield. ^1^H NMR (600 MHz, CDCl_3_): *δ*_H_ 7.54 (d, *J* = 8.2 Hz, 2H), 7.46 (d, *J* = 8.2 Hz, 2H), 3.63 (s, 3H), 3.56 (m, 2H), 3.25 (t, *J* = 12.0 Hz, 1H), 2.65 (d, *J* = 11.0 Hz, 1H), 2.59 (d, *J* = 11.0 Hz, 1H), 2.23 (m, 1H), 2.14 (m, 1H), 2.05 (s, 1H), 2.01 (m, 1H), 1.90 (m, 1H), 1.83 (m, 5H), 1.76 (m, 1H), 1.70 (m, 2H), 1.54 (m, 2H), 1.46 (m, 2H), 1.35 (m, 2H), 1.33 (s, 9H). ^13^C NMR (150 MHz, CDCl_3_): *δ*_C_ 174.1, 155.8, 137.5, 127.4, 127.4, 125.7, 125.7, 63.3, 57.7, 56.9, 56.8, 51.5, 48.0, 39.3, 35.1, 35.0, 33.9, 31.1, 31.1, 31.1, 29.9, 27.9, 27.8, 20.9, 20.9, 20.8. The calculated HRMS (ESI) *m*/*z* for [C_26_H_40_N_2_O_4_S + H]^+^ was 477.2782, while the observed value was 477.2786. Purity: >97%.

Compound **3c** was synthesized from methyl matrinic acid (0.3 mmol) reacted with 4-dimethylaminobenzoyl chloride (0.35 mmol), as described in the general procedure. The obtained compound was a yellow liquid (55 mg) with a 43.2% yield. ^1^H NMR (600 MHz, CDCl_3_): *δ*_H_ 7.42 (d, *J* = 8.3 Hz, 2H), 6.65 (d, *J* = 8.3 Hz, 2H), 3.93 (m, 1H), 3.68 (m, 1H), 3.64 (s, 3H), 3.49 (m, 1H), 2.99 (s, 6H), 2.79 (d, *J* = 11.0 Hz, 1H), 2.76 (d, *J* = 11.0 Hz, 1H), 2.23 (m, 2H), 2.21 (m, 1H), 2.07 (s, 1H), 2.05 (s, 1H), 1.98 (m, 1H), 1.87 (m, 5H), 1.62 (m, 3H), 1.54 (m, 3H), 0.86 (s, 4H). ^13^C NMR (150 MHz, CDCl_3_): *δ*_C_ 174.4, 173.4, 151.5, 129.6, 129.2, 124.5, 111.3, 111.3, 64.0, 60.5, 57.1, 56.9, 55.6, 51.5, 41.2, 40.4, 40.4, 39.1, 34.1, 31.5, 30.3, 27.7, 22.0, 21.3, 21.2. The calculated HRMS (ESI) *m*/*z* for [C_25_H_37_N_3_O_3_ + H]^+^ was 428.2908, while the observed value was 428.2911. Purity: >92%.

#### 2.3.5. General Method for Synthesis of **4a**–**4d**

A mixture of compound **4** (0.3 mmol), different secondary amines (0.4 mmol), and EDC∙HCl (0.4 mmol) in DCM (5 mL) was added to a flame-dried flask at room temperature. After stirring evenly for 30–40 min, *N*-methylmorpholine (0.4 mmol) was added and allowed to react at room temperature for 14–16 h. Then, the mixture was diluted with DCM (20 mL), washed with saturated aq. Na_2_CO_3_ (30 mL × 2) and brine (30 mL), and dried over anhydrous Na_2_SO_4_. Finally, it was concentrated in vacuo and purified by silica gel column chromatography, eluting with DCM/MeOH (*v*/*v* = 9/1 or 4/1) to yield target compounds **4a**–**4d**.

Compound **4a** was synthesized from **4** (0.3 mmol) reacted with diethylamine (0.4 mmol), as described in the general procedure. The obtained compound was a colorless liquid (12 mg) with a 12.5% yield. ^1^H NMR (600 MHz, CD_3_OD): *δ*_H_ 7.50 (s, 1H), 3.29 (m, 5H), 3.21 (m, 3H), 2.63 (m, 2H), 2.58 (m, 4H), 2.33 (m, 2H), 1.88 (m, 4H), 1.79 (m, 2H), 1.10 (d, *J* = 7.1 Hz, 1H), 1.03 (d, *J* = 7.1 Hz, 1H). ^13^C NMR (150 MHz, CDCl_3_): *δ*_C_ 174.3, 155.6, 150.3, 144.1, 115.6, 114.4, 50.7, 49.9, 43.5, 41.5, 34.3, 33.3, 26.0, 25.4, 24.1, 22.0, 22.0, 14.5, 13.5. The calculated HRMS (ESI) *m*/*z* for [C_19_H_29_N_3_O + H]^+^ was 316.2383, while the observed value was 316.2387. Purity: >95%.

Compound **4b** was synthesized from **4** (0.3 mmol) reacted with pyrrolidine (0.4 mmol), as described in the general procedure. The obtained compound was a colorless liquid (34 mg) with a 35.8% yield. ^1^H NMR (600 MHz, CD_3_OD): *δ*_H_ 7.49 (s, 1H), 3.38 (t, *J* = 6.8 Hz, 2H), 3.32 (t, *J* = 6.8 Hz, 2H), 3.20 (m, 4H), 2.64 (m, 2H), 2.57 (m, 4H), 2.29 (t, *J* = 7.4 Hz, 2H), 1.88 (m, 6H), 1.80 (m, 4H). ^13^C NMR (150 MHz, CD_3_OD): *δ*_C_ 173.7, 155.9, 150.1, 144.5, 115.5, 114.4, 50.6, 50.6, 47.9, 46.9, 34.8, 34.5, 26.9, 25.5, 25.4, 25.4, 24.1, 22.1, 22.1. The calculated HRMS (ESI) *m*/*z* for [C_19_H_27_N_3_O + H]^+^ was 314.2227, while the observed value was 314.2230. Purity: >96%.

Compound **4c** was synthesized from **4** (0.3 mmol) reacted with methyl piperidine-4-carboxylate (0.4 mmol), as described in the general procedure. The obtained compound was a yellow liquid (16 mg) with a 13.8% yield. ^1^H NMR (600 MHz, CD_3_OD): δ_H_ 7.50 (s, 1H), 4.28 (d, *J* = 14.0 Hz, 1H), 3.80 (d, *J* = 14.0 Hz, 1H), 3.61 (s, 3H), 3.19 (m, 3H), 3.08 (t, *J* = 11.4 Hz, 1H), 2.74 (t, *J* = 11.4 Hz, 1H), 2.62 (t, *J* = 6.4 Hz, 2H), 2.57 (m, 5H), 2.37 (m, 2H), 1.86 (m, 7H), 1.78 (m, 2H), 1.54 (m, 1H), 1.45 (m, 1H). ^13^C NMR (150 MHz, CD_3_OD): *δ*_C_ 176.3, 173.5, 155.9, 150.1, 144.6, 115.5, 114.4, 52.3, 50.6, 49.9, 46.1, 42.1, 41.9, 34.5, 33.5, 29.7, 29.1, 25.9, 25.5, 24.1, 22.1, 22.1. The calculated HRMS (ESI) *m*/*z* for [C_22_H_31_N_3_O_3_ + H]^+^ was 386.2438, while the observed value was 386.2443. Purity: >99%.

Compound **4d** was synthesized from **4** (0.3 mmol) reacted with methyl piperidine (0.4 mmol), as described in the general procedure. The obtained compound was a yellow liquid (35 mg) with a 35.8% yield. ^1^H NMR (600 MHz, CD_3_OD):δ_H_ 7.50 (s, 1H), 3.47 (m, 2H), 3.40 (m, 2H), 3.19 (m, 3H), 3.06 (s, 1H), 2.63 (m, 2H), 2.58 (m, 4H), 2.35 (m, 2H), 1.88 (m, 4H), 1.78 (m, 2H), 1.60 (m, 2H), 1.53 (m, 2H), 1.45 (m, 2H), 1.03 (m, 1H). ^13^C NMR (150 MHz, CD_3_OD): δ_C_ 173.3, 155.4, 150.4, 143.5, 115.6, 114.4, 50.7, 50.0, 48.0, 43.9, 34.3, 33.6, 27.6, 26.8, 26.0, 25.5, 25.4, 24.1, 22.0, 22.0. The calculated HRMS (ESI) *m*/*z* for [C_20_H_29_N_3_O + H]^+^ was 328.2383, while the observed value was 328.2388. Purity: >95%.

### 2.4. Biological Assays

#### 2.4.1. Cytotoxic Effect of the Compounds on HepG2 2.2.15 Hepatoma Cells

HepG2.2.15 cells were thawed, placed in MEM (Gibco Life Technologies, Inc., Grand Island, NY, USA) medium containing 10% FBS (Yufeng Biotechnology Co., Ltd., Guangzhou, China), 380 μg/mL G418 (Beyotime Biotechnology Co., Ltd., Shanghai, China) and 10% NEAA (Beijing Solarbio Science & Technology Co., Ltd., Beijing, China), and incubated in a cell culture incubator at 37 °C, 5% CO_2_ for 24 h. After the cells were stabilized, cells in the logarithmic phase of growth were selected for experimentation [22]. The MTT method was employed to determine the cytotoxicity of the compounds toward HepG2.2.15 cells. First, HepG2.2.15 cells were inoculated in 24-well plates at a density of 1 × 10^5^ cells/well and incubated at 37 °C, 5% CO_2_ for 24 h. The sample solution was prepared with DMSO as solvent at a master batch concentration of 200 mM, and the cells were treated with a final concentration of 0.6 mM, which has no effect on cell replication (the final DMSO concentration was ≤0.1% DMSO (*v*/*v*)) and placed in an incubator at 37 °C, 5% CO_2_. After 6 days of incubation, 50 μL of MTT solution (0.5 mg/mL in phosphate-buffered saline (PBS)) was added to each well, and the cells were incubated for an additional 4 h to produce a formazan product. The culture solution was aspirated and discarded, and 500 μL of DMSO was added to each well to fully dissolve the purple nitrile crystals generated in the cells. The absorbance at 490 nm was measured using a microplate reader (Thermo Fisher Scientific, Waltham, MA, USA). The amount of purple formazan crystal, generated by dehydrogenases in cells reducing MTT, is directly proportional to the number of living cells [23].

#### 2.4.2. Antiviral Effect of Compounds on HBV Viral Antigen Expression

The effects of all the synthesized compounds on HBeAg and HBsAg antigen secretion were determined by ELISA. HepG2.2.15 cells (1 × 10^5^ cells/well) were seeded into 24-well plates. After 24 h of incubation, the cells were treated with freshly prepared medium containing a test compound (0.6 mM) or 3TC (1 mM) and incubated at 37 °C for 6 days; the medium was refreshed with fresh drug-containing medium every 3 days. Finally, the culture medium was harvested to determine the amount of HBsAg and HBeAg secretion using an ELISA kit for HBsAg and HBeAg (Kehua Bio-Engineering Co., Ltd., Shanghai, China). Lamivudine (3TC) (Sigma, USA) was used as a positive control, and medium without a sample was used as a negative control. The absorbance values (OD) were measured at a detection wavelength of 450 nm and a reference wavelength of 630 nm using a microplate reader (Thermo Fisher Scientific, USA), and the rate of inhibition of HBeAg and HBsAg production was calculated. Each test was performed in triplicate, and the SEM (standard error of the mean) of the inhibition values varied by no more than 3% [24].

#### 2.4.3. Effects of the Compounds on HBV DNA Replication

Viral DNA was extracted from the medium using a TIANamp Virus DNA/RNA Kit (TIANGEN, Beijing, China), and qPCR was performed to determine the quantity. The forward primer 5′-GTTGCCCGTTTGTCCTCTAATTC-3′ and the reverse primer 5′-GGAGGGATACATAGAGG TTCCTT-3′ were used to detect the viral genome. The PCR reactions were performed using SYBR Green PCR master mix (Vazyme, Nanjing, China) and the primer pair noted above, with the following program: initial denaturation at 95 °C for 2 min, followed by 40 cycles of amplification at 95 °C for 15 s and annealing/extending at 60 °C for 30 s [5].

#### 2.4.4. Computational Details of Molecular Docking

The three-dimensional structure of DNA damage-binding protein 1 (DDB1) was obtained from the Uniprot database (PDB ID: 3EI1) and subjected to a series of pre-processing steps to ensure the accuracy of the molecular docking analysis. First, we used Notepad2 to remove water molecules and non-specific small organic molecules from the protein to avoid non-physiological interference. Subsequently, the protein was structurally optimized using AutoDockTools 1.5.6 (ADT), which included the follow steps: (1) adding polar hydrogen to optimize hydrogen bond interactions, thereby improving ligand-receptor binding prediction; (2) assigning partial atomic charge based on the Gasteiger method to ensure the accuracy of electrostatic interaction modeling; and (3) defining the atomic types of AutoDock force fields so that interactions such as van der Waals forces, hydrophobic interactions, and hydrogen bonds could be properly handled in the calculation process.

In the molecular docking experiment, AutoDock Vina 1.5.6 was used for calculation. This software is based on a Monte Carlo genetic algorithm, which can efficiently search the conformation space of ligands and evaluate the binding affinity by considering the score functions of spatial matching, hydrophobic effect, and electrostatic interaction. AutoDock Vina uses a strategy combining global search and local optimization to predict the optimal ligand binding posture in an iterative manner and calculates the binding free energy (unit: kcal/mol). The lower the score, the stronger the binding energy is (generally a binding energy of less than −5 kcal/mol can be regarded as stronger binding). The structures of compounds **1h**, **4a**, and **4d** were mapped using ChemBioDraw Ultra 14.0, their spatial conformations were optimized using ChemBio3D Ultra 14.0 software to minimize energy and improve docking accuracy, and finally they were saved in mol2 format for use with AutoDock Vina. During the docking process, 10 different low-energy conformations were generated for each compound, and the optimal binding mode was screened for using an RMSD threshold of less than 5 Å. In addition, docking grid parameters were automatically generated according to the binding site, and the size was set to 20 × 20 × 20 Å to cover the active site and ensure docking flexibility.

## 3. Results

### 3.1. Chemistry

**Scheme 1:**
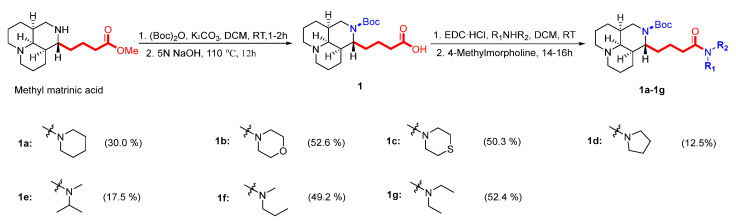
Synthesis of the first set of target compounds, **1a**–**1g**.

A total of 23 matrinic acid derivatives were synthesized with commercially available matrine as the starting material, as depicted in Figure 3. As reported earlier [25], intermediate **1** was obtained following a two-step procedure including 16 *N*-*t*-butyloxy (Boc) protection and hydrolysis of methyl matrinic acid. Subsequently, intermediate **1** was reacted with different secondary amines to yield different matrinic amides (**1a**–**1g**), as depicted in Scheme 1. In addition, **1g** was subjected to acid hydrolysis to obtain **1h** [26].

**Scheme 2:**
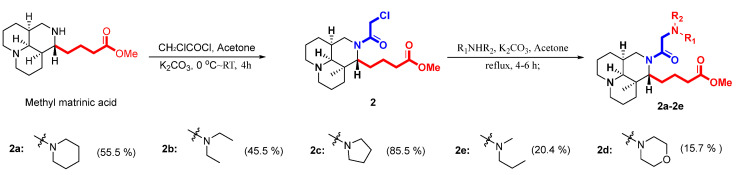
Synthesis of the second set of target compounds, **2a**–**2e**.

As depicted in Scheme 2, the key intermediate of compound **2** was obtained by condensation of methyl matrinic acid with chloroacetyl chloride under slightly alkaline (K_2_CO_3_) conditions, which facilitates amidation with relatively high yields and purity [27,28]. Finally, different secondary amines directly connected to intermediate **2** at the *N*_16_ position were used as nucleophilic reagents to transform methyl matrinic acid into the desired products **2a**–**2e**.

**Scheme 3:**
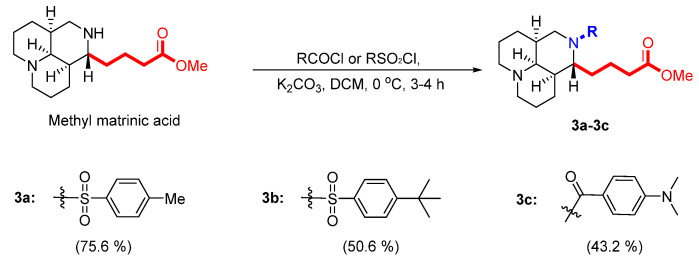
Synthesis of the third set of target compounds, **3a**–**3c**.

As depicted in Scheme 3, target compounds **3a**–**3c** were obtained by acylation or sulfonylation of methyl matrinic acid.

**Scheme 4:**
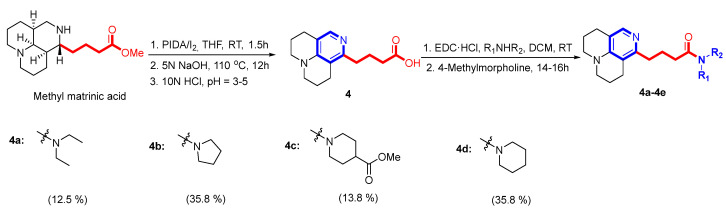
Synthesis of the third set of target compounds, **4a**–**4d**.

A three-step synthesis protocol starting with methyl matrinic acid was used to obtain compound **4**, as shown in Scheme 4 [29]. Subsequently, compound **4** was reacted with different secondary amines to yield different matrinic amides (**4a**–**4d**), as depicted in Scheme 4 [30].

### 3.2. Biological Activity

All of the synthesized compounds were evaluated for their in vitro anti-HBV activity (HBsAg, HBeAg, DNA) and cytotoxicity in HepG2.2.15 cells (human HBV transgenic hepatocellular carcinoma cells) using standard ELISA, PCR, and MTT methods. The concentration of each compound required for 50% inhibition of HBsAg secretion, HBeAg secretion, and HBV DNA replication was defined as its IC_50_. Lamivudine (3TC) was used as positive control. The effects of all 23 synthesized compounds on the proliferation of HepG 2.2.15 cells were tested in vitro by MTT assay. The results showed that concentrations of all compounds below 0.4 mM had no significant toxicity toward HepG2.2.1.5 cells for 9 days (Supplementary Table S1).

To investigate the inhibitory effect of all of the synthesized compounds on the production of the HBsAg and HBeAg by HepG2.2.15 cells, the supernatant was collected after treatment with the test compounds at a concentration of 0.2 mM for 9 days, and the titers of HBsAg and HBeAg were determined using ELISA kits, with 3TC (0.2 mM) used as a positive control. As shown in Table 1, the alkaloids exhibited potent activity against HBV, with higher potency against secretion of HBeAg than against secretion of HBsAg. Eleven of the compounds significantly inhibited HBeAg secretion by more than 30%, which suggests that these natural products are more potent than the positive control lamivudine (3TC, 15.6 ± 3.0%).

**Figure 4:**
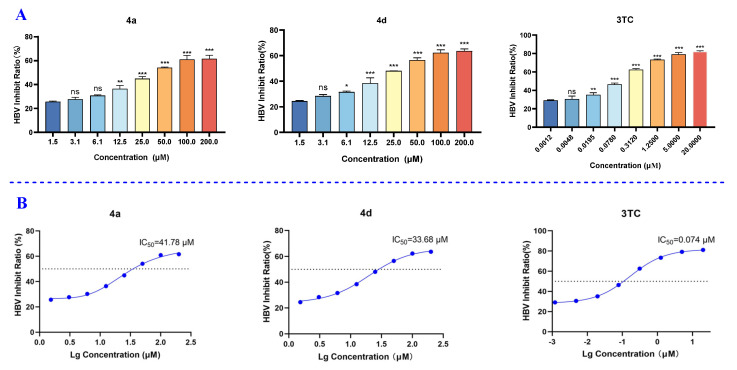
The inhibitory effects of compounds **4a** and **4d** and 3TC on HBV DNA replication. (**A**) Inhibition rates of **4a**, **4d**, and 3TC at the different concentrations. (**B**) IC_50_ values of **4a**, **4d**, and 3TC. Data are presented as mean ± SD of three experiments. ns means no significant, * *p* < 0.05, ** *p* < 0.01, and *** *p* < 0.001.

The inhibitory effect of the target compounds on HBV DNA replication were evaluated in the HepG2.2.15 cell line using 3TC as a positive control. After treatment of the cells with the test compounds at a concentration of 50 μM for 9 days, extracellular HBV DNA levels were quantified by qPCR. As shown in Table 1, some compounds exhibited mild inhibitory activity against HBV DNA replication. Of these compounds, **4a** and **4d** showed the most potent in vitro anti-HBV DNA activity, with inhibition levels of 57.5 ± 1.0% and 50.5 ± 1.1%, respectively, at a concentration of 50 μM. Compounds **1b**, **1d**, and **4** also exhibited significant efficacy against HBV DNA replication, at 47.2 ± 2.1%, 39.6 ± 1.2%, 44.1 ± 1.0%, and 36.8 ± 1.9%, respectively, at a concentration of 50 μM. Compounds **4a** and **4d** were further evaluated in HepG2.2.15 cells, and their IC_50_ values were 41.87 and 33.68 μM, respectively. The IC_50_ value of the positive control 3TC was 0.074 μΜ (Figure 4).

**Figure 5:**
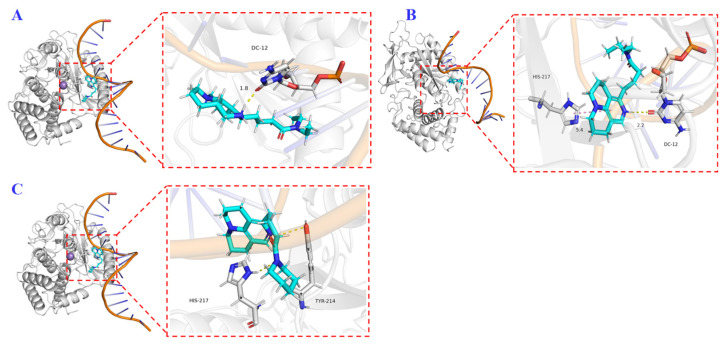
3D binding model of **1h** ((**A**), cyan), **4a** ((**B**), cyan), and **4d** ((**C**), cyan) with DDB1–DNA complexes (white and purple). The key residues are shown as white sticks. H-bonds are shown as yellow dashed lines. The docking scores for **1h**, **4a**, and **4d** were −4.881 kcal⋅mol^−1^, −5.474 kcal⋅mol^−1^, and −5.318 kcal⋅mol^−1^, respectively.

Molecular modeling studies can lead to an understanding of the detailed binding interaction between enzyme and ligand [31]. In this study, the molecular docking technique was used to investigate the binding patterns and interaction mechanisms of compounds **1h**, **4a**, and **4d** with DDB1–DNA complexes. The docking results are shown in Figure 5: DDB1–DNA complexes are represented by white and purple cartoons, compounds **1h**, **4a**, and **4d** are represented by cyan rods, and key amino acid residues are represented by white rods. The results show that the compound had a strong ability to bind DDB1–DNA complexes. Compound **1h** formed a stable hydrogen bond with DC-12, the 12th base on the DNA sequence, compound **4a** formed two hydrogen bonds with DC-12 and the protein (His-217) at the same time, and compound **4d** also formed two hydrogen bonds, with the protein (His-217) and with the DNA sequence (Tyr-214). These interactions are essential for maintaining the conformational stability of DDB1–DNA complexes and may affect biological function by interfering with DDB1–DNA interactions.

## 4. Discussion

In this study, 23 methyl matrinic acid derivatives were synthesized, and their inhibitory activities against HBsAg secretion, HBeAg secretion, and HBV DNA replication in the HepG2.2.15 cell line were determined. Synthesis of the first through third series of compounds was achieved solely through hydrolysis, amidation, and nucleophilic substitution. However, synthesis of intermediate compound **4** was relatively difficult, as it could not be directly generated through one-step oxidation using matrinic acid as a substrate. We found that, if the carboxyl group was not converted into methyl esters, the oxidation products were very complex, and the target product could be detected but not purified. Therefore, the yields of intermediates **1** and **2** were relatively higher, while the yield of intermediate **4** was less than 40%. In addition, the synthesis of key intermediate **4** could only be scaled up to 1.0 mmol before the yield significantly decreased.

After successfully synthesizing 23 target compounds, we evaluated their anti-HBV activity, along with that of matrine, matrinic acid, and methyl matrinic acid. Firstly, the inhibitory effects of these compounds on the production of HBsAg and HBeAg by HepG2.2.15 cells were evaluated by ELISA. In terms of the effect of the compound on HBsAg, the activity of the template substrate methyl matrinic acid was comparable to that of the positive control (3TC). Among the synthesized target products, except for compounds **2b**, **2c**, and **4**, whose activities increased by more than two times, the activities of most of the compounds did not significantly improve, and seven compounds even completely lost their activity. The significant decrease in the activity of compounds **1c**–**1g** may be attributed to the introduction of a strong electron-withdrawing group at *N*-12. For HBeAg, over half of the synthesized compounds exhibited an activity level that was two times higher than that of 3TC. Interestingly, the activity of some of the compounds with strong electron-withdrawing groups at the *N*-12 position significantly improved, which is exactly the opposite of the result for HBsAg. Among them, the activity of compounds **3a**–**3c** was significantly improved. In general, the newly designed derivatives displayed moderate inhibitory activity against the secretion of HBsAg and HBeAg compared with the lead compound (methyl matrinic acid) and the positive control (3TC).

Subsequently, the inhibitory effect on HBV DNA replication of all of the compounds was evaluated by qPCR in the HepG2.2.15 cell lines. The primary anti-HBV DNA replication activities in HepG2.2.15 cells of the target compounds, lead compound (methyl matrinic acid), and positive control (3TC) at 50 μM are shown in Table 1. Of these compounds, **4a** and **4d** showed the most potent in vitro anti-HBV DNA activity, and compounds **1b**, **1d**, and **4** also exhibited moderate efficacy. From the perspective of the structure of the compounds, the compounds with significantly improved activity all formed new amide bonds at C-16. The C ring of compounds **4a** and **4d** is an aromatic pyridine ring formed through oxidation. In general, the newly designed derivatives with pyridine at the C ring and amide bonds at C-16 displayed significant inhibitory activity against the replication of HBV DNA. These results provide valuable information for further modification to find more potent anti-HBV inhibitors.

HBV regulatory protein X (HBx) was recently found to promote transcription of cccDNA and degradation of Smc5/6 by interacting with the host protein DDB1. Here, this protein–protein interaction was considered as a new molecular target for HBV treatment [32,33]. Therefore, 3D binding analysis of **4a** and **4d** with DDB1–DNA complexes was carried out using a molecular docking technique. The results showed that compounds **4a** and **4d** formed two hydrogen bonds with DDB1–DNA complexes, and the docking scores of **4a** and **4d** were −5.474 and −5.318 kcal/mol, respectively. So, it is reasonable that the inhibition values of these two compounds are very close. We found that the structure of compound **1h** was most similar to that of compound **4a** but lacked the aromatic ring. The activity of compound **4** was significantly stronger than that of **1h**, so we conducted molecular docking experiments on **1h**. The results indicate that the interaction between compound **1h** and DDB1–DNA complexes was weaker than that of **4a**, both in terms of the number of hydrogen bonds (only one in **1h**) and docking score (−4.881 kcal⋅mol^−1^ for **1h**). Overall, these molecular docking studies not only reveal the binding patterns of compounds **1h**, **4a**, and **4d**, but also provide a theoretical basis for further optimizing their structure, improving binding affinity, and exploring their potential applications in targeted DDB1–DNA complexes.

## 5. Conclusions

In summary, the present work is an extension of our ongoing efforts toward the development and identification of new molecules with anti-HBV activity. In the present study, we found that, if a strong electron-withdrawing group was introduced at *N*-12 of the lead compound (methyl matrinic acid), it greatly enhanced the compound’s ability to inhibit HBeAg secretion, but correspondingly, its ability to inhibit HBsAg secretion decreased or was even lost. Furthermore, oxidation of the C ring of the lead compound to an aromatic pyridine ring enhanced their ability to inhibit anti-HBV DNA replication. Based on the fact that **1b** and **1d** still exhibited significant inhibitory effects on HBV DNA replication in the absence of a pyridine ring, we propose that subsequent synthesis strategies should focus on the introduction of a strong electron-withdrawing group at *N*-16 and the formation of a new amide bond at C-15. In summary, our results provide insight into the rational design of matrinic acid derivatives and offer promising avenues for the future development of anti-HBV agents.

## Figures and Tables

**Table 1:** Inhibitory activity of compounds against HBsAg, HBeAg, and HBV DNA levels.

Compound	HBsAg (% at 0.2 mM)	HBeAg (% at 0.2 mM)	HBV DNA (% at 50 μM)
**1**	2.25 ± 1.2	19.87 ± 0.4	12.4 ± 0.7
**1a**	10.0 ± 1.3	42.7 ± 1.8	31.7 ± 2.2
**1b**	16.6 ± 2.3	38.3 ± 1.1	47.2 ± 2.1
**1c**	− *^a^*	20.7± 0.3	21.1 ± 2.3
**1d**	− *^a^*	21.7 ± 0.8	39.6 ± 1.2
**1e**	− *^a^*	56.7 ± 0.9	19.6 ± 2.5
**1f**	− *^a^*	46.4 ± 1.2	− *^a^*
**1g**	− *^a^*	42.8 ± 0.4	16.0 ± 3.7
**1h**	13.6 ± 0.2	39.0 ± 1.6	27.0 ± 2.2
**2**	9.9 ± 2.1	6.43± 0.3	24.3 ± 0.5
**2a**	11.2 ± 1.2	− *^a^*	30.6 ± 2.4
**2b**	45.3 ± 0.1	6.0 ± 1.4	28.1 ± 0.8
**2c**	40.2 ± 0.8	2.9 ± 1.8	10.5 ± 2.2
**2d**	19.5 ± 1.2	11.4 ± 0.8	11.4 ± 1.5
**2e**	− *^a^*	40.1 ± 2.8	18.2 ± 2.8
**3a**	9.6 ± 0.2	43.0 ± 1.1	12.8 ± 1.9
**3b**	− *^a^*	43.6 ± 1.2	28.1 ± 1.9
**3c**	28.1 ± 2.2	51.9 ± 1.8	29.1 ± 0.9
**4**	36.7 ± 1.3	15.3 ± 0.2	44.1 ± 1.0
**4a**	30.1 ± 1.1	18.9 ± 1.0	54.1 ± 0.3
**4b**	30.4 ± 1.0	20.5 ± 2.0	26.5 ± 1.8
**4c**	21.6 ± 1.8	3.6 ± 1.3	15.9 ± 3.2
**4d**	24.5 ± 0.7	33.7 ± 1.3	56.6 ± 1.3
Matrine	12.2 ± 1.5	21.7 ± 0.3	36.8 ± 1.9
Matrinic acid	5.4 ± 2.0	4.2 ± 1.3	− *^a^*
Methyl matrinic acid	20.9 ± 1.1	2.6 ± 1.1	37.0 ± 1.1
3TC *^b^*	16.7 ± 1.7	15.6 ± 3.0	83.2 ± 1.1

*^a^* No inhibitory effect. *^b^* 3TC (lamivudine) was used as a positive control.

## Data Availability

The datasets used and analyzed during the current study are available from the corresponding author upon reasonable request.

## Supplementary Materials

The following supporting information can be downloaded at: https://www.mdpi.com/article/10.3390/biom15030436/s1, File S1: NMR spectra and HRESIMS for compounds **1**–**4**, **1a**–**1h**, **2a**–**2e**, **3a**–**3c**, and **4a**–**4d**.
