# Supplementary material for: Design, Synthesis, and Bioevaluation of Matrine Derivatives as Potential Anti–Hepatitis B Virus Agents
Source: Biomolecules. 2025 Mar 18;15(3):436. doi: 10.3390/biom15030436 (PMC11940400; doi:10.3390/biom15030436)
Supplement: Supplementary file 1 [file biomolecules-15-00436-s001.zip › biomolecules-3500549-supplementary.pdf]

Supporting Information

Design, Synthesis, and Bioevaluation of Matrine Derivatives as Potential Anti–Hepatitis B Virus Agents

Ting-Ting Liu[†], Meng-Fan Xie[†], Xin Liu, Rong-Tao Li, Yao Bai*, and Zhi-Jun Zhang*

Faculty of Life Science and Technology, Kunming University of Science and Technology, Kunming 650500, Yunnan, PR China

*Correspondence. E-mail address: baiy@kust.edu.cn (Y.B.); zhangzj@kust.edu.cn (Z.-J.Z.)
[†]These authors contributed equally to this work.

Table of Contents

Table S1. HepG 2.2.15 cell viability assay treatment with different compounds at 0.4 mM for 9 days 4
Figure S1. HRESIMS analysis of 1 5
Figure S2. ^{1}H NMR spectrum (600 MHz, CD_3OD) of 1 6
Figure S3. ^{13}C NMR spectrum (150 MHz, CD_3OD) of 1 6
Figure S4. HRESIMS analysis of 2 7
Figure S5. ^{1}H NMR spectrum (600 MHz, $CDCl_3$) of 2 8
Figure S6. ^{13}C NMR spectrum (150 MHz, $CDCl_3$) of 2 8
Figure S7. ^{1}H NMR spectrum (600 MHz, CD_3OD) of 4 9
Figure S8. ^{13}C NMR spectrum (150 MHz, CD_3OD) of 4 9
Figure S9. HRESIMS analysis of 1a 10
Figure S10. ^{1}H NMR spectrum (600 MHz, CD_3OD) of 1a 11
Figure S11. ^{13}C NMR spectrum (150 MHz, CD_3OD) of 1a 11
Figure S12. HRESIMS analysis of 1b 12
Figure S13. ^{1}H NMR spectrum (600 MHz, CD_3OD) of 1b 13
Figure S14. ^{13}C NMR spectrum (150 MHz, CD_3OD) of 1b 13
Figure S15. HRESIMS analysis of 1c 14
Figure S16. ^{1}H NMR spectrum (600 MHz, CD_3OD) of 1c 15
Figure S17. ^{13}C NMR spectrum (150 MHz, CD_3OD) of 1c 15
Figure S18. HRESIMS analysis of 1d 16
Figure S19. ^{1}H NMR spectrum (600 MHz, CD_3OD) of 1d 17
Figure S20. ^{13}C NMR spectrum (150 MHz, CD_3OD) of 1d 17
Figure S21. HRESIMS analysis of 1e 18
Figure S22. ^{1}H NMR spectrum (600 MHz, CD_3OD) of 1e 19
Figure S23. ^{13}C NMR spectrum (150 MHz, CD_3OD) of 1e 19
Figure S24. HRESIMS analysis of 1f 20
Figure S25. ^{1}H NMR spectrum (600 MHz, CD_3OD) of 1f 21
Figure S26. ^{13}C NMR spectrum (150 MHz, CD_3OD) of 1f 21
Figure S27. HRESIMS analysis of 1g 22
Figure S28. ^{1}H NMR spectrum (600 MHz, CD_3OD) of 1g 23
Figure S29. ^{13}C NMR spectrum (150 MHz, CD_3OD) of 1g 23
Figure S30. HRESIMS analysis of 1h 24
Figure S31. ^{1}H NMR spectrum (600 MHz, CD_3OD) of 1h 25
Figure S32. ^{13}C NMR spectrum (150 MHz, CD_3OD) of 1h 25
Figure S33. HRESIMS analysis of 2a 26
Figure S34. ^{1}H NMR spectrum (600 MHz, CD_3OD) of 2a 27
Figure S35. ^{13}C NMR spectrum (150 MHz, CD_3OD) of 2a 27
Figure S36. HRESIMS analysis of 2b 28
Figure S37. ^{1}H NMR spectrum (600 MHz, CD_3OD) of 2b 29
Figure S38. ^{13}C NMR spectrum (150 MHz, CD_3OD) of 2b 29
Figure S39. HRESIMS analysis of 2c 30
Figure S40. ^{1}H NMR spectrum (600 MHz, CD_3OD) of 2c 31
Figure S41. ^{13}C NMR spectrum (150 MHz, CD_3OD) of 2c 31
Figure S42. HRESIMS analysis of 2d 32
Figure S43. ^{1}H NMR spectrum (600 MHz, CD_3OD) of 2d 33
Figure S44. ^{13}C NMR spectrum (150 MHz, CD_3OD) of 2d 33
Figure S45. HRESIMS analysis of 2e 34
Figure S46. ^{1}H NMR spectrum (600 MHz, CD_3OD) of 2e 35

Figure S47. ^{13}C NMR spectrum (150 MHz, CD_3OD) of 2e 35
Figure S48. HRESIMS analysis of 3a 36
Figure S49. ^{1}H NMR spectrum (600 MHz, $CDCl_3$) of 3a 37
Figure S50. ^{13}C NMR spectrum (150 MHz, $CDCl_3$) of 3a 37
Figure S51. HRESIMS analysis of 3b 38
Figure S52. ^{1}H NMR spectrum (600 MHz, $CDCl_3$) of 3b 39
Figure S53. ^{13}C NMR spectrum (150 MHz, $CDCl_3$) of 3b 39
Figure S54. HRESIMS analysis of 3c 40
Figure S55. ^{1}H NMR spectrum (600 MHz, CD_3OD) of 3c 41
Figure S56. ^{13}C NMR spectrum (150 MHz, CD_3OD) of 3c 41
Figure S57. HRESIMS analysis of 4a 42
Figure S58. ^{1}H NMR spectrum (600 MHz, CD_3OD) of 4a 43
Figure S59. ^{13}C NMR spectrum (150 MHz, CD_3OD) of 4a 43
Figure S60. HRESIMS analysis of 4b 44
Figure S61. ^{1}H NMR spectrum (600 MHz, CD_3OD) of 4b 45
Figure S62. ^{13}C NMR spectrum (150 MHz, CD_3OD) of 4b 45
Figure S63. HRESIMS analysis of 4c 46
Figure S64. ^{1}H NMR spectrum (600 MHz, CD_3OD) of 4c 47
Figure S65. ^{13}C NMR spectrum (150 MHz, CD_3OD) of 4c 47
Figure S66. HRESIMS analysis of 4d 48
Figure S67. ^{1}H NMR spectrum (600 MHz, CD_3OD) of 4d 49
Figure S68. ^{13}C NMR spectrum (150 MHz, CD_3OD) of 4d 49
Figure S69. NMR purity of compound 1 (> 99%) 50
Figure S70. NMR purity of compound 2 (> 95%) 50
Figure S71. NMR purity of compound 4 (> 99%) 51
Figure S72. NMR purity of compound 1a (> 93%) 51
Figure S73. NMR purity of compound 1b (> 99%) 52
Figure S74. NMR purity of compound 1c (> 98%) 52
Figure S75. NMR purity of compound 1c (> 92%) 53
Figure S76. NMR purity of compound 1d (> 95%) 54
Figure S77. NMR purity of compound 1f (> 96%) 54
Figure S78. NMR purity of compound 1g (> 93%) 55
Figure S79. NMR purity of compound 1h (> 97%) 55
Figure S80. NMR purity of compound 2a (> 97%) 55
Figure S81. NMR purity of compound 2b (> 97%) 56
Figure S82. NMR purity of compound 2c (> 97%) 56
Figure S83. NMR purity of compound 2d (> 92%) 57
Figure S84. NMR purity of compound 2e (> 90%) 57
Figure S85. NMR purity of compound 3a (> 97%) 58
Figure S86. NMR purity of compound 3b (> 97%) 58
Figure S87. NMR purity of compound 3c (> 92%) 59
Figure S88. NMR purity of compound 4a (> 94%) 59
Figure S89. NMR purity of compound 4b (> 96%) 60
Figure S90. NMR purity of compound 4c (> 99%) 60
Figure S91. NMR purity of compound 4d (> 95%) 61
Figure S92. NMR purity of compound 4e (> 95%) 61

Table S1. HepG 2.2.15 cell viability assay treatment with different compounds at 0.4 mM for 9 days

Compound	Cell viability (%)	Compound	Cell viability (%)
**1**	96.3 ± 3.4	**2d**	101.4 ± 0.6
**1a**	100.1 ± 1.2	**2e**	91.1 ± 2.3
**1b**	98.2 ± 1.2	**3a**	84.3 ± 1.0
**1c**	94.2 ± 0.7	**3b**	94.8 ± 2.5
**1d**	96.3 ±1.4	**3c**	94.1 ± 3.2
**1e**	100.5 ± 1.8	**4**	92.0 ± 2.1
**1f**	101.6 ± 1.7	**4a**	91.9± 0.6
**1g**	96.7 ± 1.1	**4b**	100.2 ± 0.1
**1h**	98.5 ± 0.8	**4c**	104.7 ± 2.2
**2**	96.5 ± 0.7	**4d**	95.4 ± 2.6
**2a**	99.0 ± 0.6	Matrine	99.8 ± 0.8
**2b**	98.1 ± 1.1	Matrinic acid	92.1 ± 2.1
**2c**	90.8 ± 0.8	Methyl matrinic acid	97.0 ± 1.2

Qualitative Analysis Report

**Data Filename**	D.d	**Sample Name**	BOC
**Sample Type**	Sample	**Position**	P1-B7
**Instrument Name**	Instrument 1	**User Name**	
**Acq Method**	20200905-HRMS(+).m	**Acquired Time**	2024/4/14 13:34:25
**IRM Calibration Status**	Success	**DA Method**	Default.m
**Comment**			

**Sample Group**		**Info.**	
**Stream Name**	LC 1	**Acquisition SW Version**	6200 series TOF/6500 series Q-TOF B.06.01 (B6172 SP1)

User Spectra

Fragmentor Voltage	Collision Energy	Ionization Mode
175	0	ESI

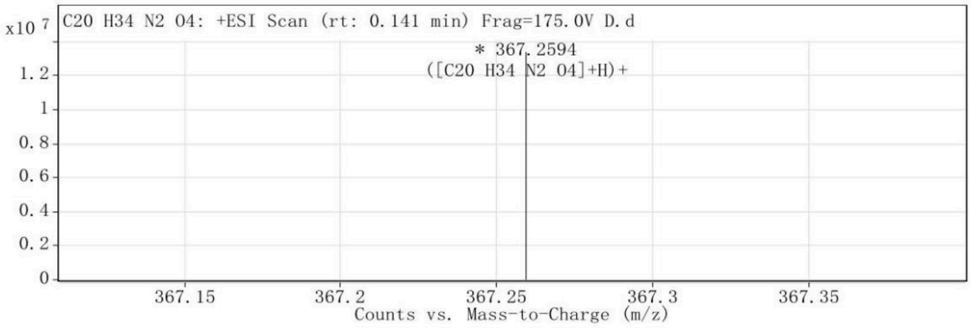

Peak List

m/z	z	Abund	Formula	Ion
267.2072	1	244865.75		
311.1965	1	2912696.5		
311.3466		148323.31		
312.2004	1	558013.75		
367.2594	1	13394097	C20 H34 N2 O4	(M+H)+
367.4207	1	765636.31		
368.2625	1	3128151	C20 H34 N2 O4	(M+H)+
368.4234	1	207869.83		
369.2661	1	483933.72	C20 H34 N2 O4	(M+H)+
755.4931	1	333918.09		

Formula Calculator Element Limits

Element	Min	Max
C	3	60
H	0	120
O	0	30
N	0	30
S	0	5
Cl	0	3

Formula Calculator Results

Formula	Best	Mass	Tgt Mass	Diff (ppm)	Ion Species	Score
C20 H34 N2 O4	TRUE	366.2521	366.2519	-0.63	C20 H35 N2 O4	99.73

--- End Of Report ---

Agilent Technologies Page 1 of 1 Printed at: 21:46 on: 2024/11/17

Figure S1. HRESIMS analysis of **1**

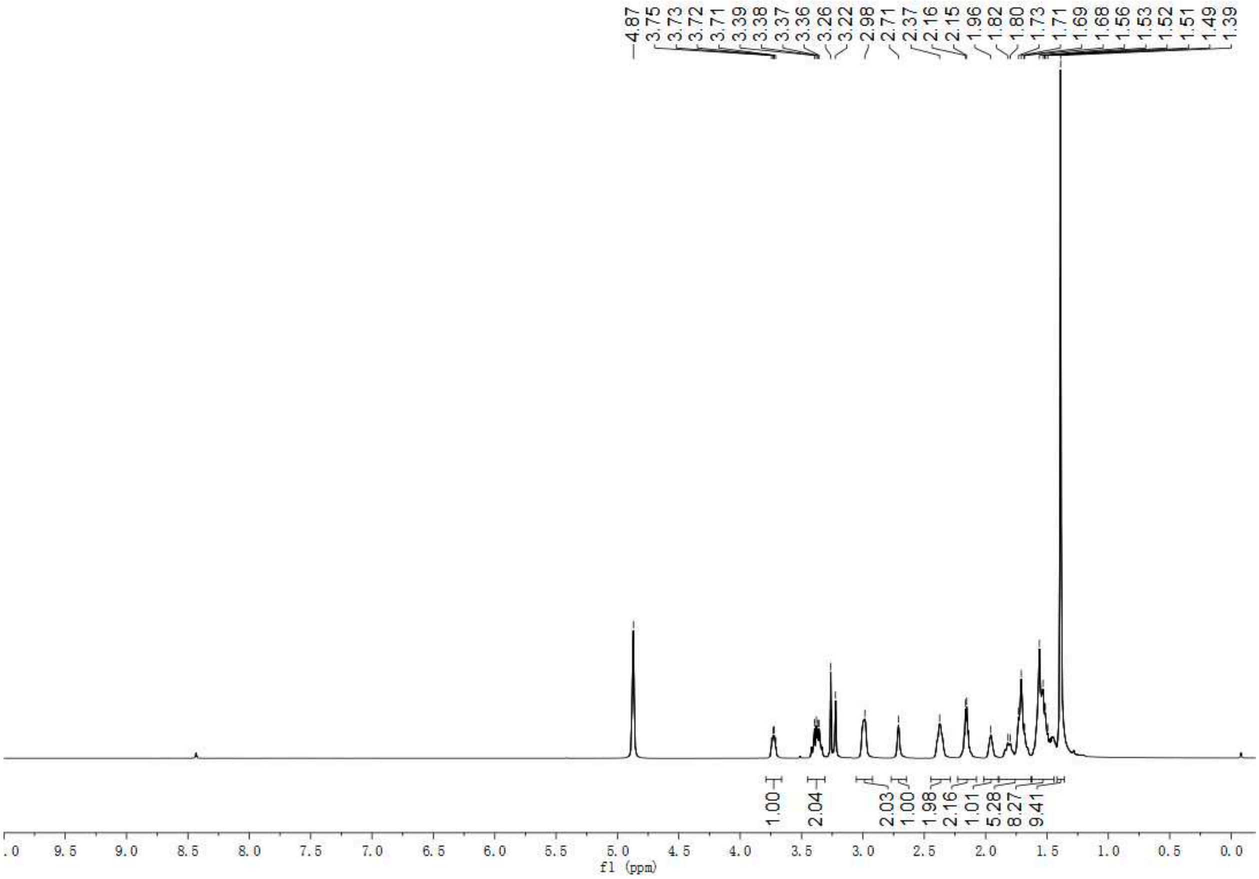

Figure S2. ^{1}H NMR spectrum (600 MHz, CD_3OD) of **1**

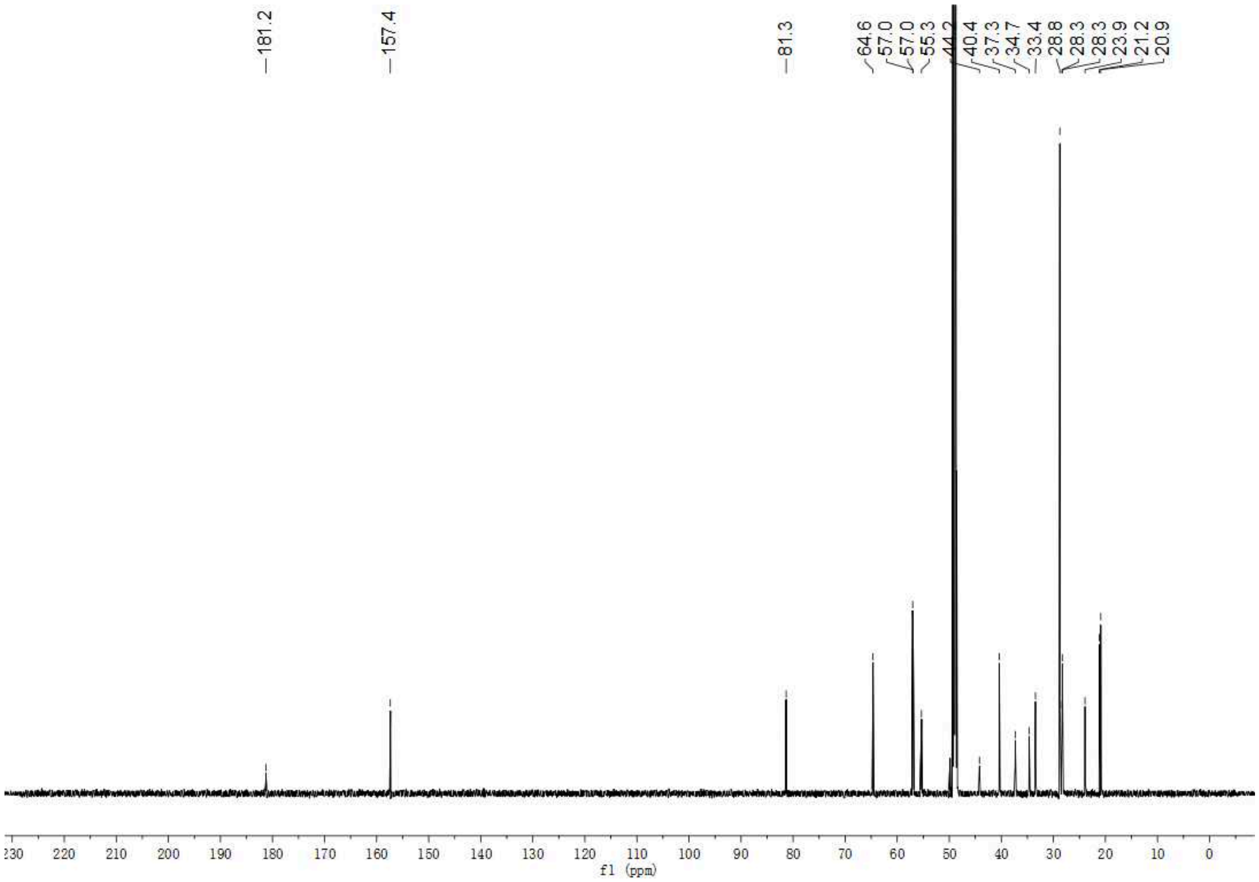

Figure S3. ^{13}C NMR spectrum (150 MHz, CD_3OD) of **1**

Qualitative Analysis Report

**Data Filename**	B.d	**Sample Name**	CL
**Sample Type**	Sample	**Position**	P1-B8
**Instrument Name**	Instrument 1	**User Name**	
**Acq Method**	20200905-HRMS(+).m	**Acquired Time**	2024/4/14 13:37:13
**IRM Calibration Status**	Success	**DA Method**	Default.m
**Comment**			

**Sample Group**		**Info.**	
**Stream Name**	LC 1	**Acquisition SW Version**	6200 series TOF/6500 series Q-TOF B.06.01 (B6172 SP1)

User Spectra

Fragmentor Voltage	Collision Energy	Ionization Mode
175	0	ESI

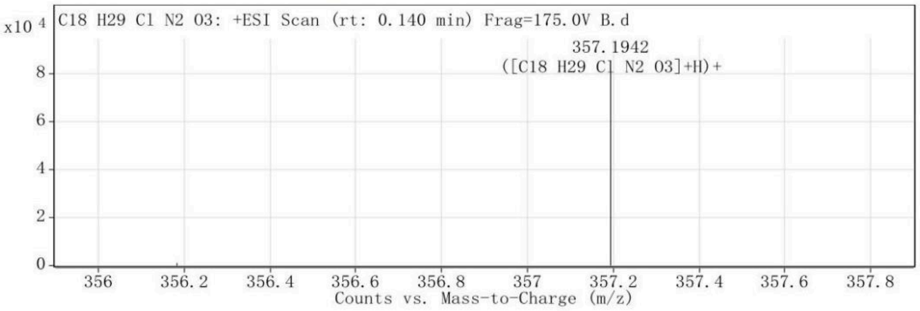

Peak List

m/z	z	Abund
249.1964	1	289192.34
281.2228	1	294338.78
339.2278	1	4221641
340.2316	1	786904.19
415.1994	1	4549054.5
416.204	1	959614.63
417.1976	1	1528003.63
418.2011	1	346652.53
473.2058	1	743235.81
475.2035	1	254981.94

Formula Calculator Element Limits

Element	Min	Max
C	3	60
H	0	120
O	0	30
N	0	30
S	0	5
Cl	0	3

Formula Calculator Results

Formula	Best	Mass	Tgt Mass	Diff (ppm)	Ion Species	Score
C18 H29 Cl N2 O3	TRUE	356.1869	356.1867	-0.66	C18 H30 Cl N2 O3	99.55

--- End Of Report ---

Figure S4. HRESIMS analysis of **2**

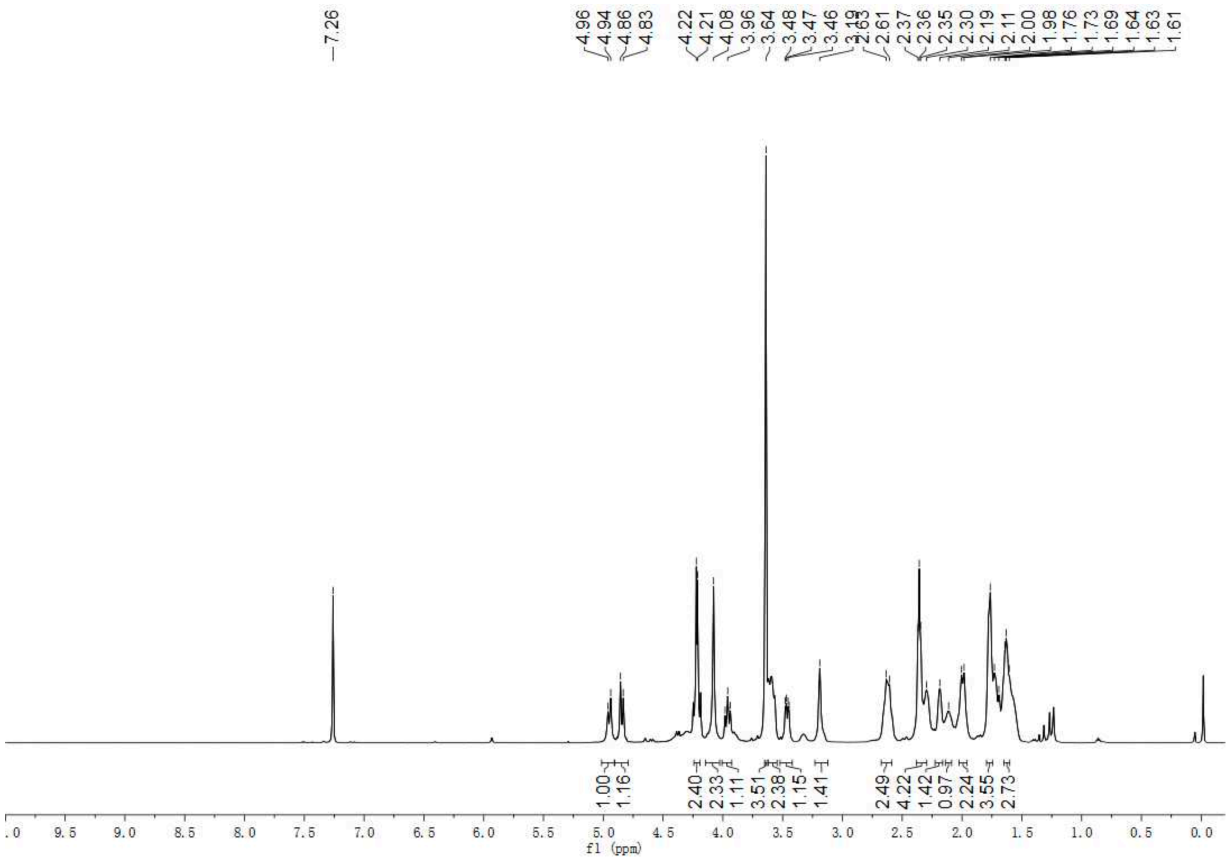

Figure S5. ^{1}H NMR spectrum (600 MHz, $CDCl_3$) of **2**

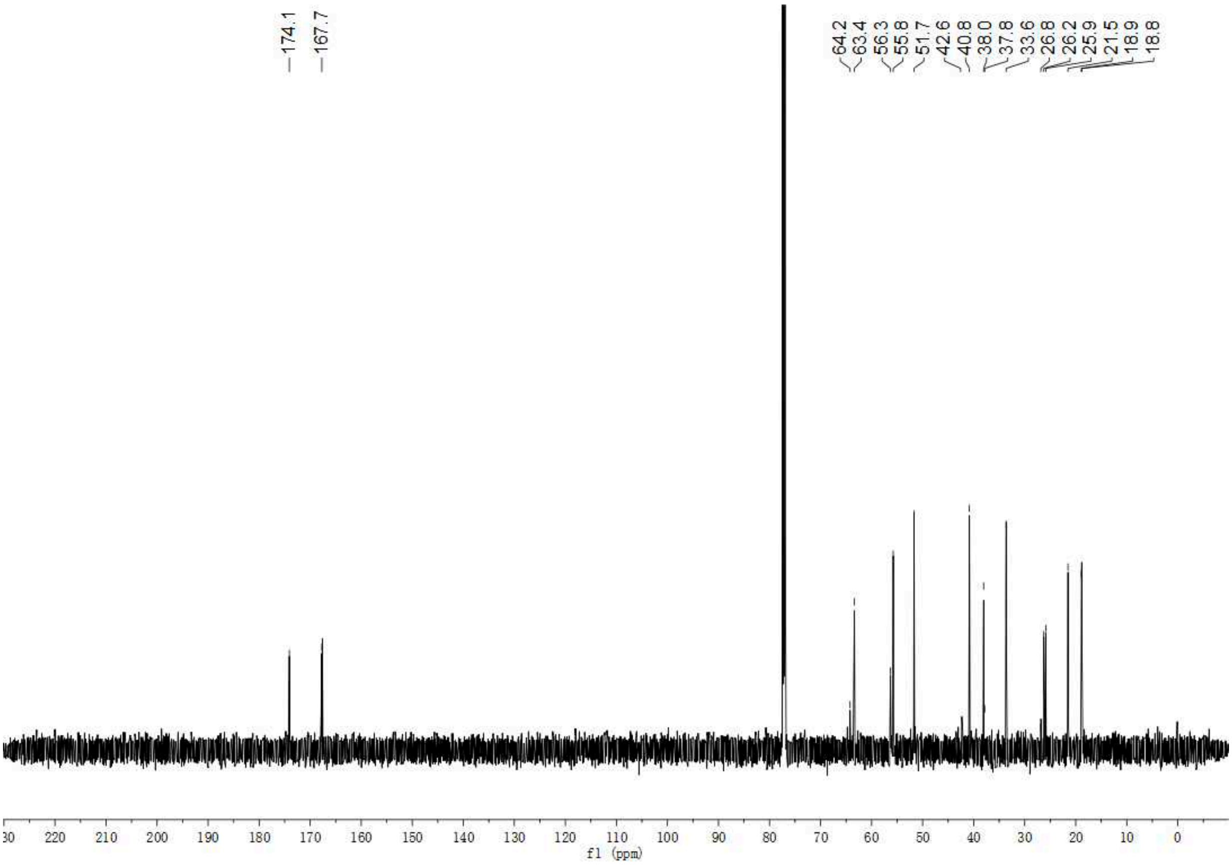

Figure S6. ^{13}C NMR spectrum (150 MHz, $CDCl_3$) of **2**

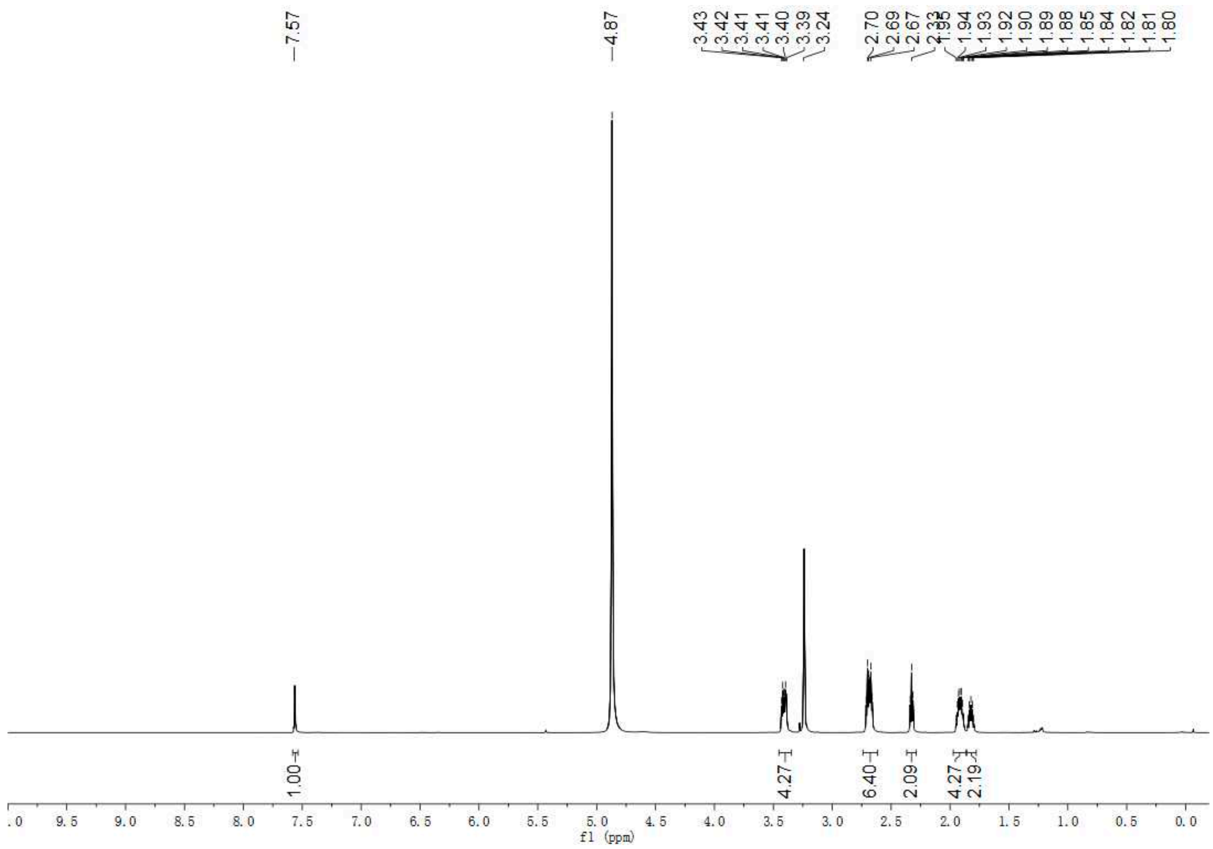

Figure S7. ^{1}H NMR spectrum (600 MHz, CD_3OD) of **4**

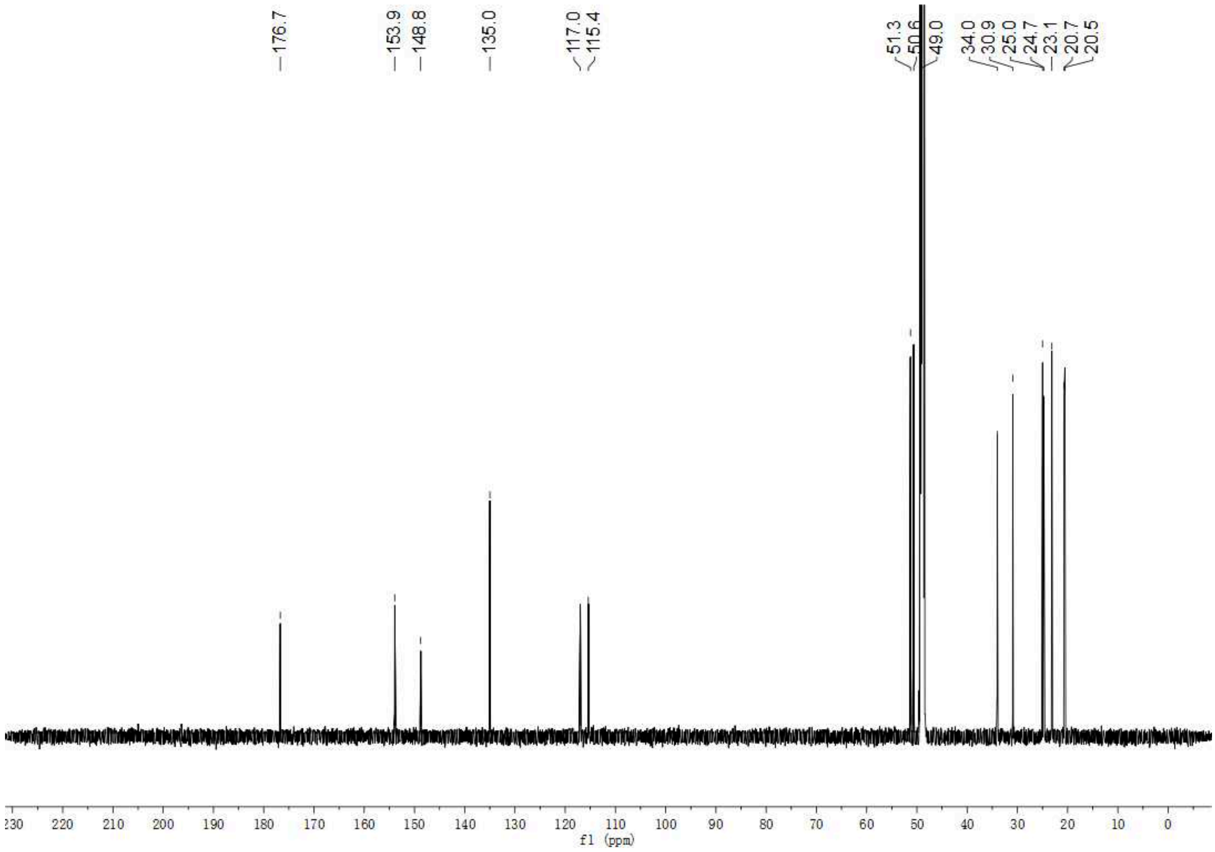

Figure S8. ^{13}C NMR spectrum (150 MHz, CD_3OD) of **4**

Qualitative Analysis Report

**Data Filename**	D1.d	**Sample Name**	D1
**Sample Type**	Sample	**Position**	P1-F6
**Instrument Name**	Instrument 1	**User Name**	
**Acq Method**	20240114-HRMS-Pos-1.5min-5%H2O.m	**Acquired Time**	2024/4/14 12:34:56
**IRM Calibration Status**	Success	**DA Method**	Default.m
**Comment**			

**Sample Group**		**Info.**	
**Stream Name**	LC 1	**Acquisition SW Version**	6200 series TOF/6500 series Q-TOF B.06.01 (B6172 SP1)

User Spectra

Fragmentor Voltage	Collision Energy	Ionization Mode
175	0	ESI

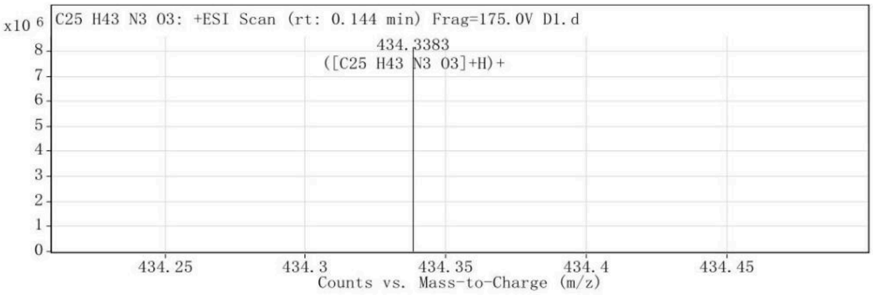

Peak List

m/z	z	Abund	Formula	Ion
325.2132	1	365672.5		
334.287	1	805643.94		
381.2758	1	1500082.13		
382.2792	1	370692.5		
432.3236	1	498368.47		
434.3383	1	8166243.5	C25 H43 N3 O3	(M+H)+
434.5123	1	417704.19		
435.3416	1	2297420.25	C25 H43 N3 O3	(M+H)+
436.3456	1	402418.81	C25 H43 N3 O3	(M+H)+
472.2949	1	581149.94		

Formula Calculator Element Limits

Element	Min	Max
C	3	60
H	0	120
O	0	30
N	0	30
S	0	5
Cl	0	3

Formula Calculator Results

Formula	Best	Mass	Tgt Mass	Diff (ppm)	Ion Species	Score
C25 H43 N3 O3	TRUE	433.3311	433.3304	-1.57	C25 H44 N3 O3	98.61

--- End Of Report ---

Figure S9. HRESIMS analysis of **1a**

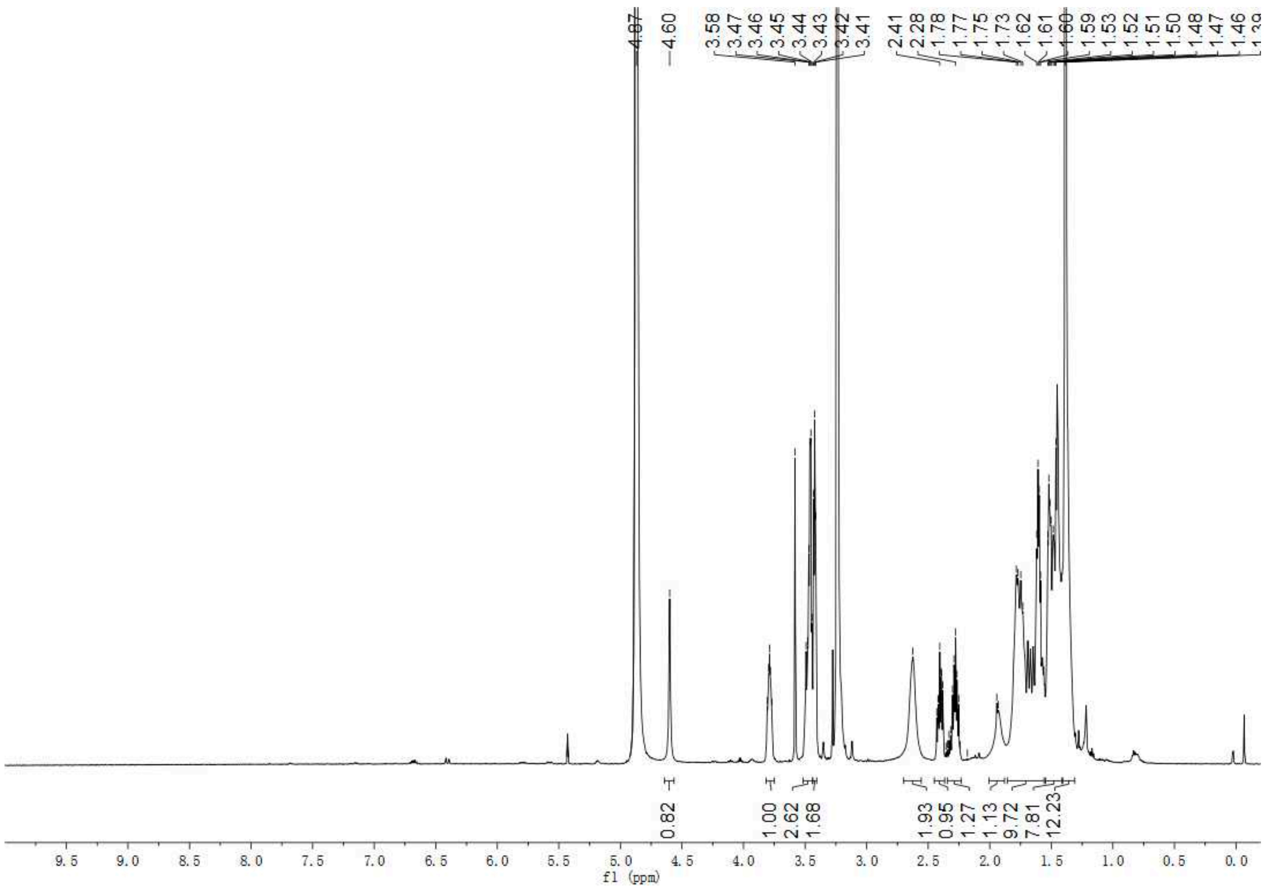

Figure S10. ^{1}H NMR spectrum (600 MHz, CD_3OD) of **1a**

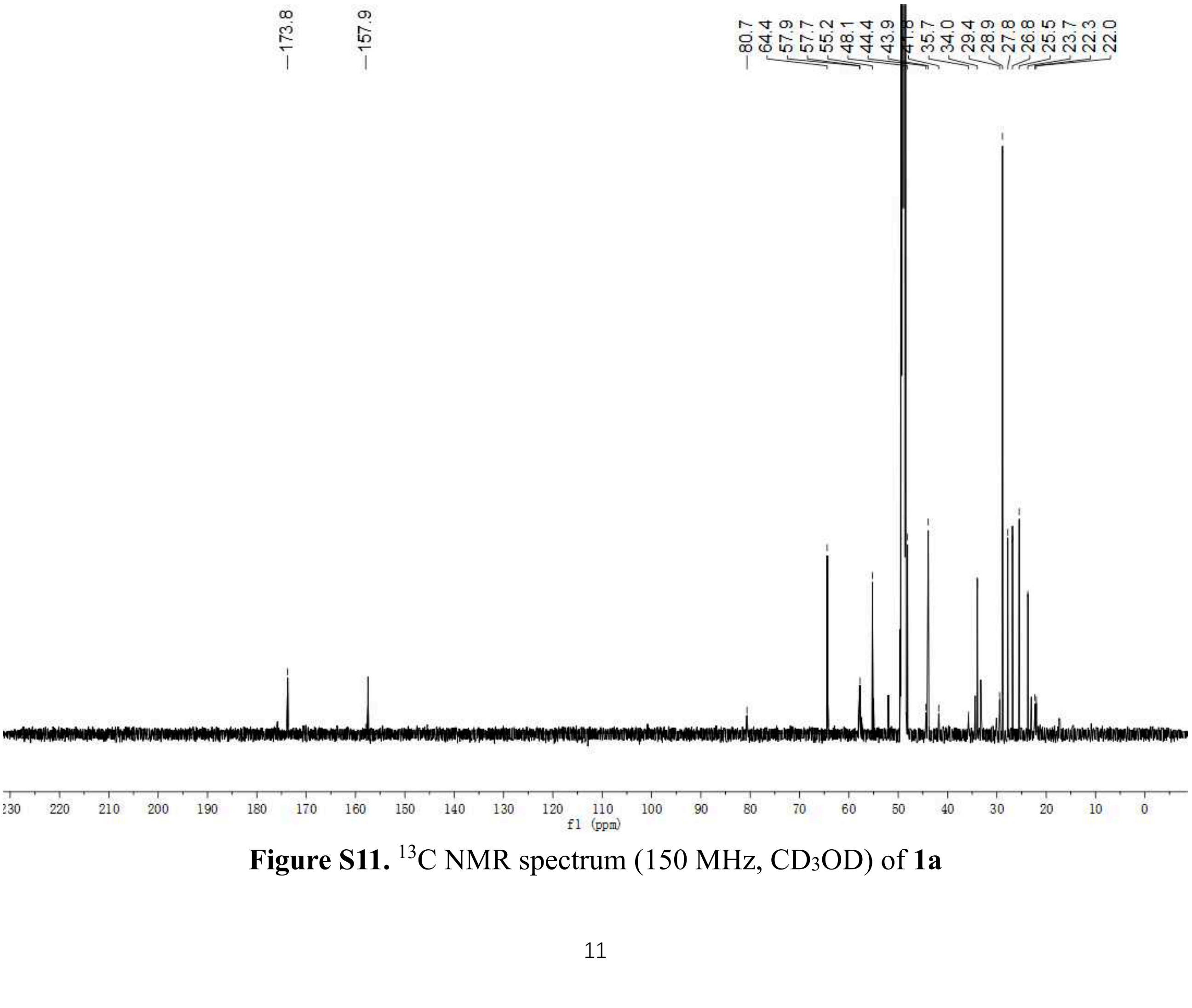

Figure S11. ^{13}C NMR spectrum (150 MHz, CD_3OD) of **1a**

Qualitative Analysis Report

**Data Filename**	D2.d	**Sample Name**	D2
**Sample Type**	Sample	**Position**	P1-F7
**Instrument Name**	Instrument 1	**User Name**	
**Acq Method**	20240114-HRMS-Pos-1.5min-5%H2O.m	**Acquired Time**	2024/4/14 12:37:14
**IRM Calibration Status**	Success	**DA Method**	Default.m
**Comment**			

**Sample Group**		**Info.**	
**Stream Name**	LC 1	**Acquisition SW Version**	6200 series TOF/6500 series Q-TOF B.06.01 (B6172 SP1)

User Spectra

Fragmentor Voltage	Collision Energy	Ionization Mode
175	0	ESI

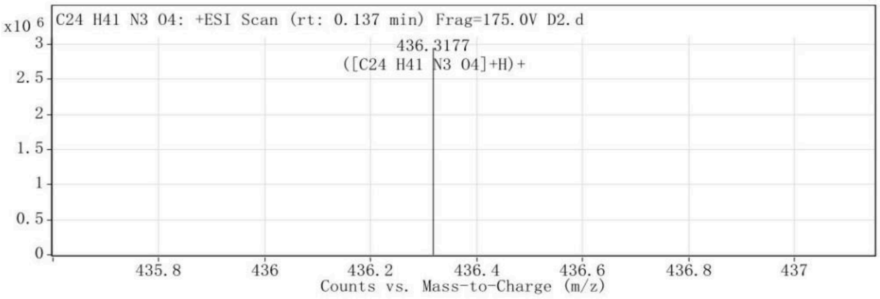

Peak List

m/z	z	Abund	Formula	Ion
84.9609		93328.29		
336.2659	1	345783.22		
337.2687	1	69103.84		
434.3087	1	171515.16		
435.3108	1	46315.07		
436.3177	1	2949663.5	C24 H41 N3 O4	(M+H)+
437.3229	1	708395	C24 H41 N3 O4	(M+H)+
438.3235	1	132290.03	C24 H41 N3 O4	(M+H)+
448.2815	1	57578.01		
893.6088		47426.85		

Formula Calculator Element Limits

Element	Min	Max
C	3	60
H	0	120
O	0	30
N	0	30
S	0	5
Cl	0	3

Formula Calculator Results

Formula	Best	Mass	Tgt Mass	Diff (ppm)	Ion Species	Score
C24 H41 N3 O4	TRUE	435.3108	435.3097	-2.43	C24 H42 N3 O4	91.48

--- End Of Report ---

Figure S12. HRESIMS analysis of **1b**

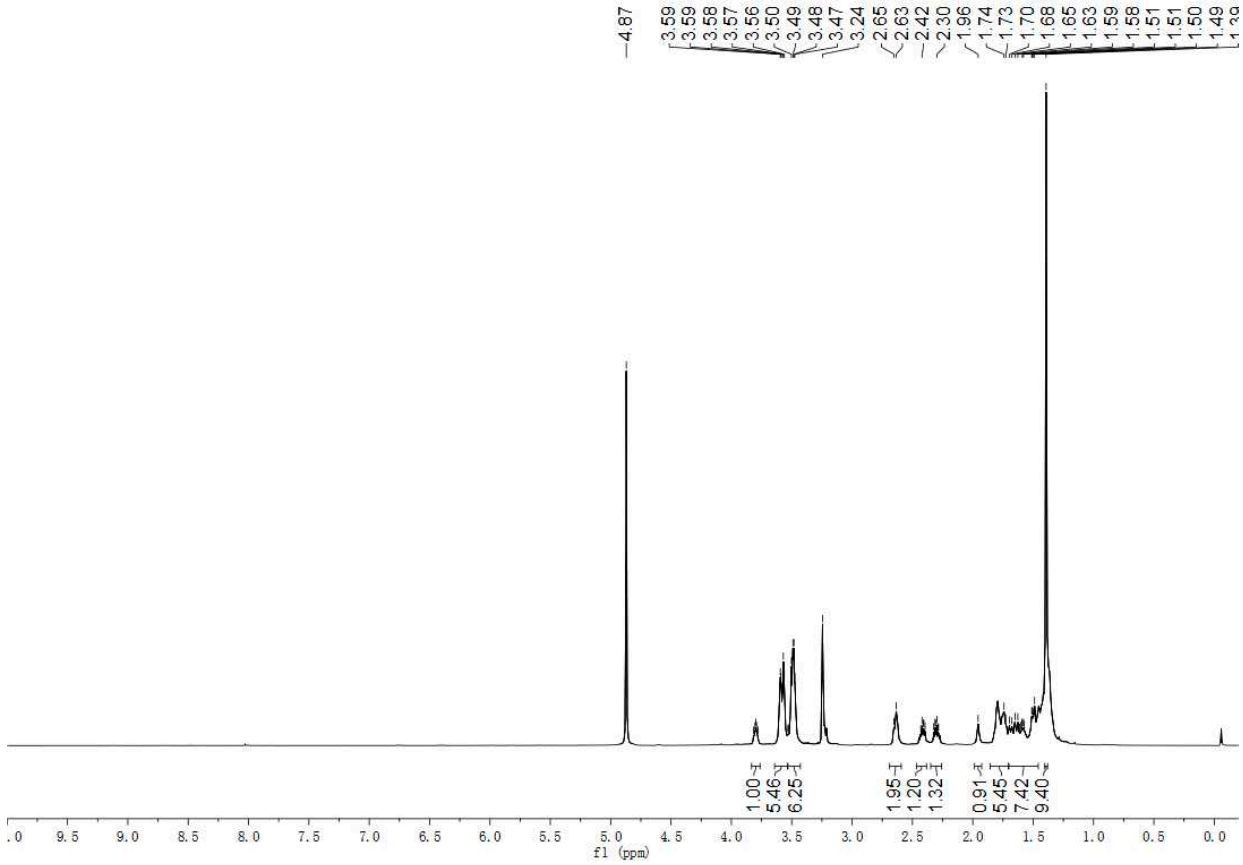

Figure S13. ^{1}H NMR spectrum (600 MHz, CD_3OD) of **1b**

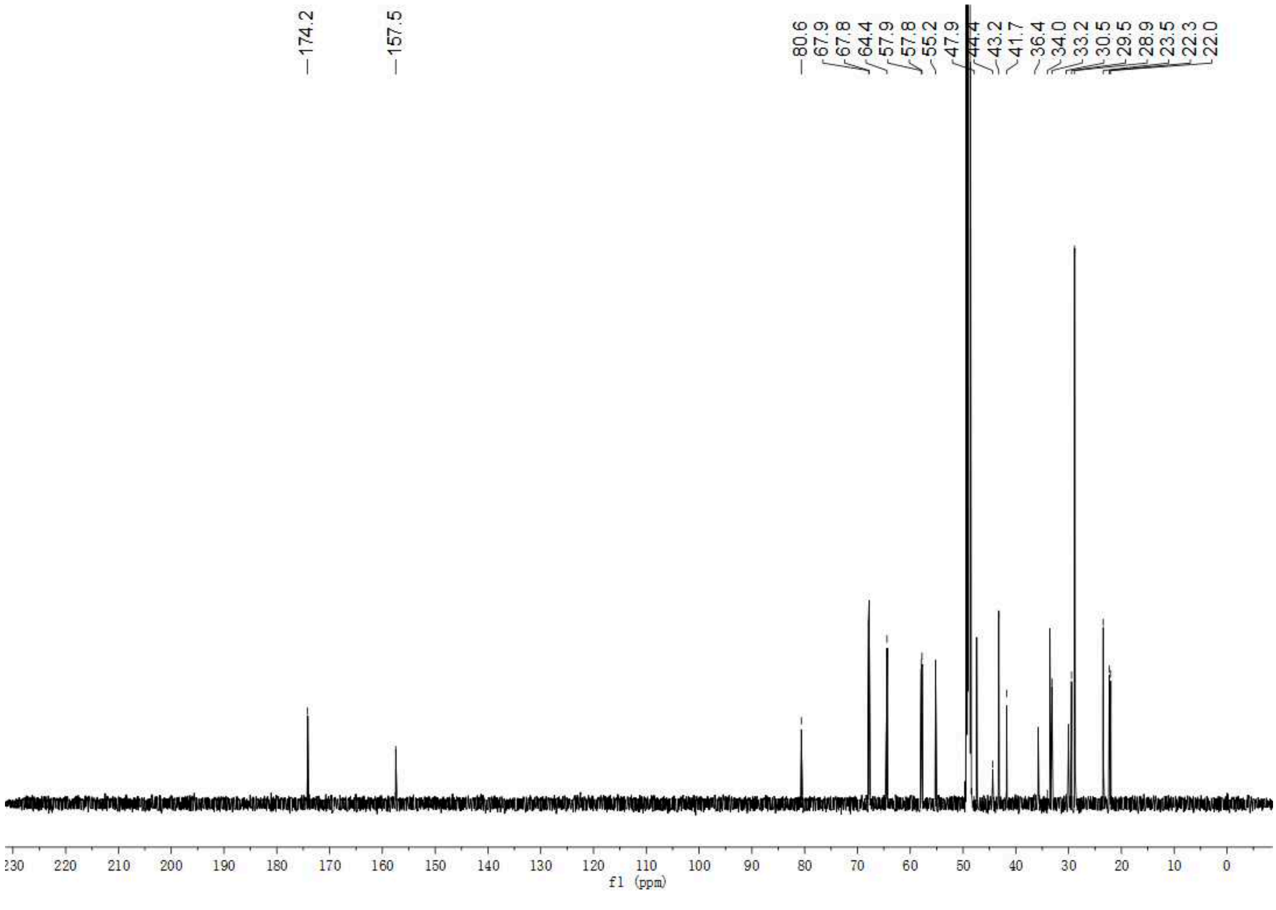

Figure S14. ^{13}C NMR spectrum (150 MHz, CD_3OD) of **1b**

Qualitative Analysis Report

**Data Filename**	D3.d	**Sample Name**	D3
**Sample Type**	Sample	**Position**	P1-F8
**Instrument Name**	Instrument 1	**User Name**	
**Acq Method**	20240114-HRMS-Pos-1.5min-5%H2O.m	**Acquired Time**	2024/4/14 12:39:30
**IRM Calibration Status**	Success	**DA Method**	Default.m
**Comment**			

**Sample Group**		**Info.**	
**Stream Name**	LC 1	**Acquisition SW Version**	6200 series TOF/6500 series Q-TOF B.06.01 (B6172 SP1)

User Spectra

Fragmentor Voltage	Collision Energy	Ionization Mode
175	0	ESI

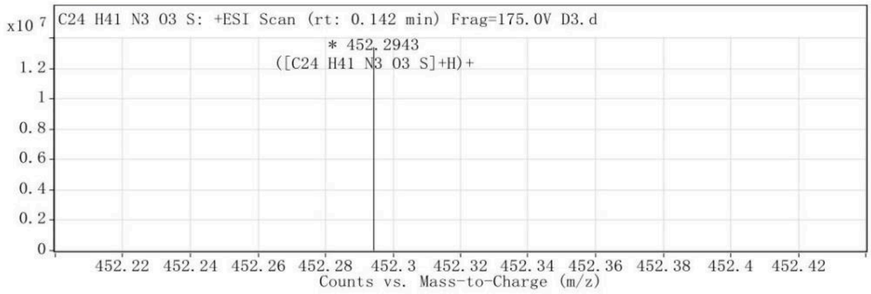

Peak List

m/z	z	Abund	Formula	Ion
352.2421	1	1302300		
446.2481	1	468816.19		
450.2787	1	1934875		
451.2826	1	597494.38		
452.2943	1	13407070	C24 H41 N3 O3 S	(M+H)+
452.4709	1	720183.31		
453.2973	1	3831696.5	C24 H41 N3 O3 S	(M+H)+
454.2971	1	1121885.5	C24 H41 N3 O3 S	(M+H)+
925.5629		779237.88		
926.566	1	469565.81		

Formula Calculator Element Limits

Element	Min	Max
C	3	60
H	0	120
O	0	30
N	0	30
S	0	5
Cl	0	3

Formula Calculator Results

Formula	Best	Mass	Tgt Mass	Diff (ppm)	Ion Species	Score
C24 H41 N3 O3 S	TRUE	451.2872	451.2869	-0.64	C24 H42 N3 O3 S	97.87

--- End Of Report ---

Figure S15. HRESIMS analysis of **1c**

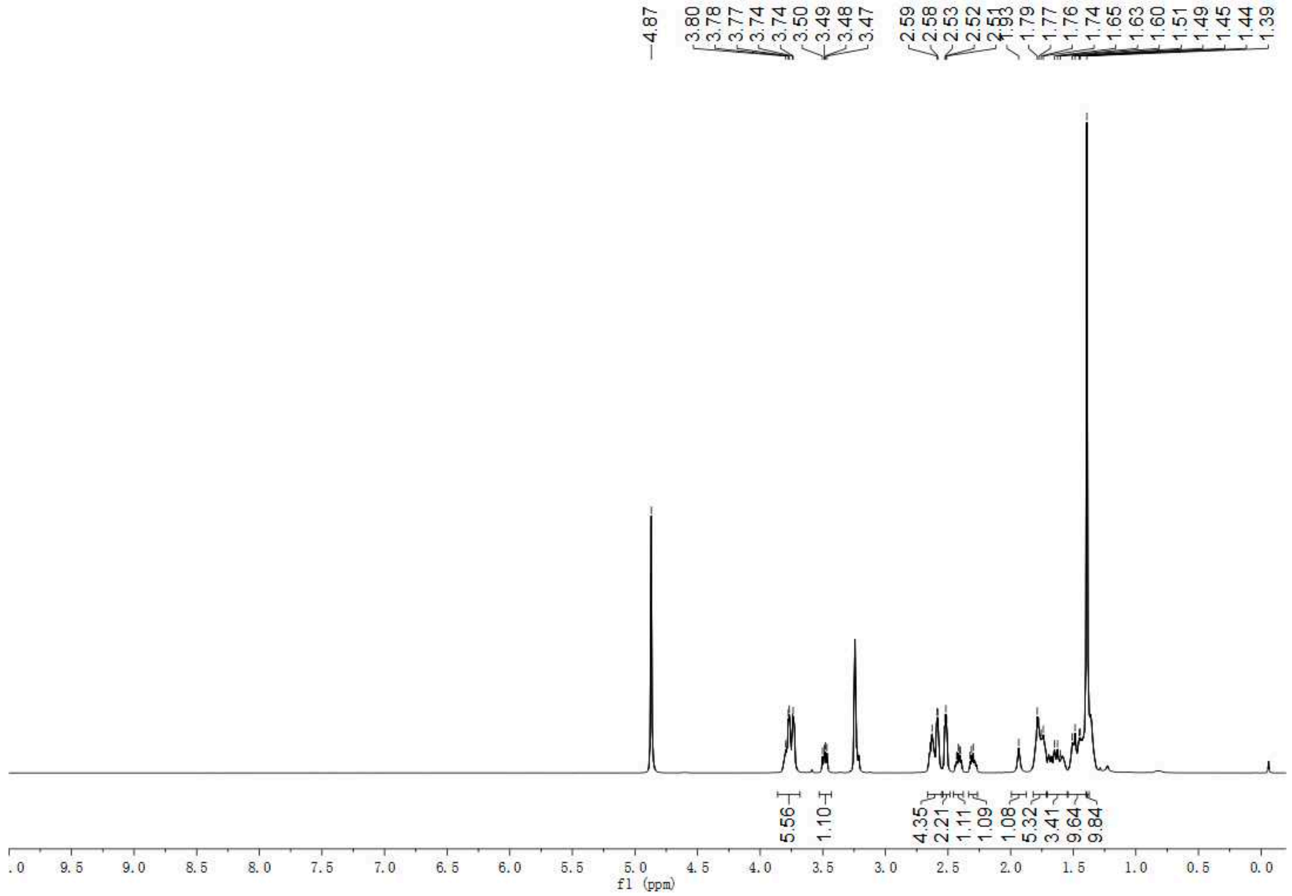

Figure S16. ^{1}H NMR spectrum (600 MHz, CD_3OD) of **1c**

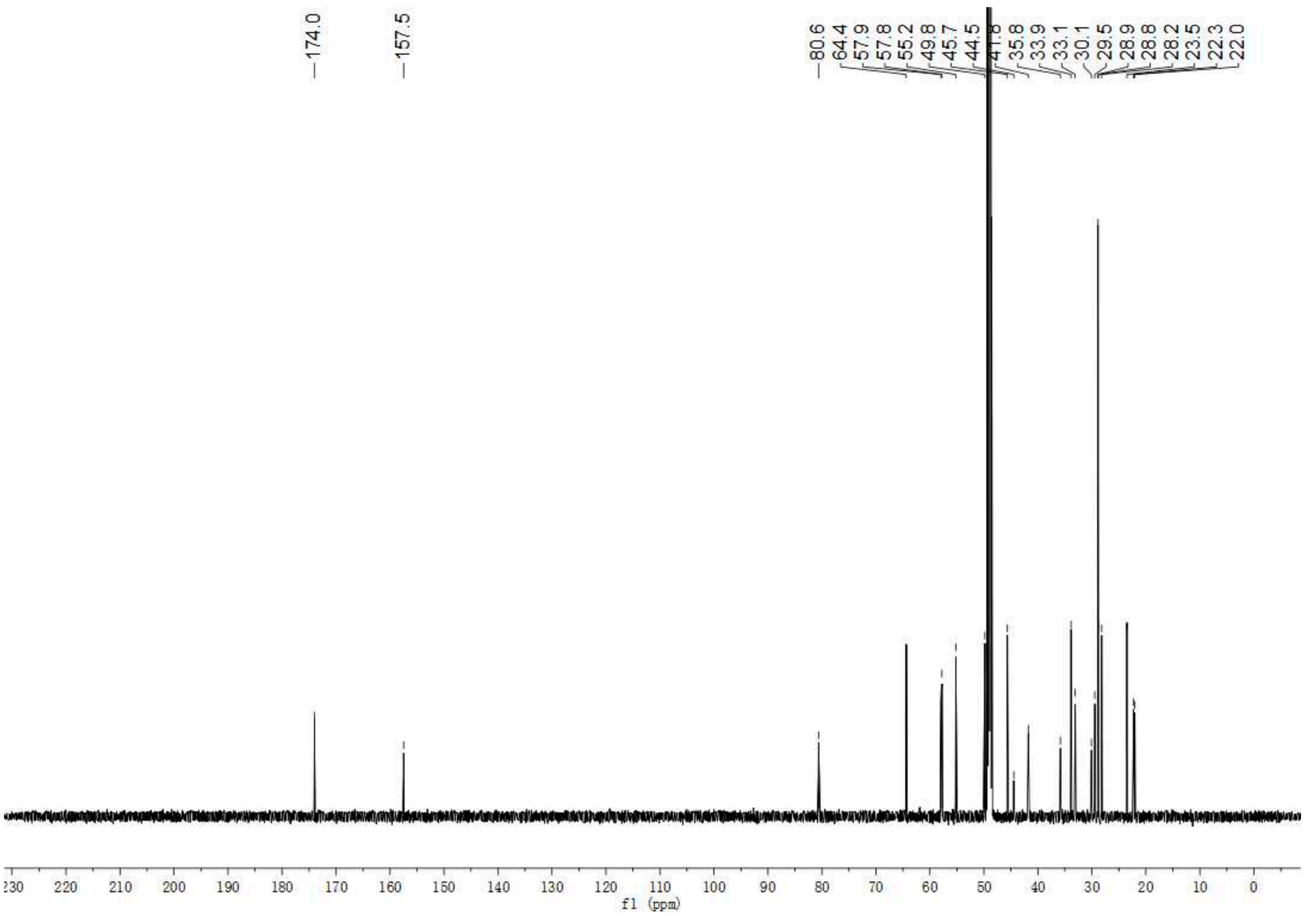

Figure S17. ^{13}C NMR spectrum (150 MHz, CD_3OD) of **1c**

Qualitative Analysis Report

**Data Filename**	D4.d	**Sample Name**	D4
**Sample Type**	Sample	**Position**	P1-F9
**Instrument Name**	Instrument 1	**User Name**	
**Acq Method**	20240114-HRMS-Pos-1.5min-5%H2O.m	**Acquired Time**	2024/4/14 12:41:47
**IRM Calibration Status**	Success	**DA Method**	Default.m
**Comment**			

Sample Group | **Info.**
Stream Name LC 1 | **Acquisition SW Version** 6200 series TOF/6500 series Q-TOF B.06.01 (B6172 SP1)

User Spectra

Fragmentor Voltage	Collision Energy	Ionization Mode
175	0	ESI

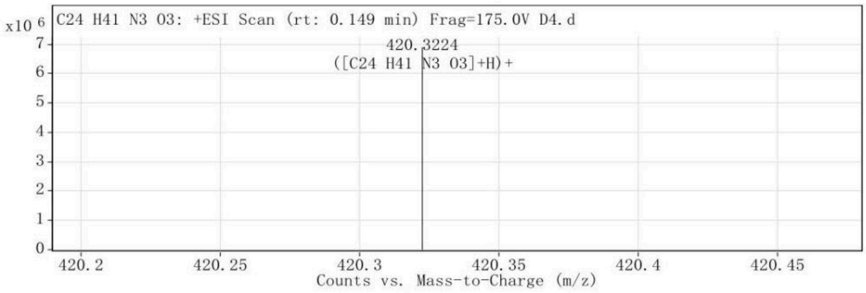

Peak List

m/z	z	Abund	Formula	Ion
320.2716	1	911979.88		
414.2763	1	465533.56		
416.2914	1	313158		
418.3075	1	593774.88		
420.3224	1	6899851.5	C24 H41 N3 O3	(M+H)+
420.4938	1	345784.03		
421.3259	1	1833816	C24 H41 N3 O3	(M+H)+
432.2866	1	339202.78		
436.3179	1	354009.38		
861.6193		496235.22		

Formula Calculator Element Limits

Element	Min	Max
C	3	60
H	0	120
O	0	30
N	0	30
S	0	5
Cl	0	3

Formula Calculator Results

Formula	Best	Mass	Tgt Mass	Diff (ppm)	Ion Species	Score
C24 H41 N3 O3	TRUE	419.3153	419.3148	-1.1	C24 H42 N3 O3	99.1

--- End Of Report ---

Figure S18. HRESIMS analysis of **1d**

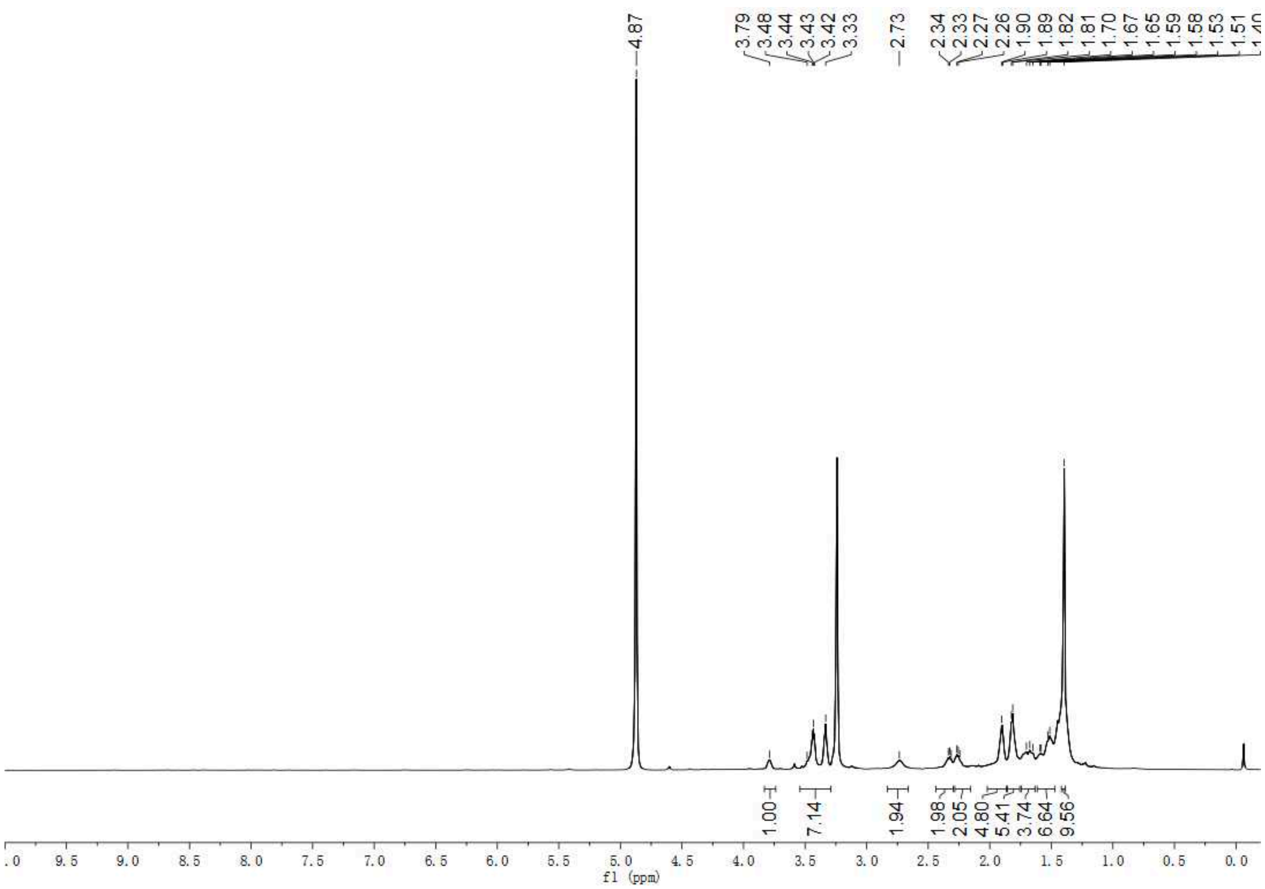

Figure S19. ^{1}H NMR spectrum (600 MHz, CD_3OD) of **1d**

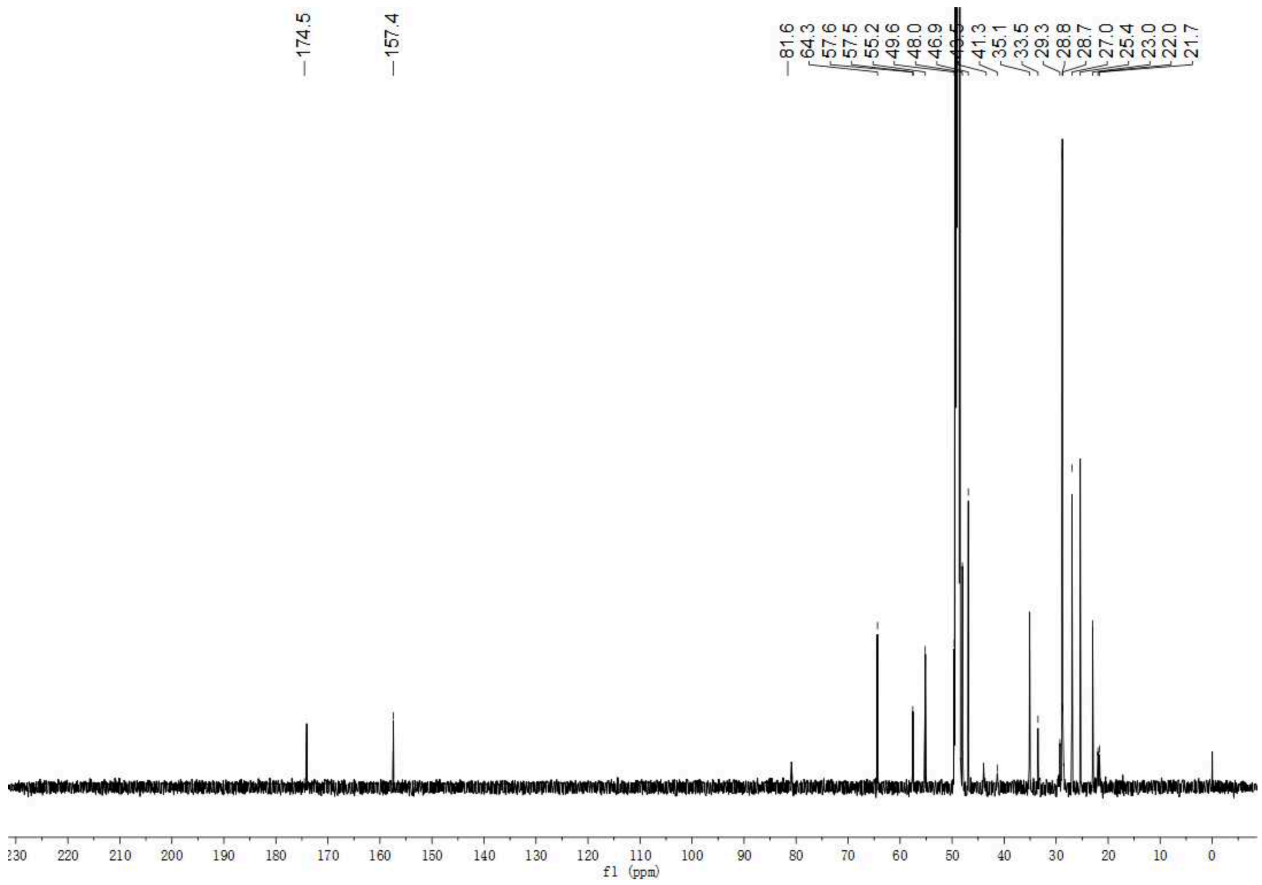

Figure S20. ^{13}C NMR spectrum (150 MHz, CD_3OD) of **1d**

Qualitative Analysis Report

**Data Filename**	D5.d	**Sample Name**	D5
**Sample Type**	Sample	**Position**	P1-A1
**Instrument Name**	Instrument 1	**User Name**	
**Acq Method**	20200905-HRMS(+).m	**Acquired Time**	2024/4/14 12:53:39
**IRM Calibration Status**	Success	**DA Method**	Default.m
**Comment**			

**Sample Group**		**Info.**	
**Stream Name**	LC 1	**Acquisition SW Version**	6200 series TOF/6500 series Q-TOF B.06.01 (B6172 SP1)

User Spectra

Fragmentor Voltage	Collision Energy	Ionization Mode
175	0	ESI

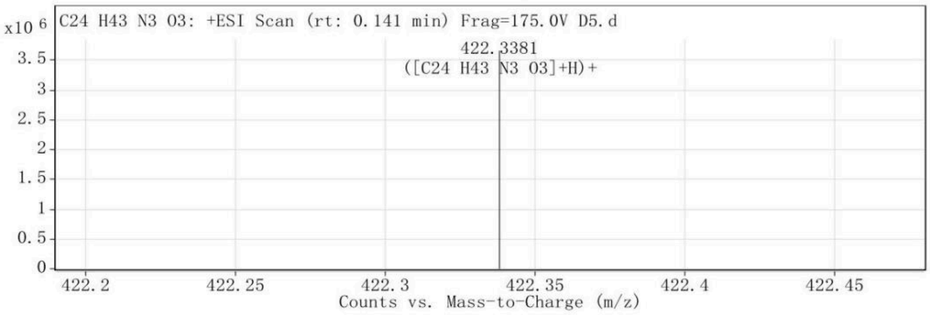

Peak List

m/z	z	Abund	Formula	Ion
322.2861	1	463256.03		
422.3381	1	3660375.25	C24 H43 N3 O3	(M+H)+
422.5093	2	190375.77		
423.3421	1	941738.25	C24 H43 N3 O3	(M+H)+
424.3444	1	152575.59	C24 H43 N3 O3	(M+H)+
444.3207	1	231764.02		
460.2946	1	168184.75		
865.65		943006.25		
866.6538	1	598463.13		
867.6561	1	161464.36		

Formula Calculator Element Limits

Element	Min	Max
C	3	60
H	0	120
O	0	30
N	0	30
S	0	5
Cl	0	3

Formula Calculator Results

Formula	Best	Mass	Tgt Mass	Diff (ppm)	Ion Species	Score
C24 H43 N3 O3	TRUE	421.331	421.3304	-1.3	C24 H44 N3 O3	97.8

--- End Of Report ---

Agilent Technologies Page 1 of 1 Printed at: 21:57 on: 2024/11/17

Figure S21. HRESIMS analysis of **1e**

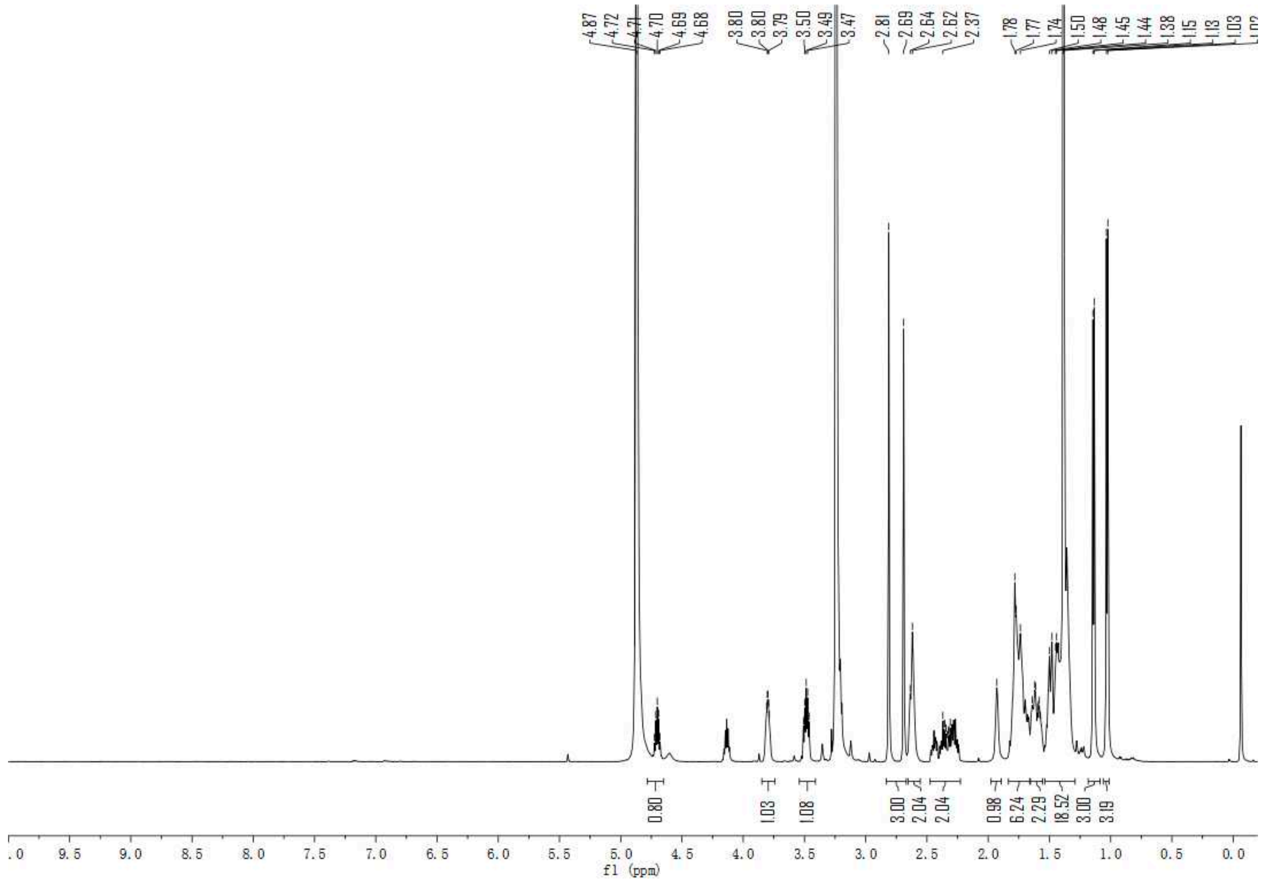

Figure S22. ^{1}H NMR spectrum (600 MHz, CD_3OD) of **1e**

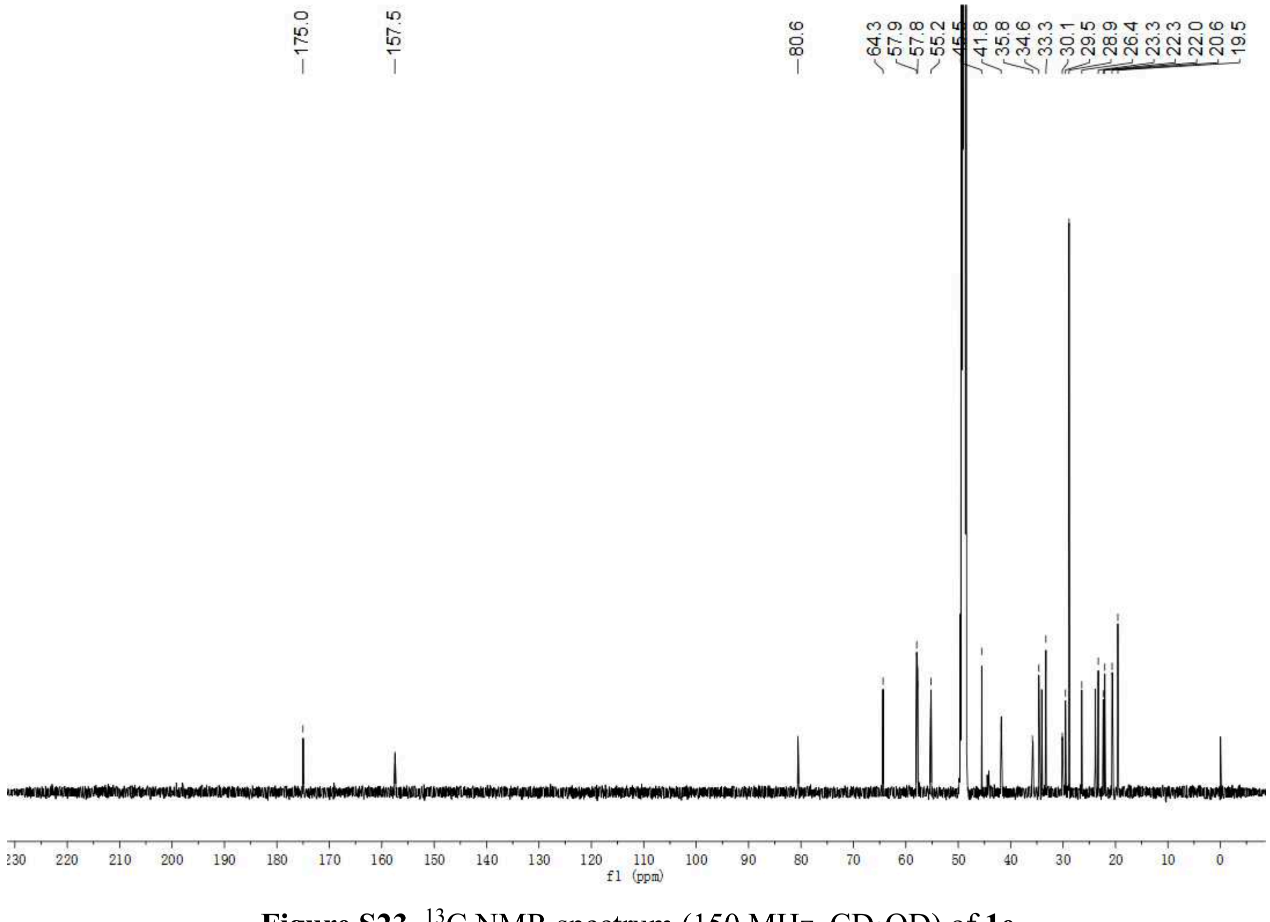

Figure S23. ^{13}C NMR spectrum (150 MHz, CD_3OD) of **1e**

Qualitative Analysis Report

**Data Filename**	D6.d	**Sample Name**	D6
**Sample Type**	Sample	**Position**	P1-A2
**Instrument Name**	Instrument 1	**User Name**	
**Acq Method**	20200905-HRMS(+).m	**Acquired Time**	2024/4/14 12:56:25
**IRM Calibration Status**	Success	**DA Method**	Default.m
**Comment**			

**Sample Group**		**Info.**	
**Stream Name**	LC 1	**Acquisition SW Version**	6200 series TOF/6500 series Q-TOF B.06.01 (B6172 SP1)

User Spectra

Fragmentor Voltage	Collision Energy	Ionization Mode
175	0	ESI

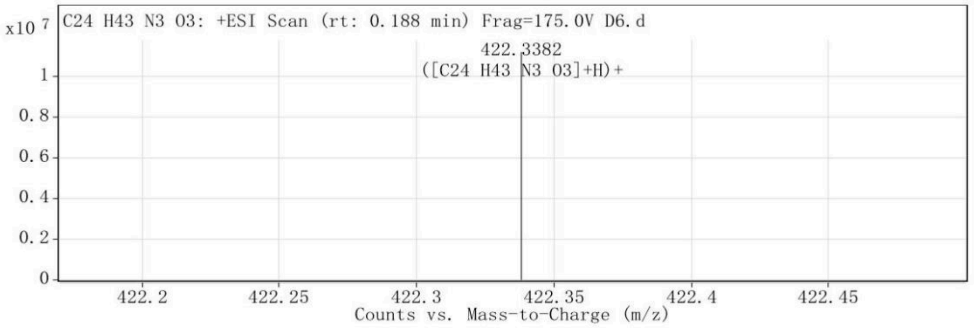

Peak List

m/z	z	Abund	Formula	Ion
322.2862	1	1532066.5		
323.2892	1	351603.84		
381.2758	1	422115.75		
420.3229	1	252947.47		
422.3382	1	11180599	C24 H43 N3 O3	(M+H)+
422.5106	1	631452.31		
423.3412	1	3117980.25	C24 H43 N3 O3	(M+H)+
424.3451	1	512656.75	C24 H43 N3 O3	(M+H)+
865.6509	1	1422050		
866.6551	1	787567.19		

Formula Calculator Element Limits

Element	Min	Max
C	3	60
H	0	120
O	0	30
N	0	30
S	0	5
Cl	0	3

Formula Calculator Results

Formula	Best	Mass	Tgt Mass	Diff (ppm)	Ion Species	Score
C24 H43 N3 O3	TRUE	421.3309	421.3304	-1.08	C24 H44 N3 O3	99.37

--- End Of Report ---

Figure S24. HRESIMS analysis of **1f**

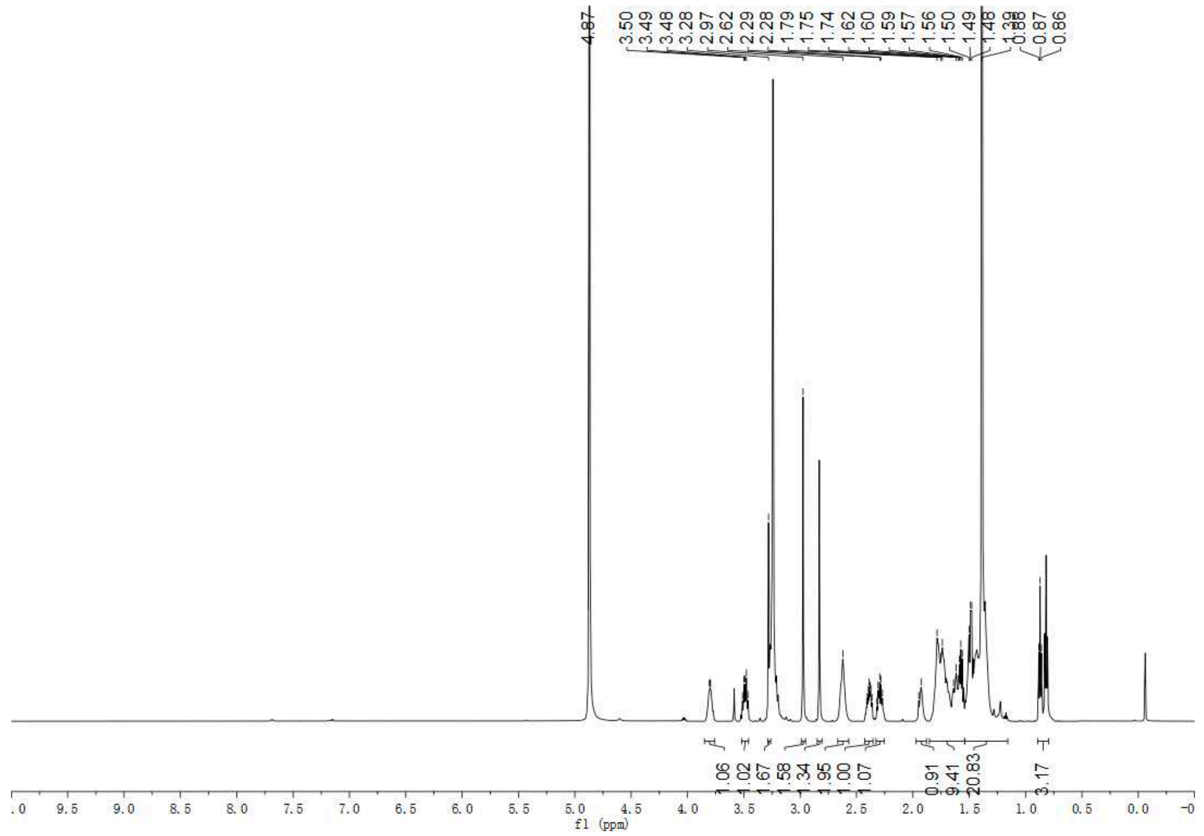

Figure S25. ^{1}H NMR spectrum (600 MHz, CD_3OD) of **1f**

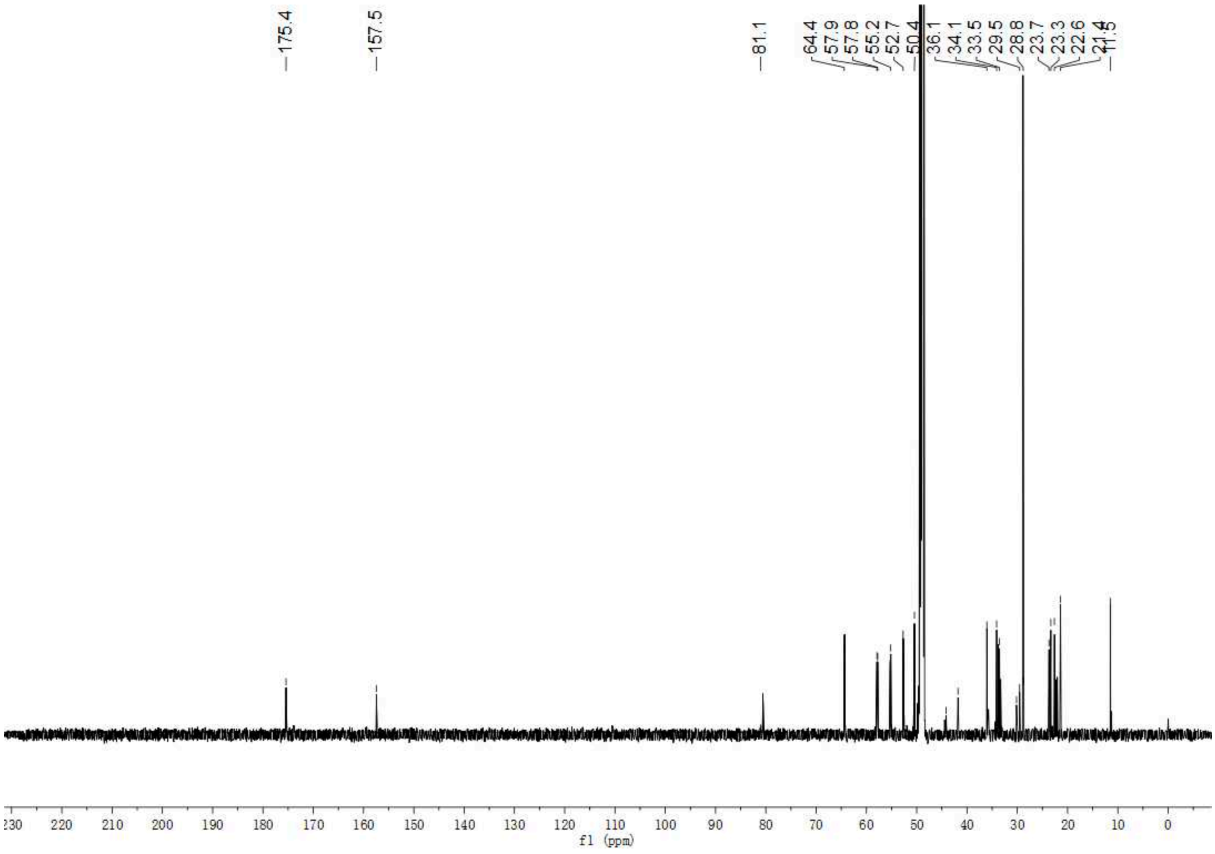

Figure S26. ^{13}C NMR spectrum (150 MHz, CD_3OD) of **1f**

Qualitative Analysis Report

**Data Filename**	D7.d	**Sample Name**	D7
**Sample Type**	Sample	**Position**	P1-A3
**Instrument Name**	Instrument 1	**User Name**	
**Acq Method**	20200905-HRMS(+).m	**Acquired Time**	2024/4/14 12:59:07
**IRM Calibration Status**	Success	**DA Method**	Default.m
**Comment**			

**Sample Group**		**Info.**	
**Stream Name**	LC 1	**Acquisition SW Version**	6200 series TOF/6500 series Q-TOF B.06.01 (B6172 SP1)

User Spectra

Fragmentor Voltage	Collision Energy	Ionization Mode
175	0	ESI

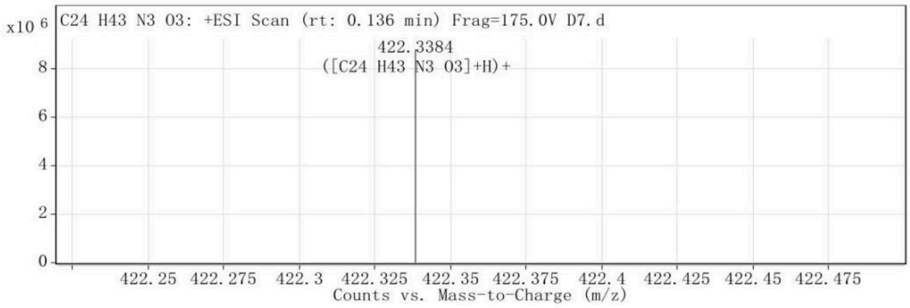

Peak List

m/z	z	Abund	Formula	Ion
322.2865	1	1123572.25		
381.2759	1	393819.03		
420.3235	1	322746.38		
422.3384	1	8803412	C24 H43 N3 O3	(M+H)+
422.511	1	490499.75		
423.3418	1	2413983	C24 H43 N3 O3	(M+H)+
424.3453	1	399624.31	C24 H43 N3 O3	(M+H)+
432.288	1	693290.25		
865.6511	1	659388.25		
866.6541	1	357384		

Formula Calculator Element Limits

Element	Min	Max
C	3	60
H	0	120
O	0	30
N	0	30
S	0	5
Cl	0	3

Formula Calculator Results

Formula	Best	Mass	Tgt Mass	Diff (ppm)	Ion Species	Score
C24 H43 N3 O3	TRUE	421.3312	421.3304	-1.82	C24 H44 N3 O3	98.39

--- End Of Report ---

Agilent Technologies Page 1 of 1 Printed at: 22:00 on: 2024/11/17

Figure S27. HRESIMS analysis of **1g**

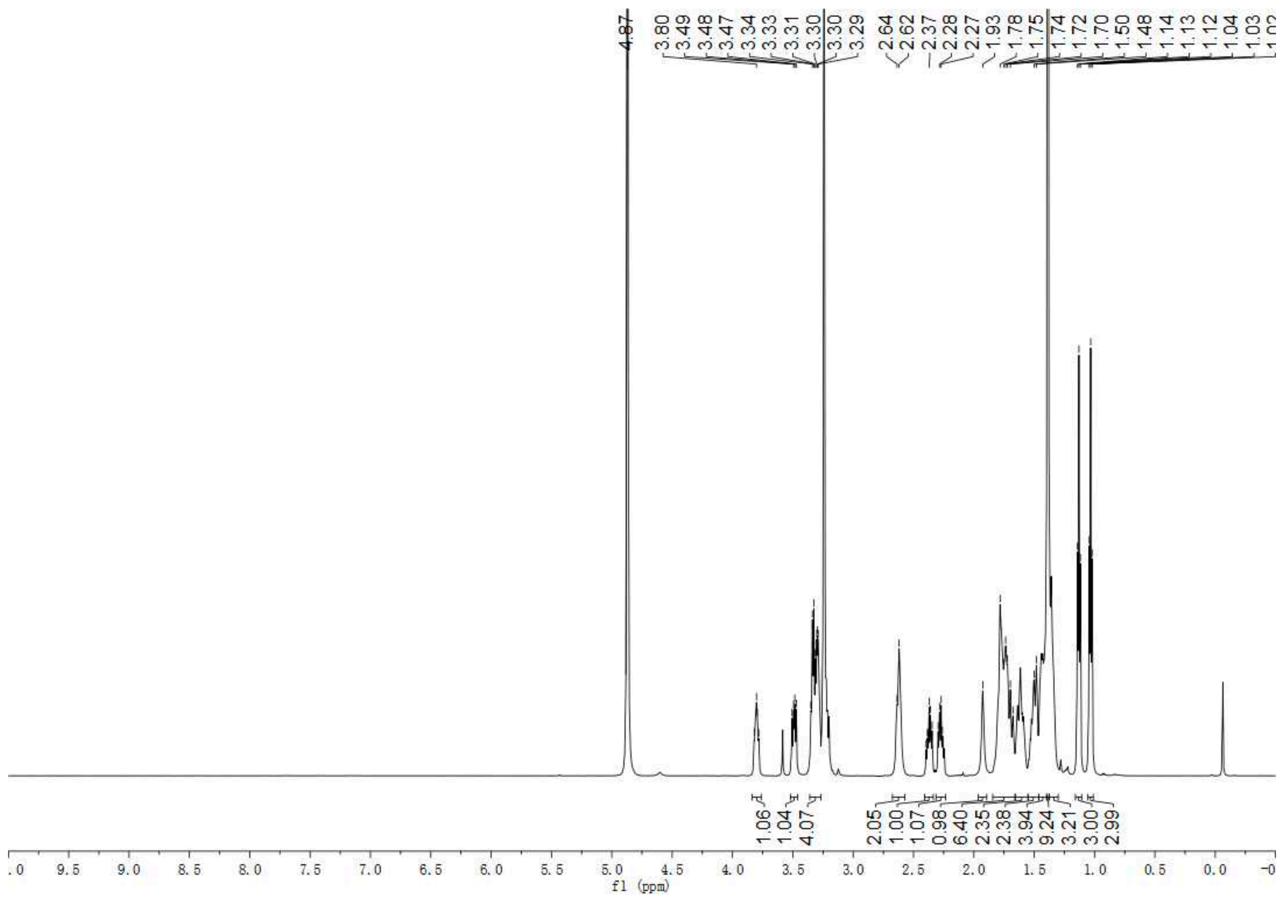

Figure S28. ^{1}H NMR spectrum (600 MHz, CD_3OD) of **1g**

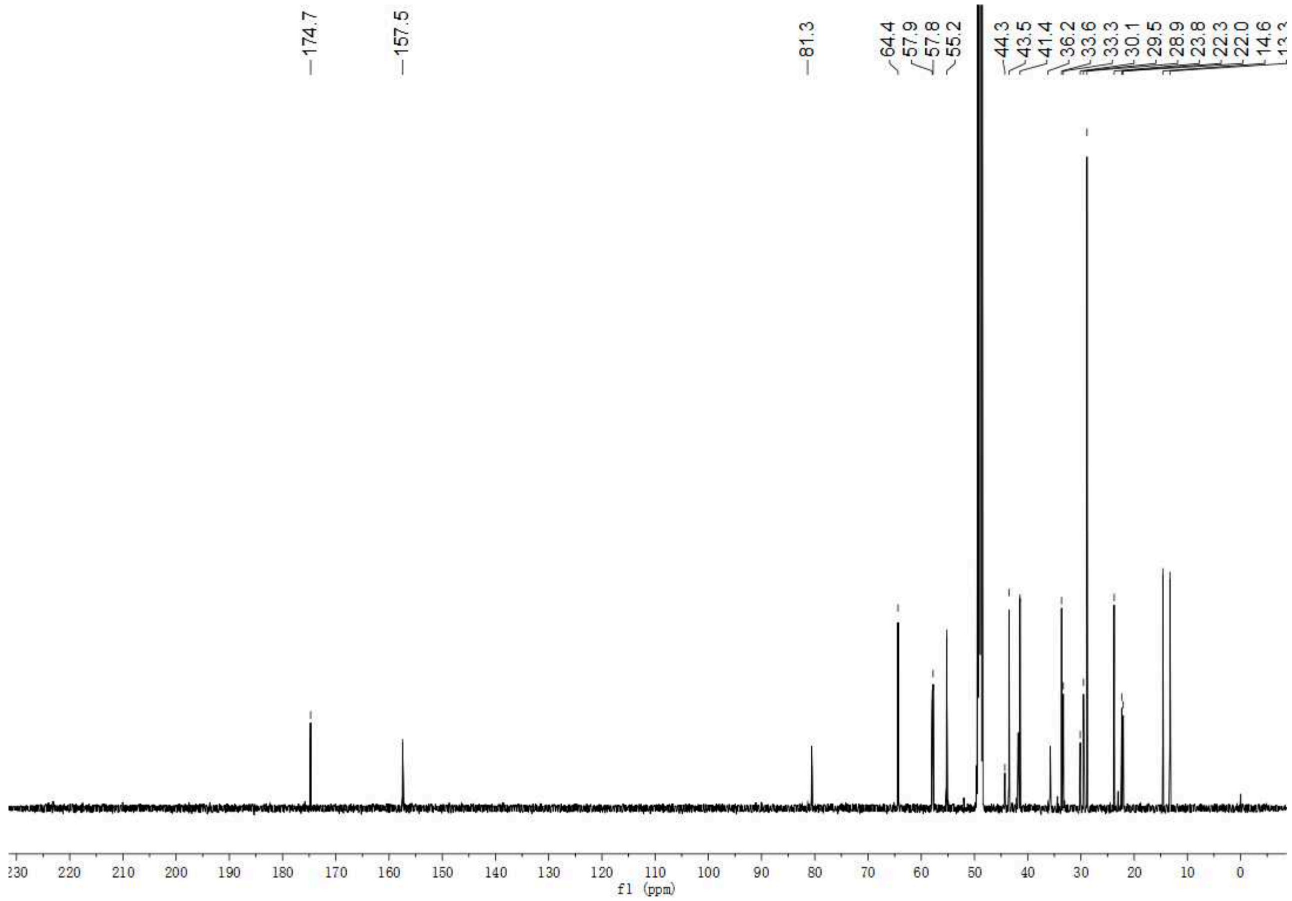

Figure S29. ^{13}C NMR spectrum (150 MHz, CD_3OD) of **1g**

Qualitative Analysis Report

**Data Filename**	E1.d	**Sample Name**	E1
**Sample Type**	Sample	**Position**	P1-B6
**Instrument Name**	Instrument 1	**User Name**	
**Acq Method**	20200905-HRMS(+).m	**Acquired Time**	2024/4/14 13:31:43
**IRM Calibration Status**	Success	**DA Method**	Default.m
**Comment**			

**Sample Group**		**Info.**	
**Stream Name**	LC 1	**Acquisition SW Version**	6200 series TOF/6500 series Q-TOF B.06.01 (B6172 SP1)

User Spectra

Fragmentor Voltage	Collision Energy	Ionization Mode
175	0	ESI

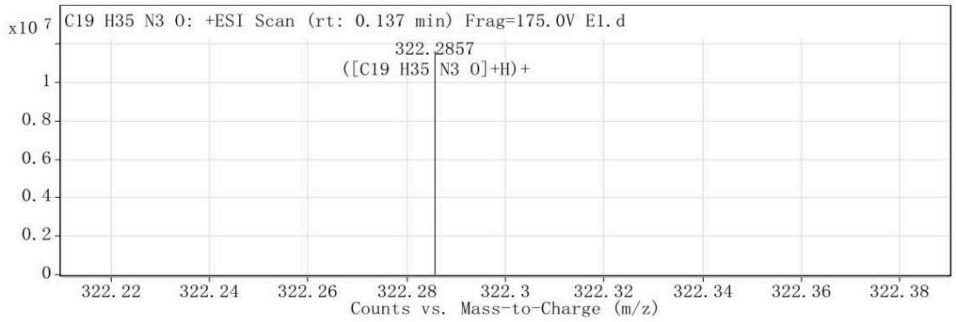

Peak List

m/z	z	Abund	Formula	Ion
162.974	1	198796.14		
249.1967	1	179406.09		
320.2706	1	351536.25		
322.2857	1	11622786	C19 H35 N3 O	(M+H)+
322.4371	1	647972.44		
323.2888	1	2547176.5	C19 H35 N3 O	(M+H)+
323.44	1	150373.06		
324.2924	1	292018.47	C19 H35 N3 O	(M+H)+
643.5645	1	159766.09		
679.5413	1	215943.02		

Formula Calculator Element Limits

Element	Min	Max
C	3	60
H	0	120
O	0	30
N	0	30
S	0	5
Cl	0	3

Formula Calculator Results

Formula	Best	Mass	Tgt Mass	Diff (ppm)	Ion Species	Score
C19 H35 N3 O	TRUE	321.2785	321.278	-1.4	C19 H36 N3 O	99.31

--- End Of Report ---

Figure S30. HRESIMS analysis of **1h**

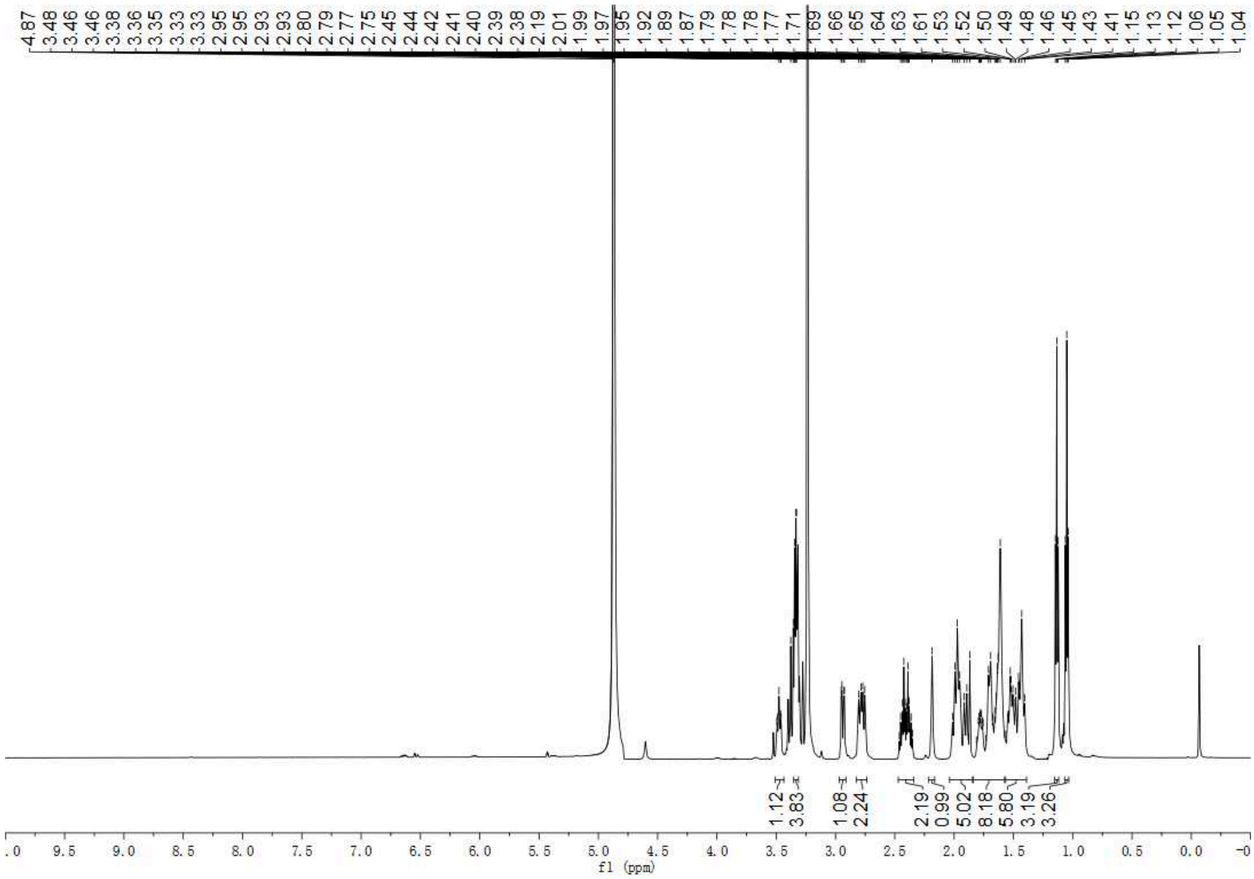

Figure S31. ^{1}H NMR spectrum (600 MHz, CD_3OD) of **1h**

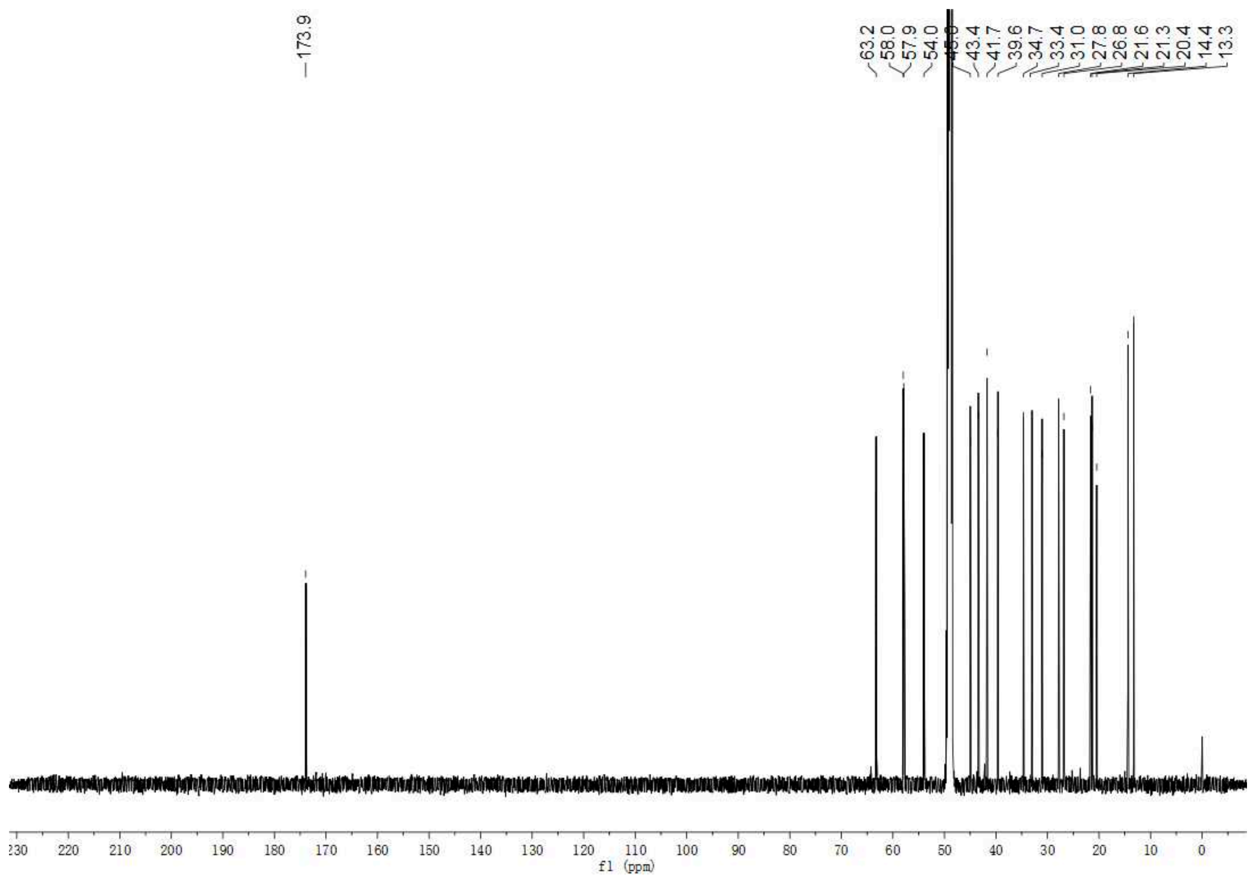

Figure S32. ^{13}C NMR spectrum (150 MHz, CD_3OD) of **1h**

Qualitative Analysis Report

**Data Filename**	B1.d	**Sample Name**	B1
**Sample Type**	Sample	**Position**	P1-E8
**Instrument Name**	Instrument 1	**User Name**	
**Acq Method**	20240114-HRMS-Pos-1.5min-5%H2O.m	**Acquired Time**	2024/4/14 11:56:38
**IRM Calibration Status**	Success	**DA Method**	Default.m
**Comment**			

**Sample Group**		**Info.**	
**Stream Name**	LC 1	**Acquisition SW Version**	6200 series TOF/6500 series Q-TOF B.06.01 (B6172 SP1)

User Spectra

Fragmentor Voltage	Collision Energy	Ionization Mode
175	0	ESI

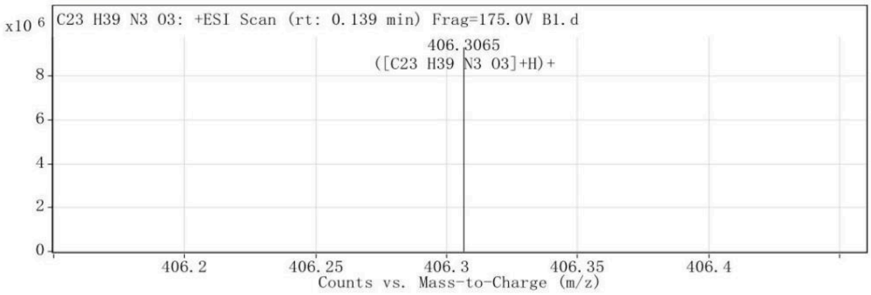

Peak List

m/z	z	Abund	Formula	Ion
98.0968		994590.13		
203.6579	2	818773.63		
281.2229	1	728318		
404.2915	1	384792.78		
406.3065	1	9301593	C23 H39 N3 O3	(M+H)+
406.4739	1	521736.34		
407.3107	1	3594008	C23 H39 N3 O3	(M+H)+
408.3152	1	751408.81	C23 H39 N3 O3	(M+H)+
833.5884		567516.81		
834.5923	1	434647.59		

Formula Calculator Element Limits

Element	Min	Max
C	3	60
H	0	120
O	0	30
N	0	30
S	0	5
Cl	0	3

Formula Calculator Results

Formula	Best	Mass	Tgt Mass	Diff (ppm)	Ion Species	Score
C23 H39 N3 O3	TRUE	405.2997	405.2991	-1.31	C23 H40 N3 O3	81

--- End Of Report ---

Figure S33. HRESIMS analysis of **2a**

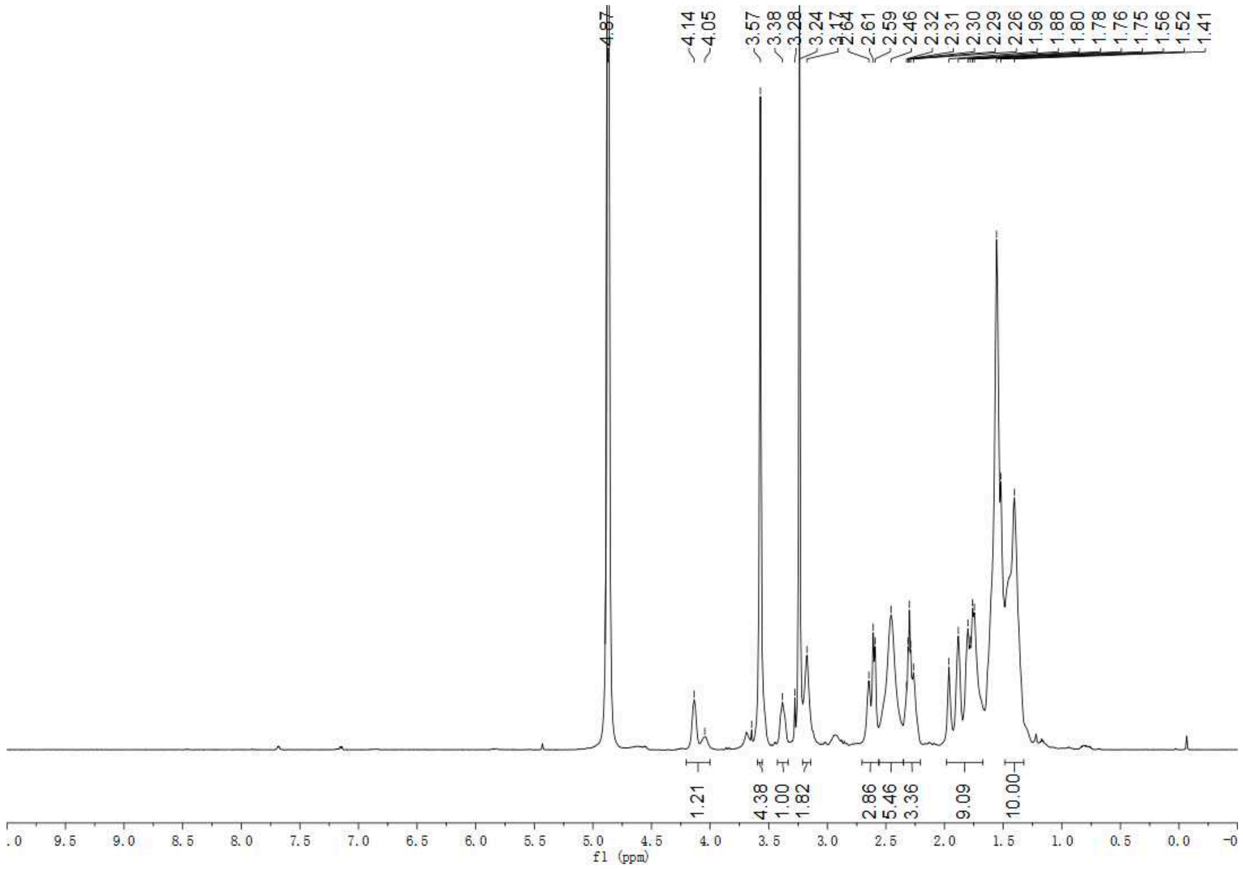

Figure S34. ^{1}H NMR spectrum (600 MHz, CD_3OD) of **2a**

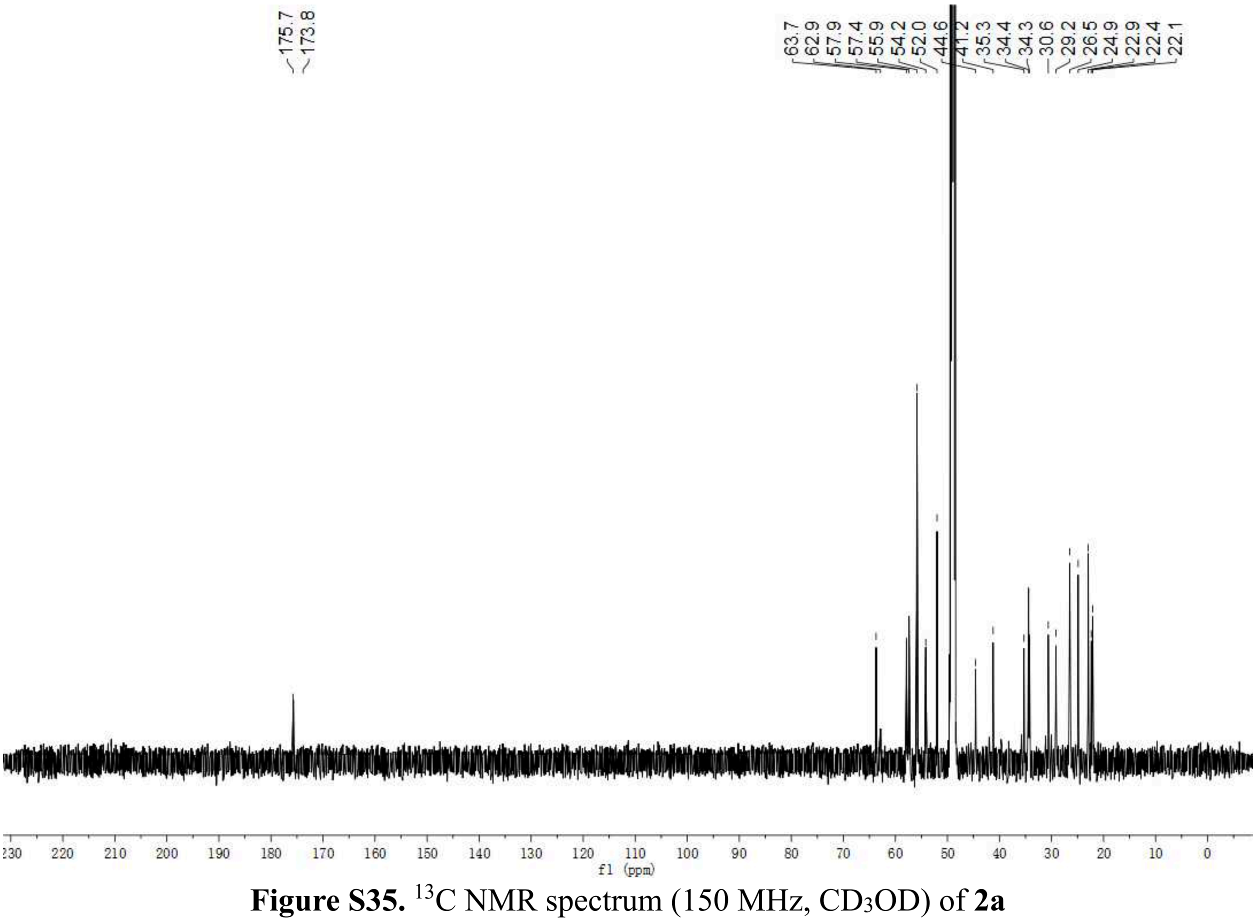

Figure S35. ^{13}C NMR spectrum (150 MHz, CD_3OD) of **2a**

Qualitative Analysis Report

**Data Filename**	B2.d	**Sample Name**	B2
**Sample Type**	Sample	**Position**	P1-E9
**Instrument Name**	Instrument 1	**User Name**	
**Acq Method**	20240114-HRMS-Pos-1.5min-5%H2O.m	**Acquired Time**	2024/4/14 11:58:54
**IRM Calibration Status**	Success	**DA Method**	Default.m
**Comment**			

**Sample Group**		**Info.**	
**Stream Name**	LC 1	**Acquisition SW Version**	6200 series TOF/6500 series Q-TOF B.06.01 (B6172 SP1)

User Spectra

Fragmentor Voltage	Collision Energy	Ionization Mode
175	0	ESI

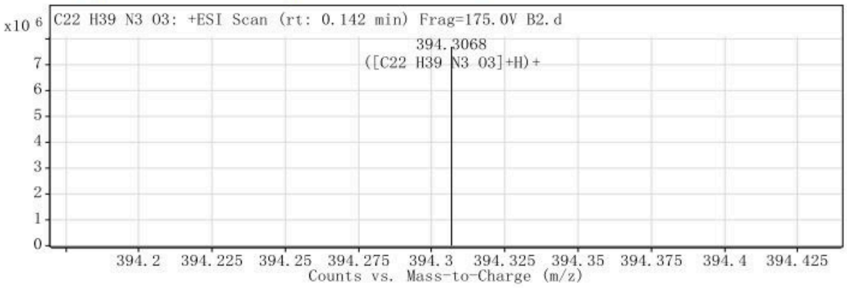

Peak List

m/z	z	Abund	Formula	Ion
86.0974		505430.03		
87.1034		318265.31		
249.1966	1	316652.63		
281.2232	1	685423.5		
394.3068		7683962	C22 H39 N3 O3	(M+H)+
394.4732		392801.66		
395.312	1	5834191.5		
395.4773	1	346457.44		
396.3169	1	1762618.63		
397.324	1	649107.44		

Formula Calculator Element Limits

Element	Min	Max
C	3	60
H	0	120
O	0	30
N	0	30
S	0	5
Cl	0	3

Formula Calculator Results

Formula	Best	Mass	Tgt Mass	Diff (ppm)	Ion Species	Score
C22 H39 N3 O3	TRUE	393.2995	393.2991	-0.92	C22 H40 N3 O3	47.27

--- End Of Report ---

Figure S36. HRESIMS analysis of **2b**

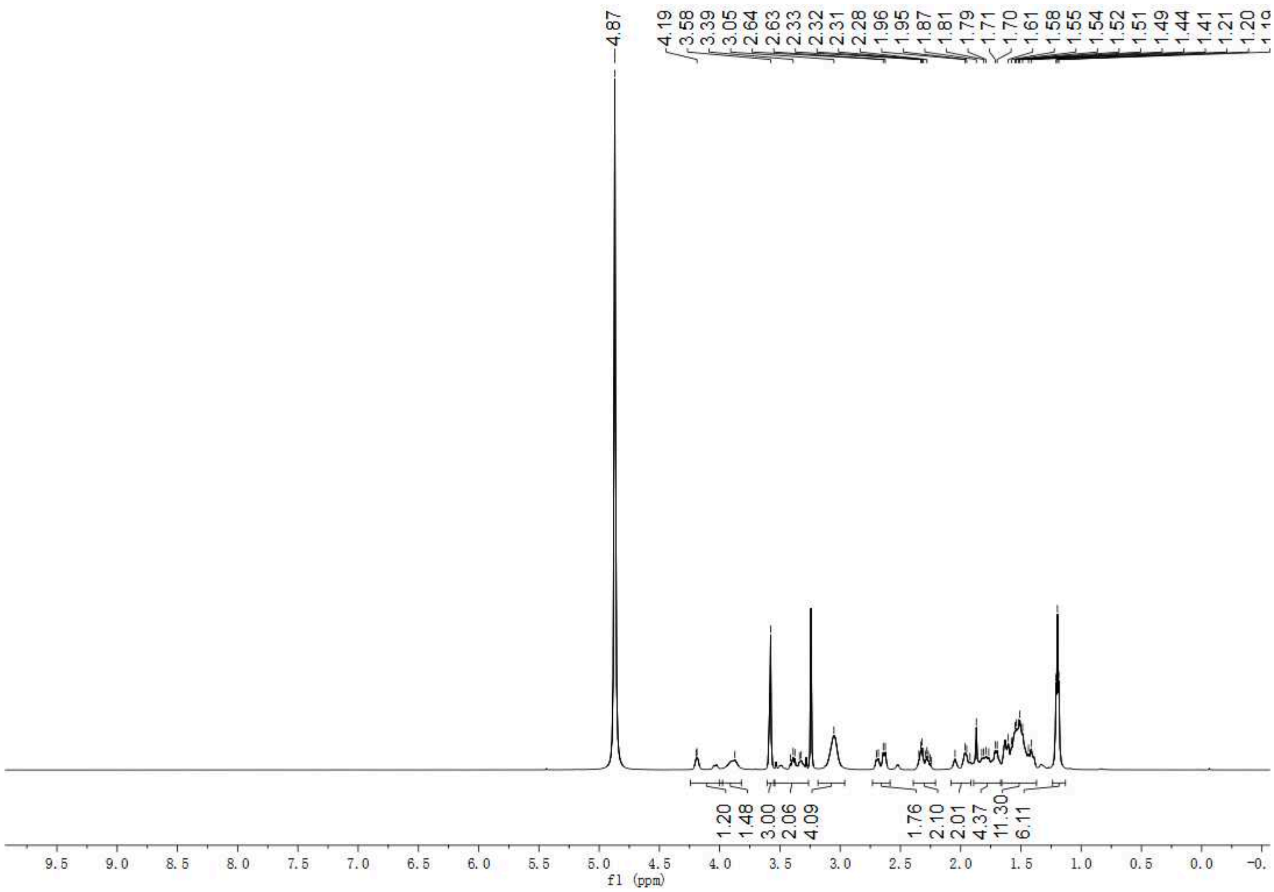

Figure S37. ^{1}H NMR spectrum (600 MHz, CD_3OD) of **2b**

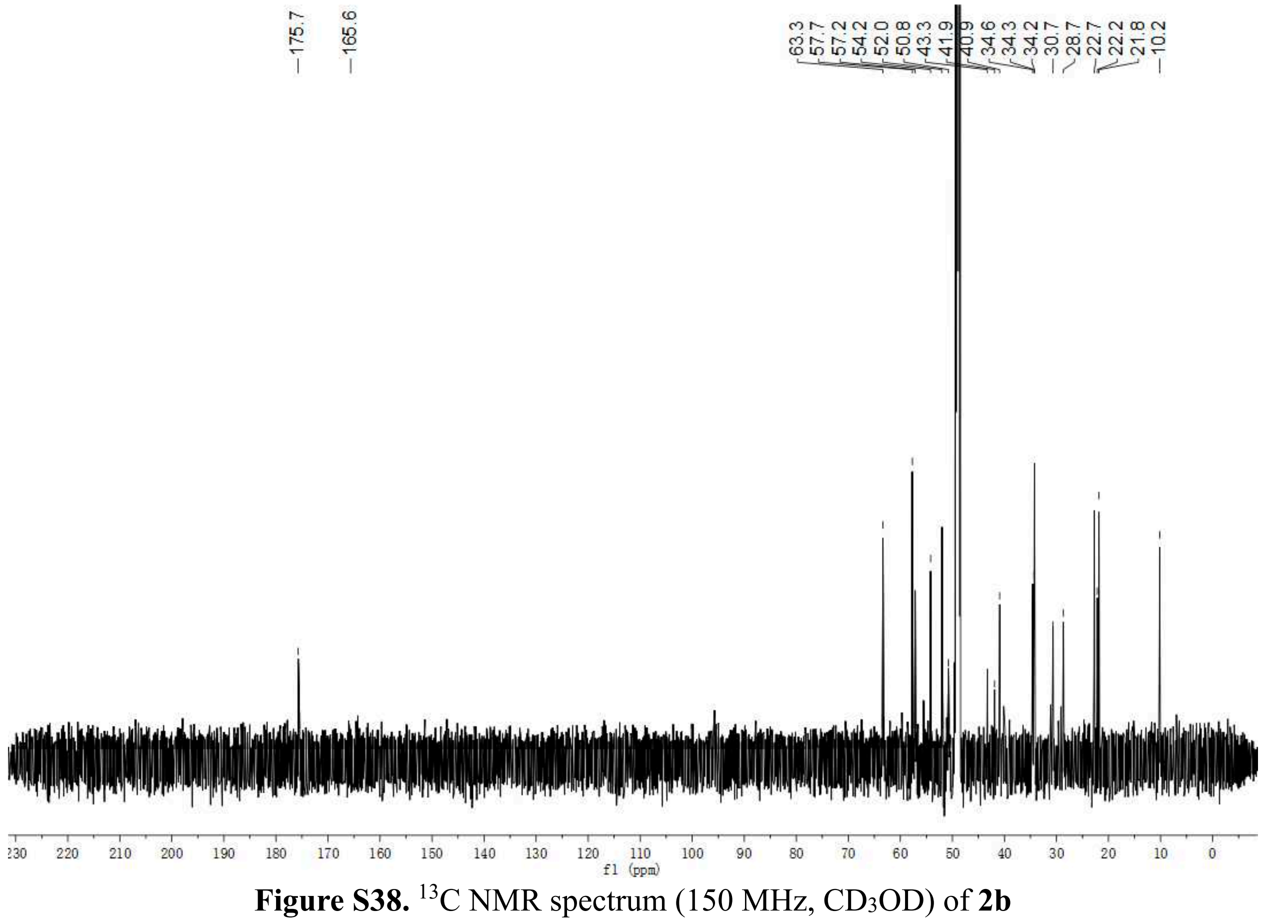

Figure S38. ^{13}C NMR spectrum (150 MHz, CD_3OD) of **2b**

Qualitative Analysis Report

**Data Filename**	B3.d	**Sample Name**	B3
**Sample Type**	Sample	**Position**	P1-F1
**Instrument Name**	Instrument 1	**User Name**	
**Acq Method**	20240114-HRMS-Pos-1.5min-5%H2O.m	**Acquired Time**	2024/4/14 12:01:12
**IRM Calibration Status**	Success	**DA Method**	Default.m
**Comment**			

**Sample Group**		**Info.**	
**Stream Name**	LC 1	**Acquisition SW Version**	6200 series TOF/6500 series Q-TOF B.06.01 (B6172 SP1)

User Spectra

Fragmentor Voltage	Collision Energy	Ionization Mode
175	0	ESI

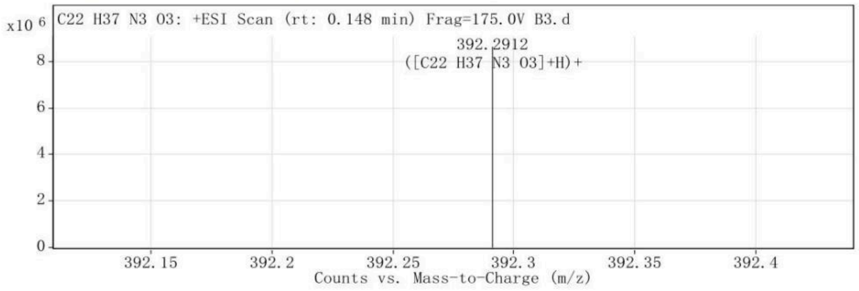

Peak List

m/z	z	Abund	Formula	Ion
84.0818		576642.38		
196.6498	2	420464.5		
249.1966	1	308941.53		
281.2239	1	812067.88		
392.2912		8638473	C22 H37 N3 O3	(M+H)+
392.4574		469221.69		
393.2961	1	5061445.5		
393.4614	1	279941.16		
394.301	1	1169164.88		
395.3077	1	329292.88		

Formula Calculator Element Limits

Element	Min	Max
C	3	60
H	0	120
O	0	30
N	0	30
S	0	5
Cl	0	3

Formula Calculator Results

Formula	Best	Mass	Tgt Mass	Diff (ppm)	Ion Species	Score
C22 H37 N3 O3	TRUE	391.2839	391.2835	-1.08	C22 H38 N3 O3	47.14

--- End Of Report ---

Figure S39. HRESIMS analysis of **2c**

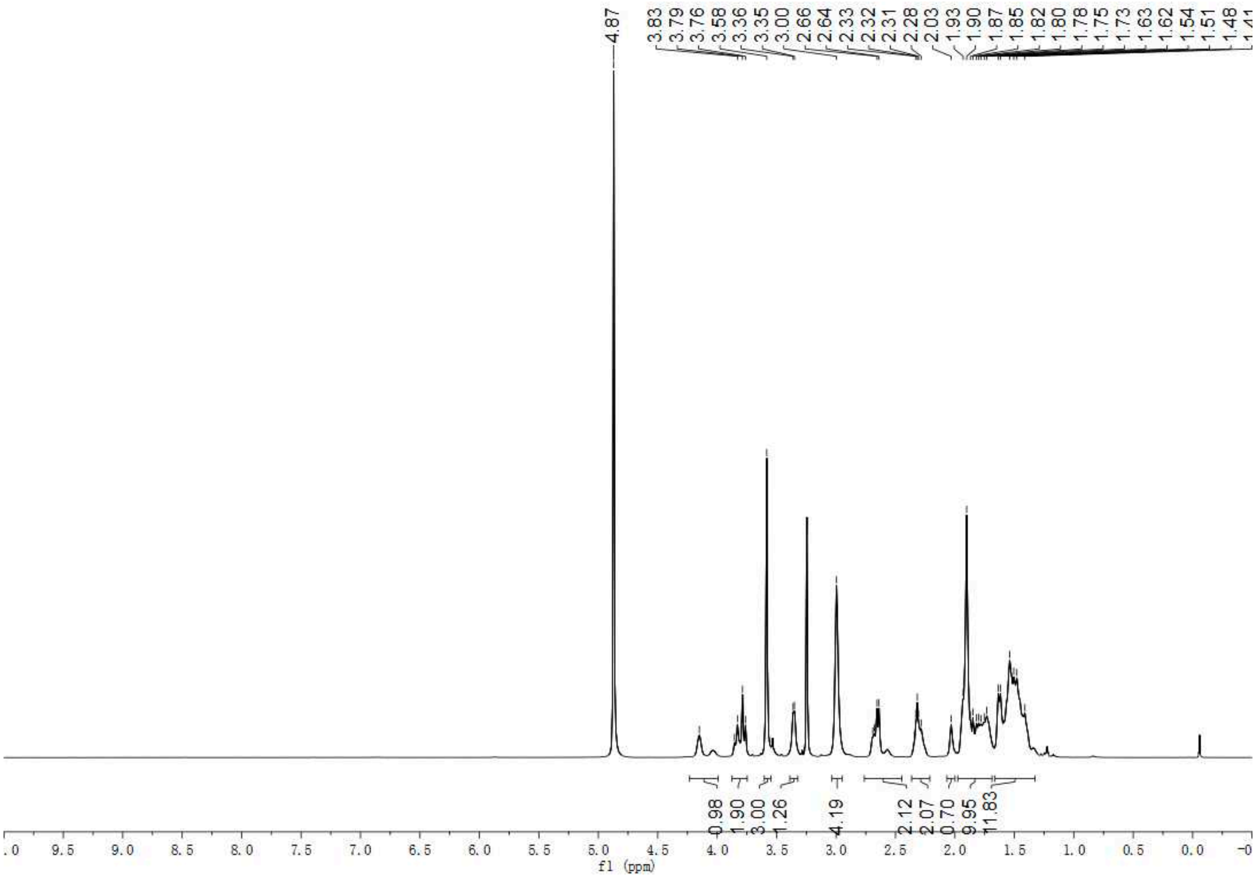

Figure S40. ^{1}H NMR spectrum (600 MHz, CD_3OD) of **2c**

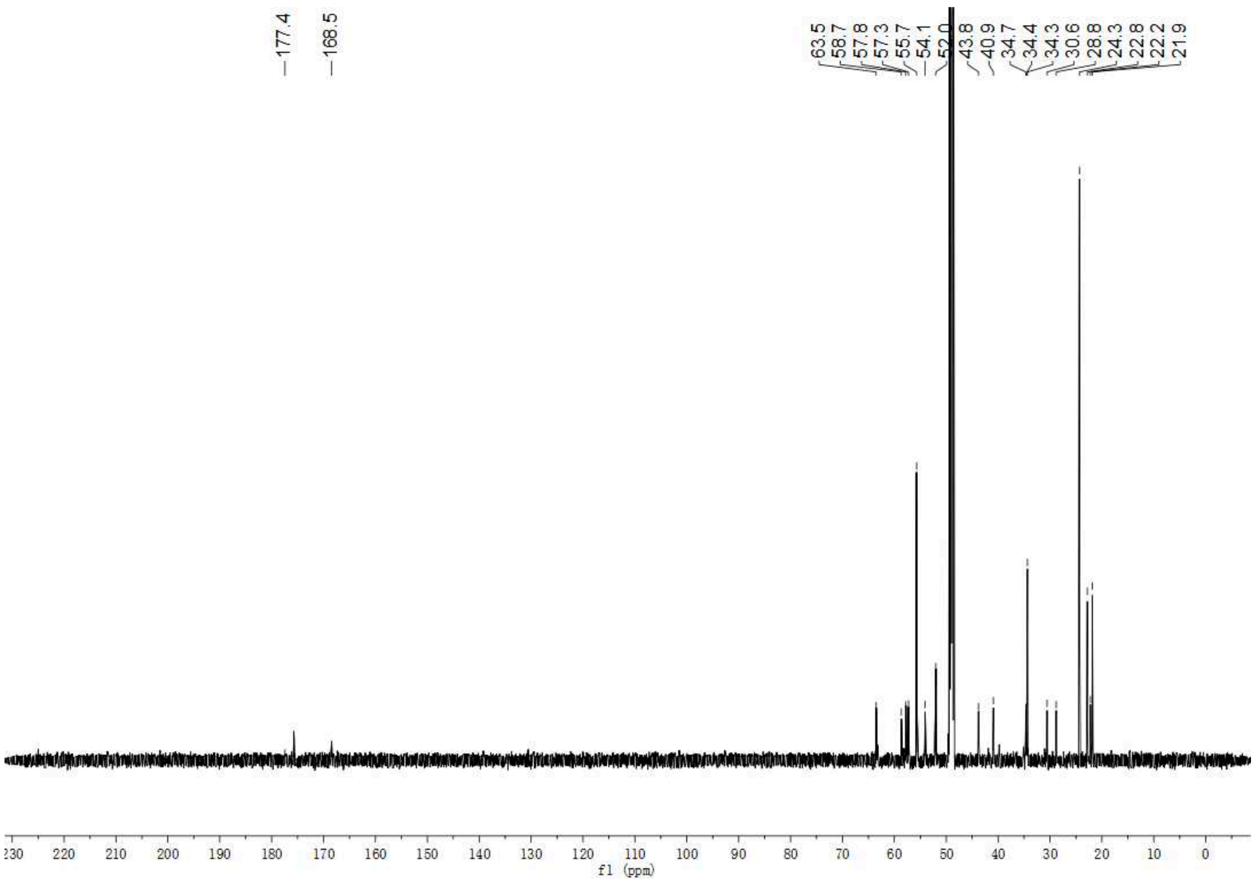

Figure S41. ^{13}C NMR spectrum (150 MHz, CD_3OD) of **2c**

Qualitative Analysis Report

**Data Filename**	B6.d	**Sample Name**	B6
**Sample Type**	Sample	**Position**	P1-F4
**Instrument Name**	Instrument 1	**User Name**	
**Acq Method**	20231128-LCMS-RC-WCX-Pos-Full-1.m	**Acquired Time**	2024/4/14 12:08:55
**IRM Calibration Status**	Success	**DA Method**	Default.m
**Comment**			

**Sample Group**		**Info.**	
**Stream Name**	LC 1	**Acquisition SW Version**	6200 series TOF/6500 series Q-TOF B.06.01 (B6172 SP1)

User Spectra

Fragmentor Voltage	Collision Energy	Ionization Mode
185	10	ESI

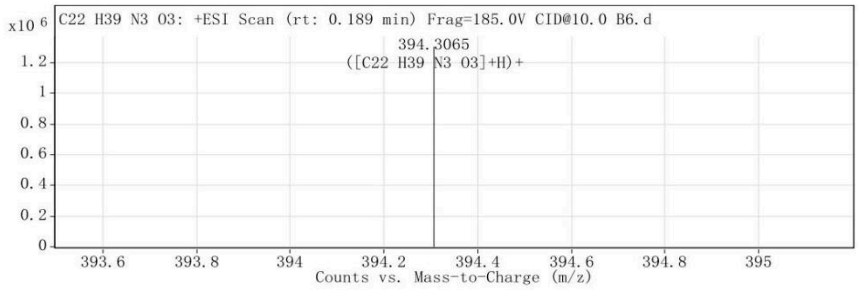

Peak List

m/z	z	Abund	Formula	Ion
84.9602		50415.46		
86.0971		48962.5		
249.196	1	66306.92		
281.2225	1	230780.55		
394.3065	1	1303589.88	C22 H39 N3 O3	(M+H)+
395.3107	1	454845.75	C22 H39 N3 O3	(M+H)+
396.3142	1	71342.52	C22 H39 N3 O3	(M+H)+
397.325	1	161539.17		
416.2887	1	69099.59		
442.283	1	54478.34		

Formula Calculator Element Limits

Element	Min	Max
C	3	60
H	0	120
O	0	30
N	0	30
S	0	5
Cl	0	3

Formula Calculator Results

Formula	Best	Mass	Tgt Mass	Diff (ppm)	Ion Species	Score
C22 H39 N3 O3	TRUE	393.2996	393.2991	-1.06	C22 H40 N3 O3	86.53

--- End Of Report ---

Figure S42. HRESIMS analysis of **2d**

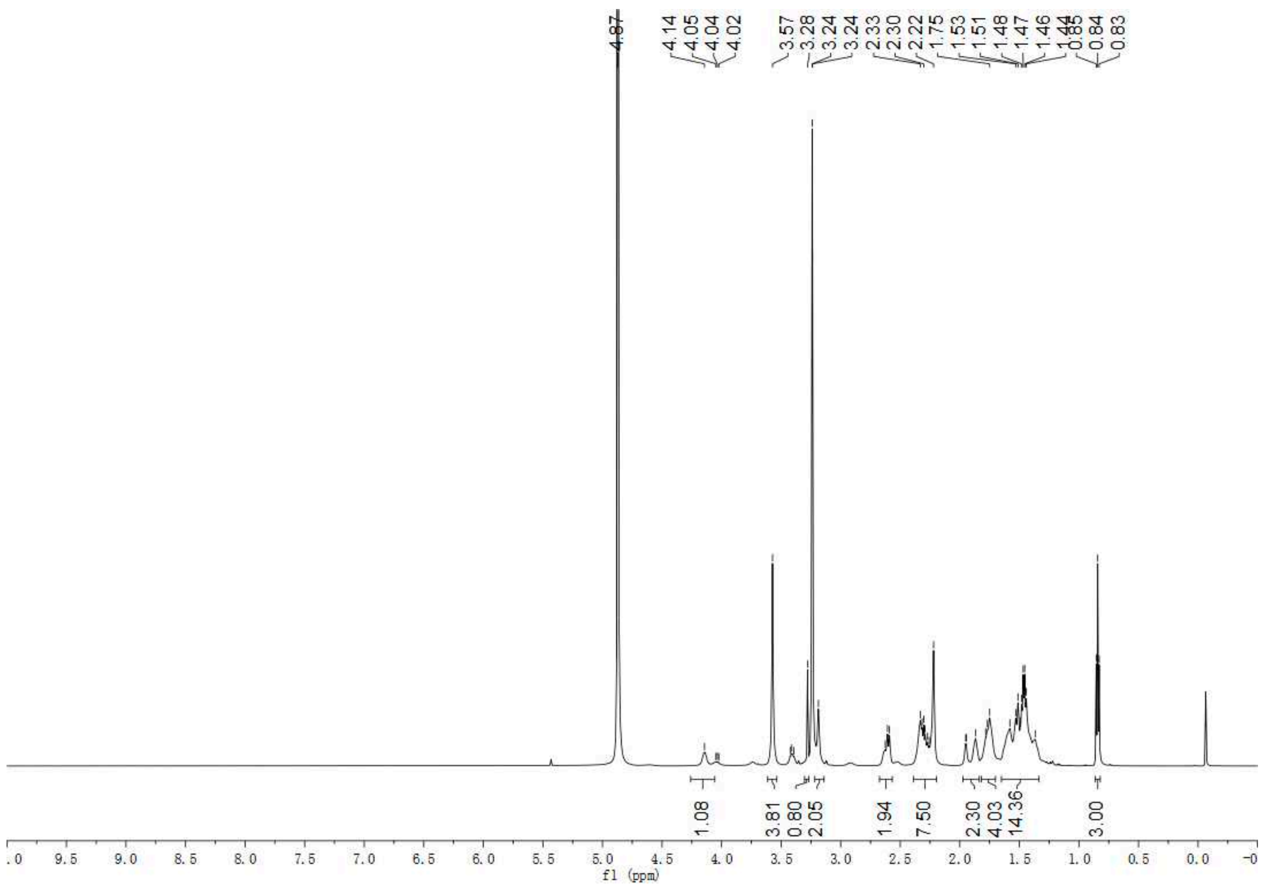

Figure S43. ^{1}H NMR spectrum (600 MHz, CD_3OD) of **2d**

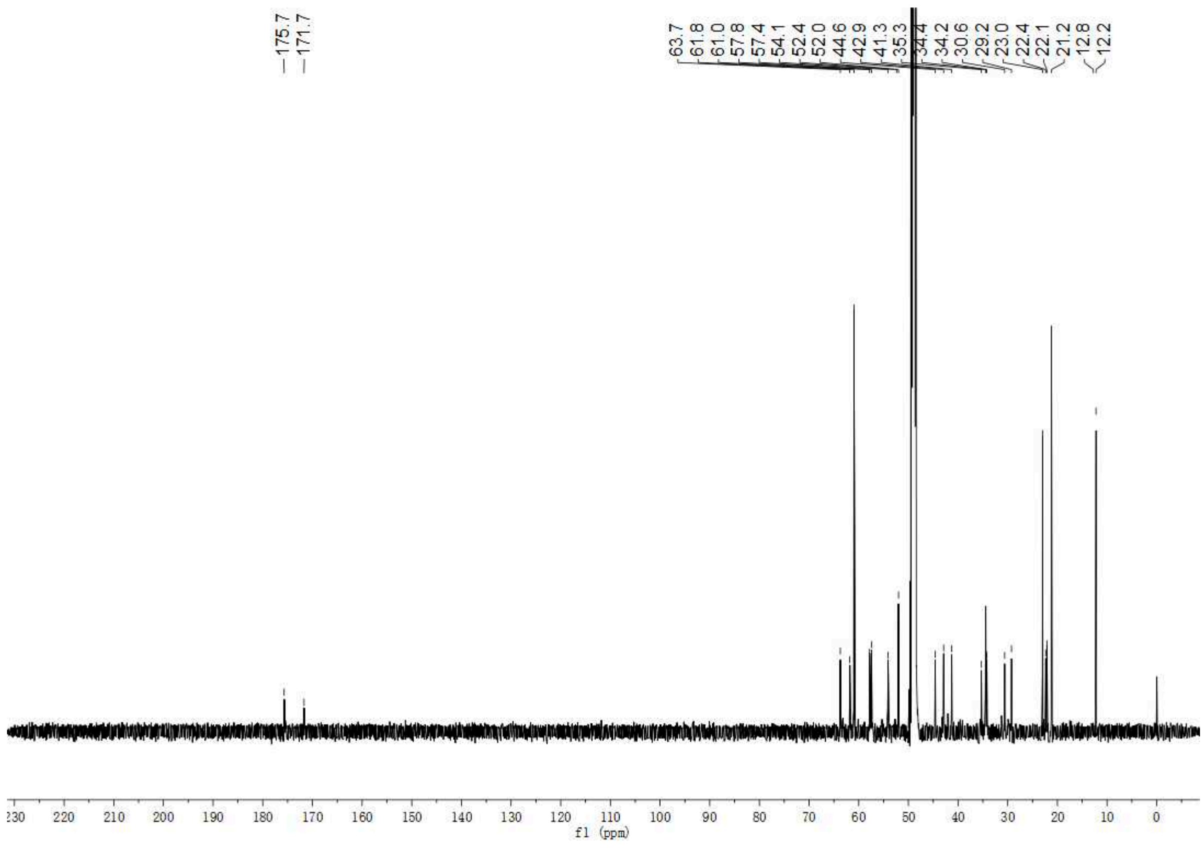

Figure S44. ^{13}C NMR spectrum (150 MHz, CD_3OD) of **2d**

Qualitative Analysis Report

**Data Filename**	B7.d	**Sample Name**	B7
**Sample Type**	Sample	**Position**	P1-F5
**Instrument Name**	Instrument 1	**User Name**	
**Acq Method**	20240114-HRMS-Pos-1.5min-5%H2O.m	**Acquired Time**	2024/4/14 12:32:41
**IRM Calibration Status**	Success	**DA Method**	Default.m
**Comment**			

Sample Group
Stream Name LC 1

Info.
Acquisition SW Version 6200 series TOF/6500 series Q-TOF B.06.01 (B6172 SP1)

User Spectra

Fragmentor Voltage	Collision Energy	Ionization Mode
175	0	ESI

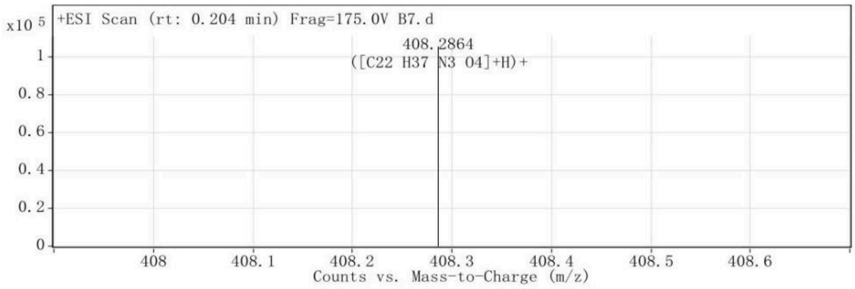

Peak List

m/z	z	Abund	Formula	Ion
72.987		7164.01		
84.9598		19290.52		
100.0757		9217.75		
116.976		15748.07		
151.0002		6818.37		
162.9731	1	17209.51		
194.9897	1	20288.97		
396.8026	2	14633.16		
408.2864	1	105547.02	C22 H37 N3 O4	(M+H)+
409.2897	1	25243.56	C22 H37 N3 O4	(M+H)+

Formula Calculator Element Limits

Element	Min	Max
C	3	60
H	0	120
O	0	30
N	0	30
S	0	5
Cl	0	3

Formula Calculator Results

Formula	Best	Mass	Tgt Mass	Diff (ppm)	Ion Species	Score
C22 H37 N3 O4	TRUE	407.279	407.2784	-1.54	C22 H38 N3 O4	97.86

--- End Of Report ---

Agilent Technologies Page 1 of 1 Printed at: 21:29 on: 2024/11/17

Figure S45. HRESIMS analysis of **2e**

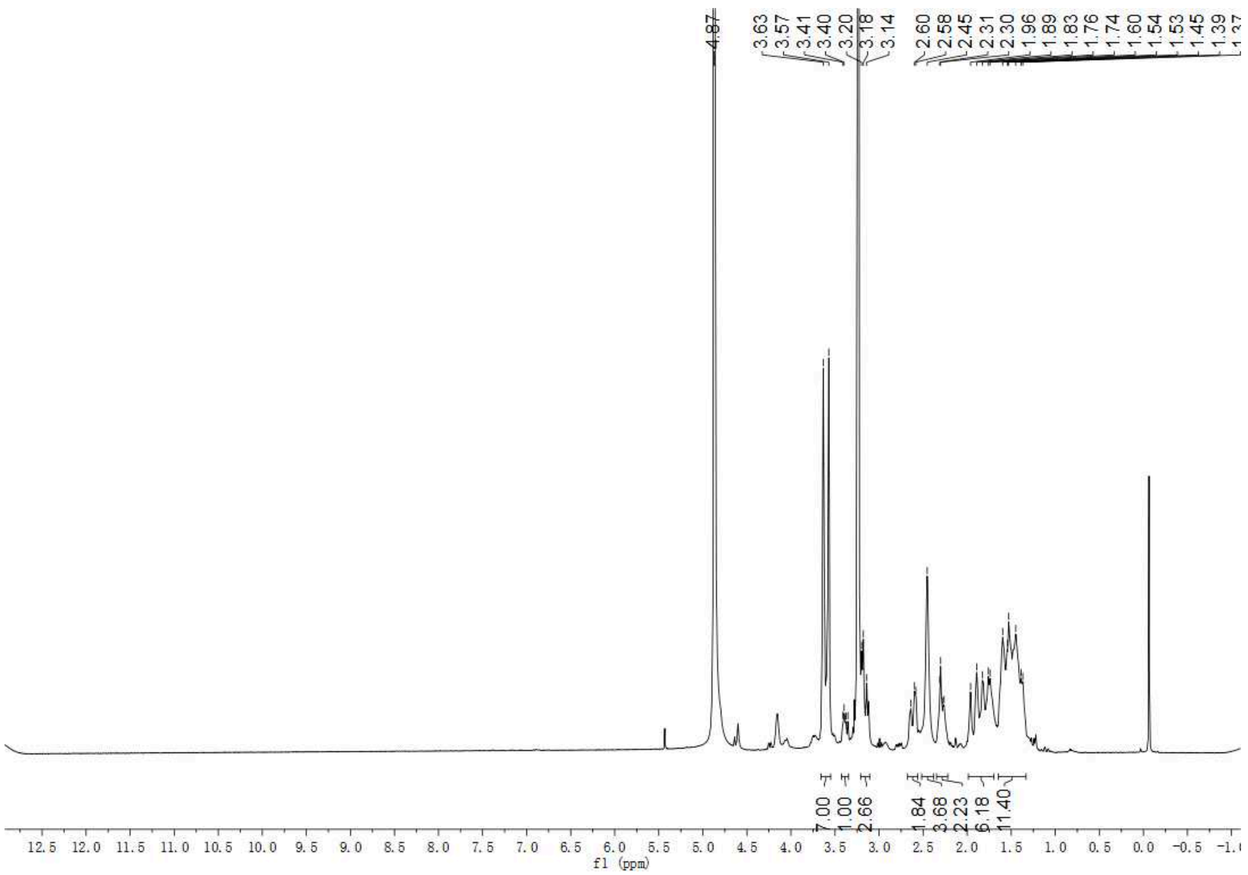

Figure S46. ^{1}H NMR spectrum (600 MHz, CD_3OD) of **2e**

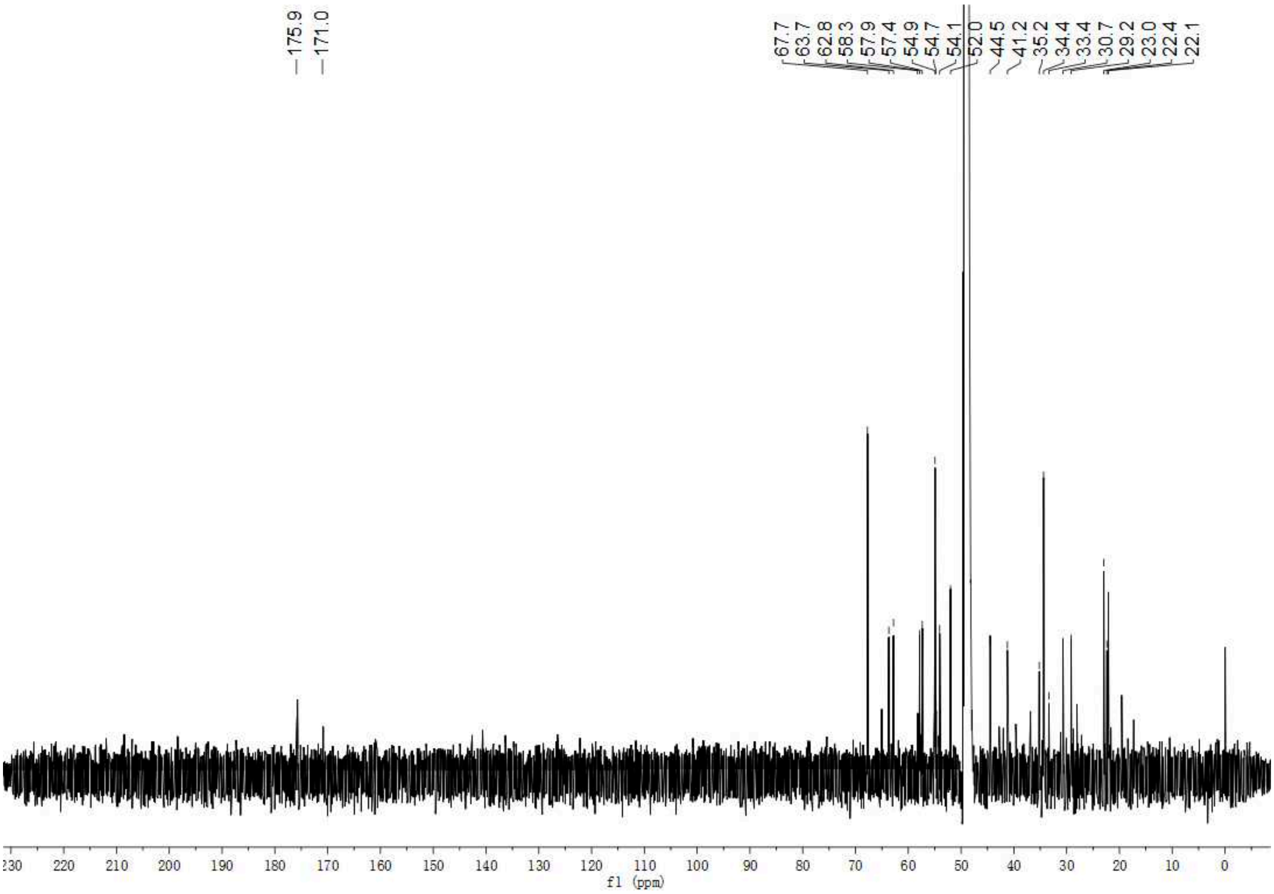

Figure S47. ^{13}C NMR spectrum (150 MHz, CD_3OD) of **2e**

Qualitative Analysis Report

**Data Filename**	C1.d	**Sample Name**	C1
**Sample Type**	Sample	**Position**	P1-A7
**Instrument Name**	Instrument 1	**User Name**	
**Acq Method**	20200905-HRMS(+).m	**Acquired Time**	2024/4/14 13:10:01
**IRM Calibration Status**	Success	**DA Method**	Default.m
**Comment**			

**Sample Group**		**Info.**	
**Stream Name**	LC 1	**Acquisition SW Version**	6200 series TOF/6500 series Q-TOF B.06.01 (B6172 SP1)

User Spectra

Fragmentor Voltage	Collision Energy	Ionization Mode
175	0	ESI

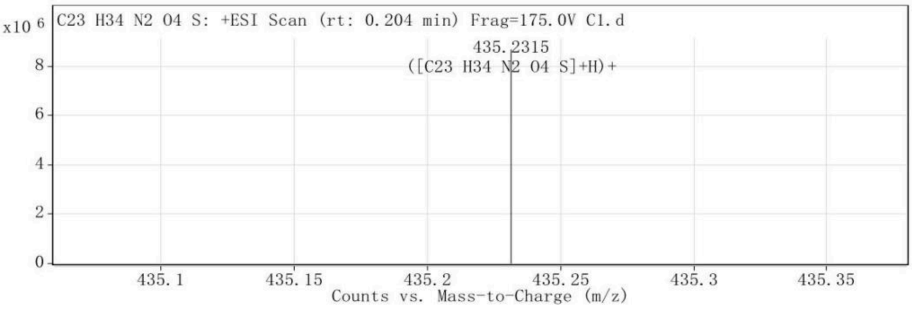

Peak List

m/z	z	Abund	Formula	Ion
84.9606		60971.59		
339.2283	1	36978.54		
433.2165	1	166401.47		
434.219	1	41615.82		
435.2315	1	8693489	C23 H34 N2 O4 S	(M+H)+
435.4059	1	465778.28		
436.2349	1	2286148	C23 H34 N2 O4 S	(M+H)+
436.4095	1	123290.47		
437.2336	1	742055.19	C23 H34 N2 O4 S	(M+H)+
438.2344	1	129460.24	C23 H34 N2 O4 S	(M+H)+

Formula Calculator Element Limits

Element	Min	Max
C	3	60
H	0	120
O	0	30
N	0	30
S	0	5
Cl	0	3

Formula Calculator Results

Formula	Best	Mass	Tgt Mass	Diff (ppm)	Ion Species	Score
C23 H34 N2 O4 S	TRUE	434.2244	434.2239	-1.12	C23 H35 N2 O4 S	98.76

--- End Of Report ---

Figure S48. HRESIMS analysis of **3a**

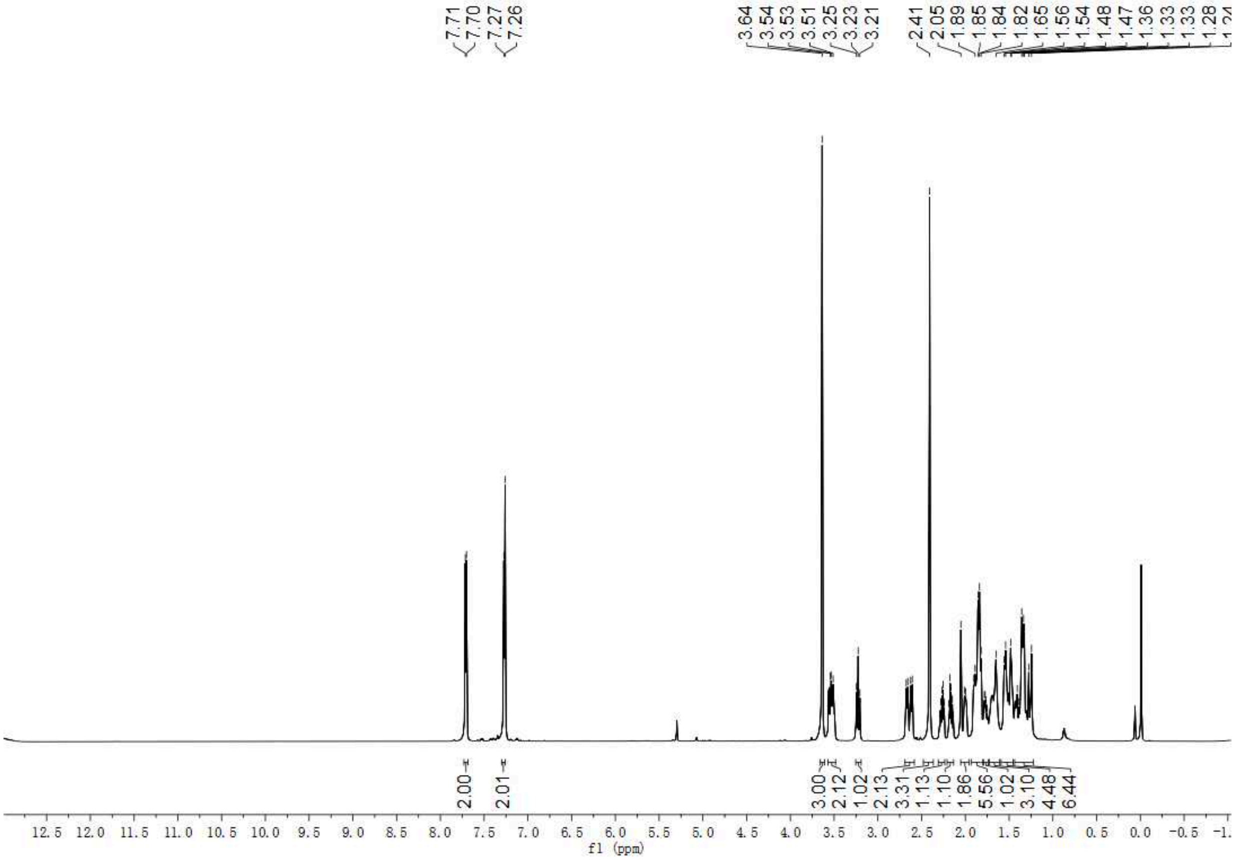

Figure S49. ^{1}H NMR spectrum (600 MHz, $CDCl_3$) of **3a**

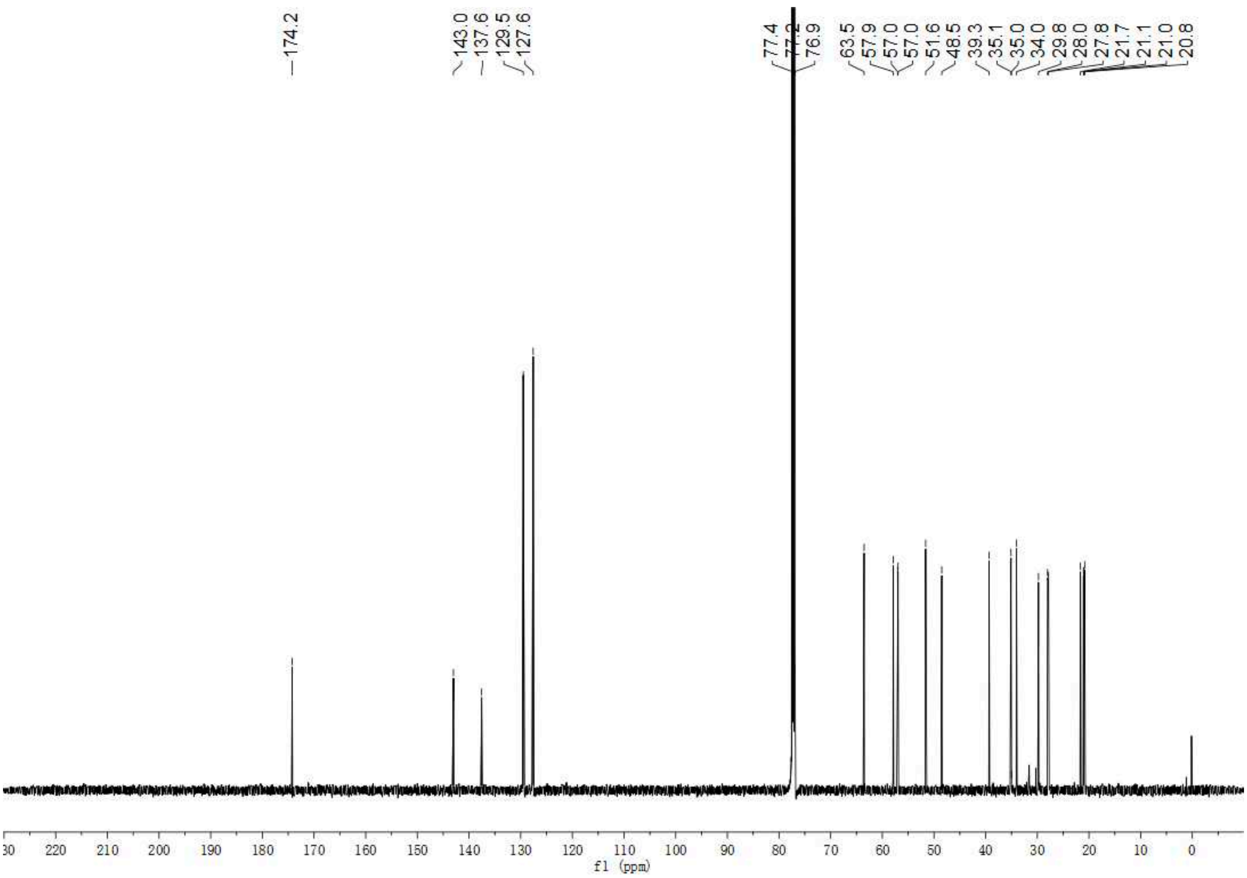

Figure S50. ^{13}C NMR spectrum (150 MHz, $CDCl_3$) of **3a**

Qualitative Analysis Report

**Data Filename**	C2.d	**Sample Name**	C2
**Sample Type**	Sample	**Position**	P1-A8
**Instrument Name**	Instrument 1	**User Name**	
**Acq Method**	20200905-HRMS(+).m	**Acquired Time**	2024/4/14 13:12:43
**IRM Calibration Status**	Success	**DA Method**	Default.m
**Comment**			

**Sample Group**		**Info.**	
**Stream Name**	LC 1	**Acquisition SW Version**	6200 series TOF/6500 series Q-TOF B.06.01 (B6172 SP1)

User Spectra

Fragmentor Voltage	Collision Energy	Ionization Mode
175	0	ESI

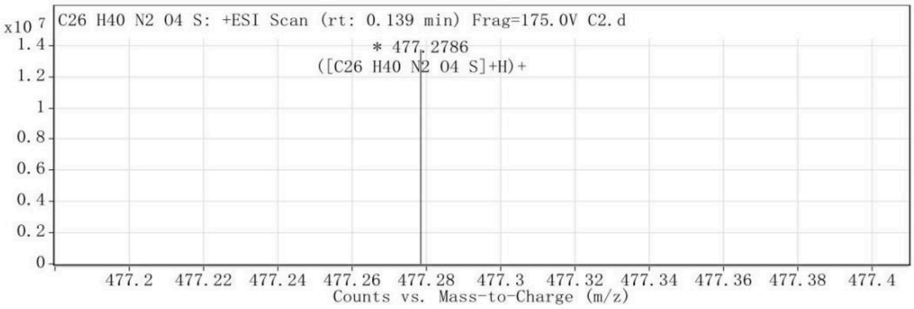

Peak List

m/z	z	Abund	Formula	Ion
475.264	1	1358336.25		
476.2669	1	437798.19		
477.2786	1	13745688	C26 H40 N2 O4 S	(M+H)+
477.4596	1	744123.19		
478.2815	1	4208602.5	C26 H40 N2 O4 S	(M+H)+
478.4627	1	278437.53		
479.2811	1	1229354.5	C26 H40 N2 O4 S	(M+H)+
480.2815	1	274213.34		
487.2278	1	402125.44		
493.2737	1	200945.14		

Formula Calculator Element Limits

Element	Min	Max
C	3	60
H	0	120
O	0	30
N	0	30
S	0	5
Cl	0	3

Formula Calculator Results

Formula	Best	Mass	Tgt Mass	Diff (ppm)	Ion Species	Score
C26 H40 N2 O4 S	TRUE	476.2713	476.2709	-0.93	C26 H41 N2 O4 S	97.84

--- End Of Report ---

Figure S51. HRESIMS analysis of **3b**

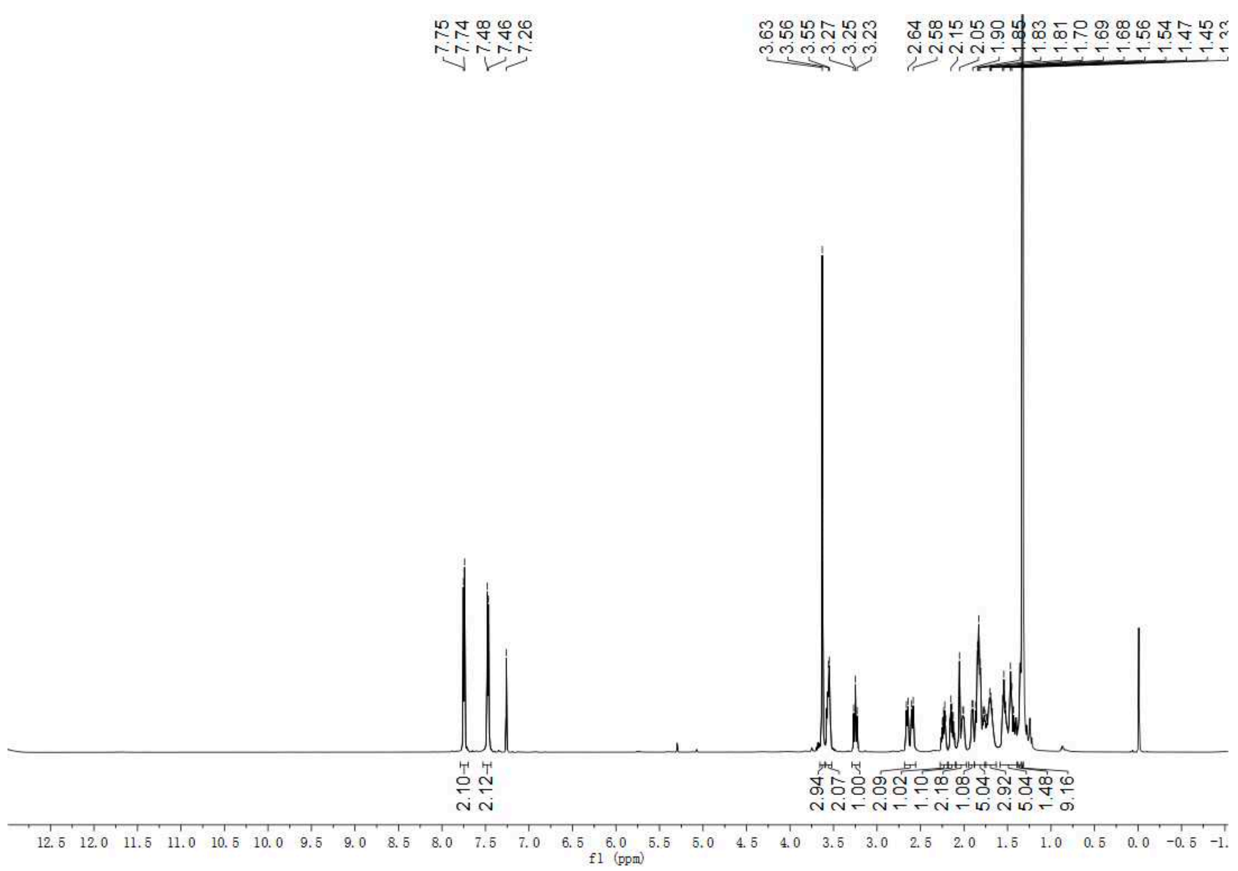

Figure S52. ^{1}H NMR spectrum (600 MHz, $CDCl_3$) of **3b**

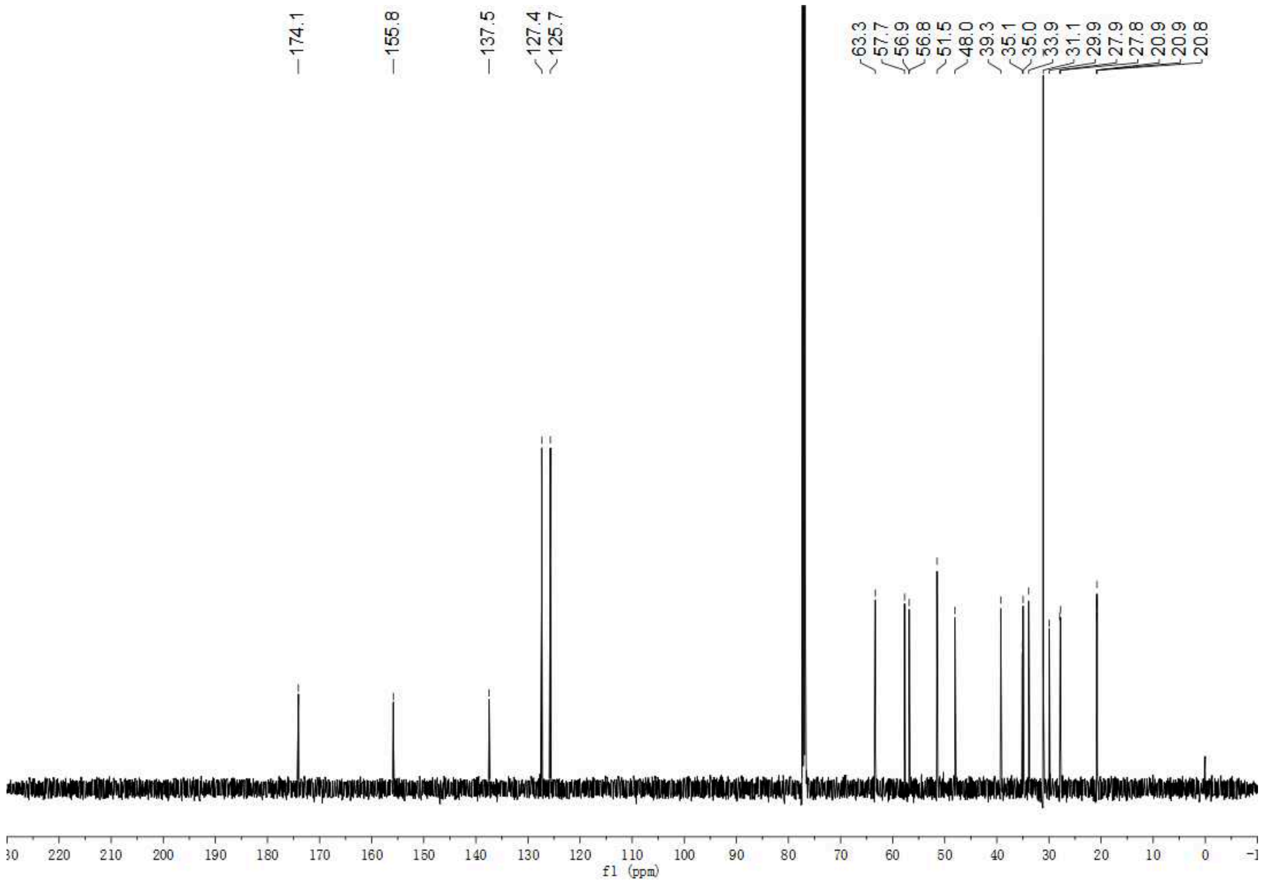

Figure S53. ^{13}C NMR spectrum (150 MHz, $CDCl_3$) of **3b**

Qualitative Analysis Report

**Data Filename**	C4.d	**Sample Name**	C4
**Sample Type**	Sample	**Position**	P1-B1
**Instrument Name**	Instrument 1	**User Name**	
**Acq Method**	20200905-HRMS(+).m	**Acquired Time**	2024/4/14 13:18:07
**IRM Calibration Status**	Success	**DA Method**	Default.m
**Comment**			

**Sample Group**		**Info.**	
**Stream Name**	LC 1	**Acquisition SW Version**	6200 series TOF/6500 series Q-TOF B.06.01 (B6172 SP1)

User Spectra

Fragmentor Voltage	Collision Energy	Ionization Mode
175	0	ESI

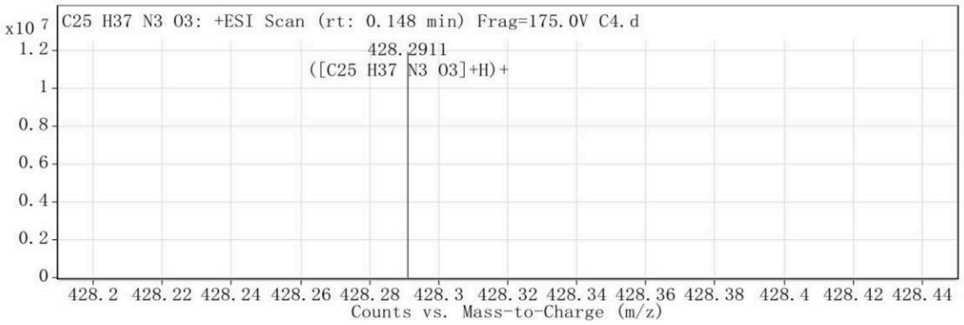

Peak List

m/z	z	Abund	Formula	Ion
148.0759	1	546615.31		
428.2911	1	11946579	C25 H37 N3 O3	(M+H)+
428.4633	1	621905.19		
429.2942	1	3407340.5	C25 H37 N3 O3	(M+H)+
429.4676	1	227101.09		
430.2981	1	581004.13	C25 H37 N3 O3	(M+H)+
663.4546	1	316098.53		
877.5563		1812062.75		
878.5624	1	981503.81		
879.5633	1	327348.56		

Formula Calculator Element Limits

Element	Min	Max
C	3	60
H	0	120
O	0	30
N	0	30
S	0	5
Cl	0	3

Formula Calculator Results

Formula	Best	Mass	Tgt Mass	Diff (ppm)	Ion Species	Score
C25 H37 N3 O3	TRUE	427.2838	427.2835	-0.77	C25 H38 N3 O3	99.6

--- End Of Report ---

Figure S54. HRESIMS analysis of **3c**

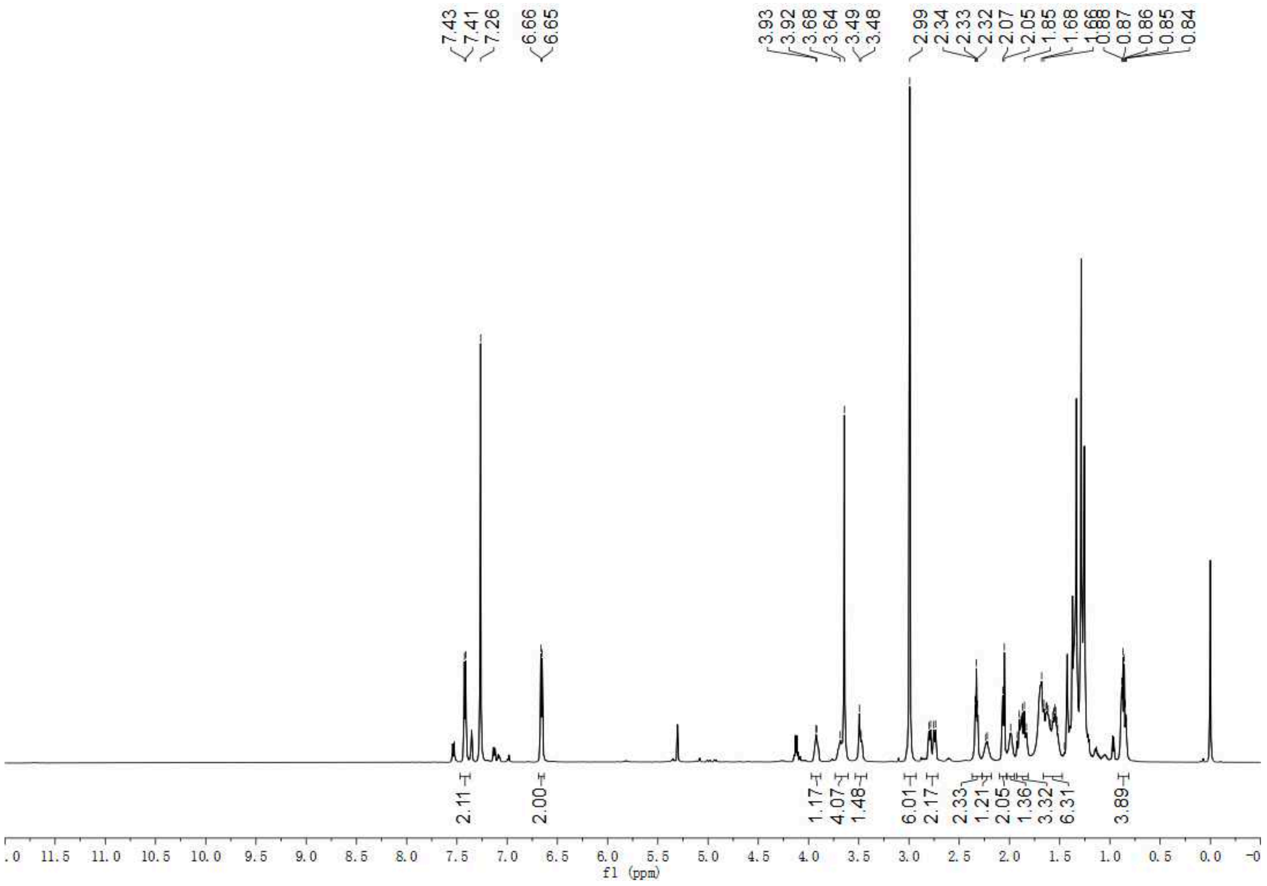

Figure S55. ^{1}H NMR spectrum (600 MHz, CD_3OD) of **3c**

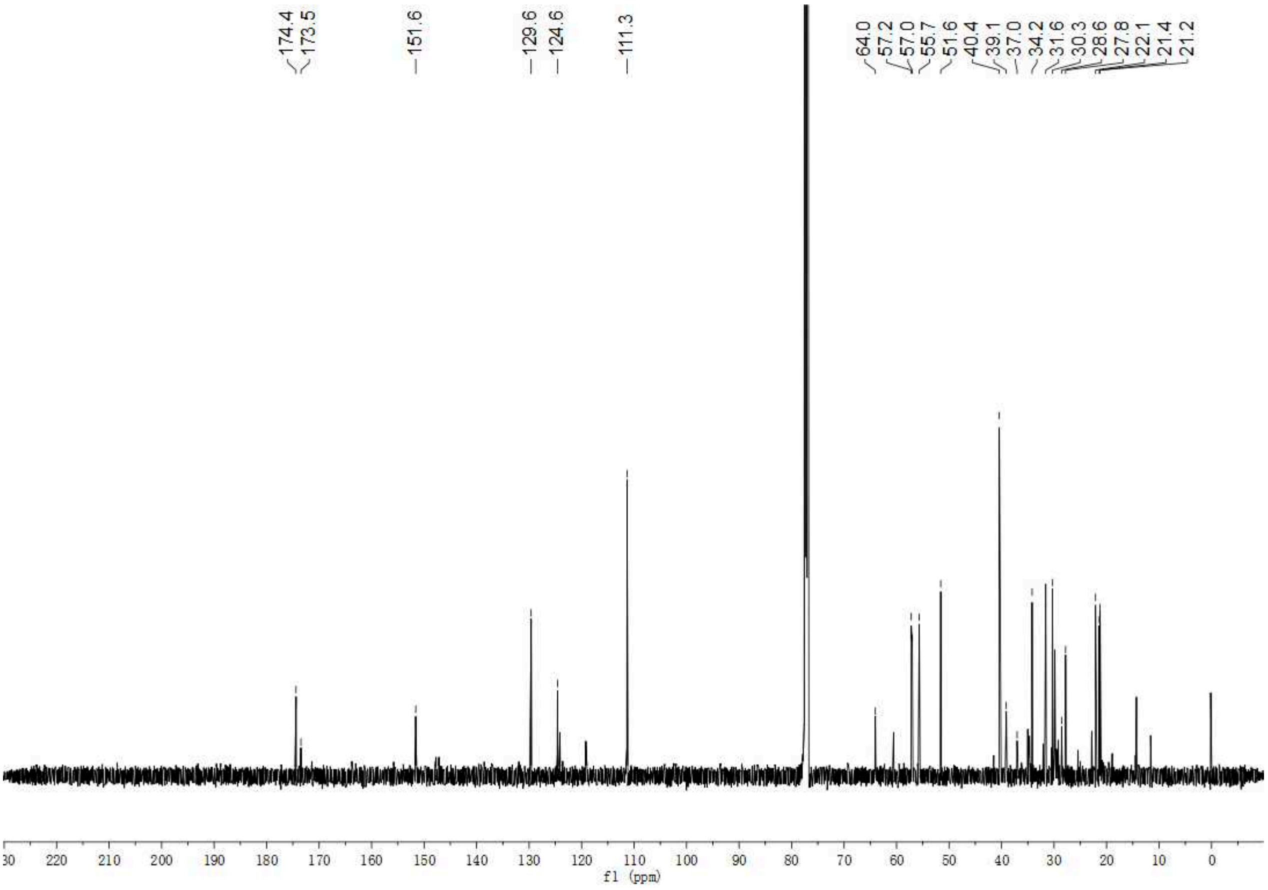

Figure S56. ^{13}C NMR spectrum (150 MHz, CD_3OD) of **3c**

Qualitative Analysis Report

**Data Filename**	A1.d	**Sample Name**	A1
**Sample Type**	Sample	**Position**	P1-E3
**Instrument Name**	Instrument 1	**User Name**	
**Acq Method**	20240114-HRMS-Pos-1.5min-5%H2O.m	**Acquired Time**	2024/4/14 11:45:11
**IRM Calibration Status**	Success	**DA Method**	Default.m
**Comment**			

**Sample Group**		**Info.**	
**Stream Name**	LC 1	**Acquisition SW Version**	6200 series TOF/6500 series Q-TOF B.06.01 (B6172 SP1)

User Spectra

Fragmentor Voltage	Collision Energy	Ionization Mode
175	0	ESI

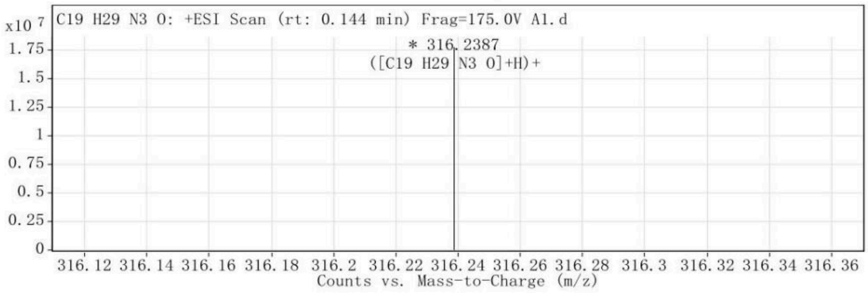

Peak List

m/z	z	Abund	Formula	Ion
184.181	1	302835.66		
229.2391	1	762663.63		
243.1496	1	673975.88		
316.2387	1	17735124	C19 H29 N3 O	(M+H)+
316.3857	1	867508.13		
317.2416	1	4010695	C19 H29 N3 O	(M+H)+
317.392	1	271435.84		
318.2454	1	482277.25	C19 H29 N3 O	(M+H)+
653.4532	1	1076166.25		
654.4556	1	518175.03		

Formula Calculator Element Limits

Element	Min	Max
C	3	60
H	0	120
O	0	30
N	0	30
S	0	5
Cl	0	3

Formula Calculator Results

Formula	Best	Mass	Tgt Mass	Diff (ppm)	Ion Species	Score
C19 H29 N3 O	TRUE	315.2314	315.2311	-1.14	C19 H30 N3 O	99.38

--- End Of Report ---

Figure S57. HRESIMS analysis of **4a**

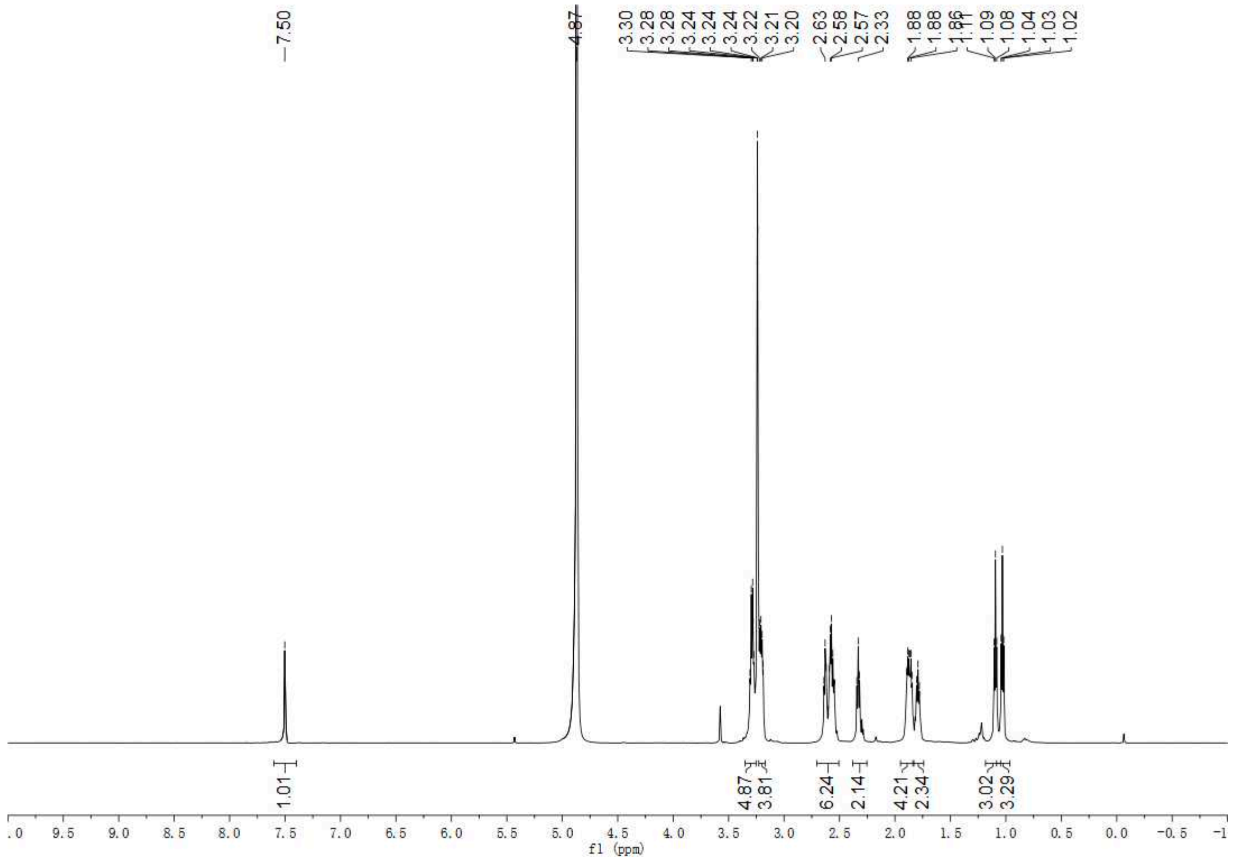

Figure S58. ^{1}H NMR spectrum (600 MHz, CD_3OD) of **4a**

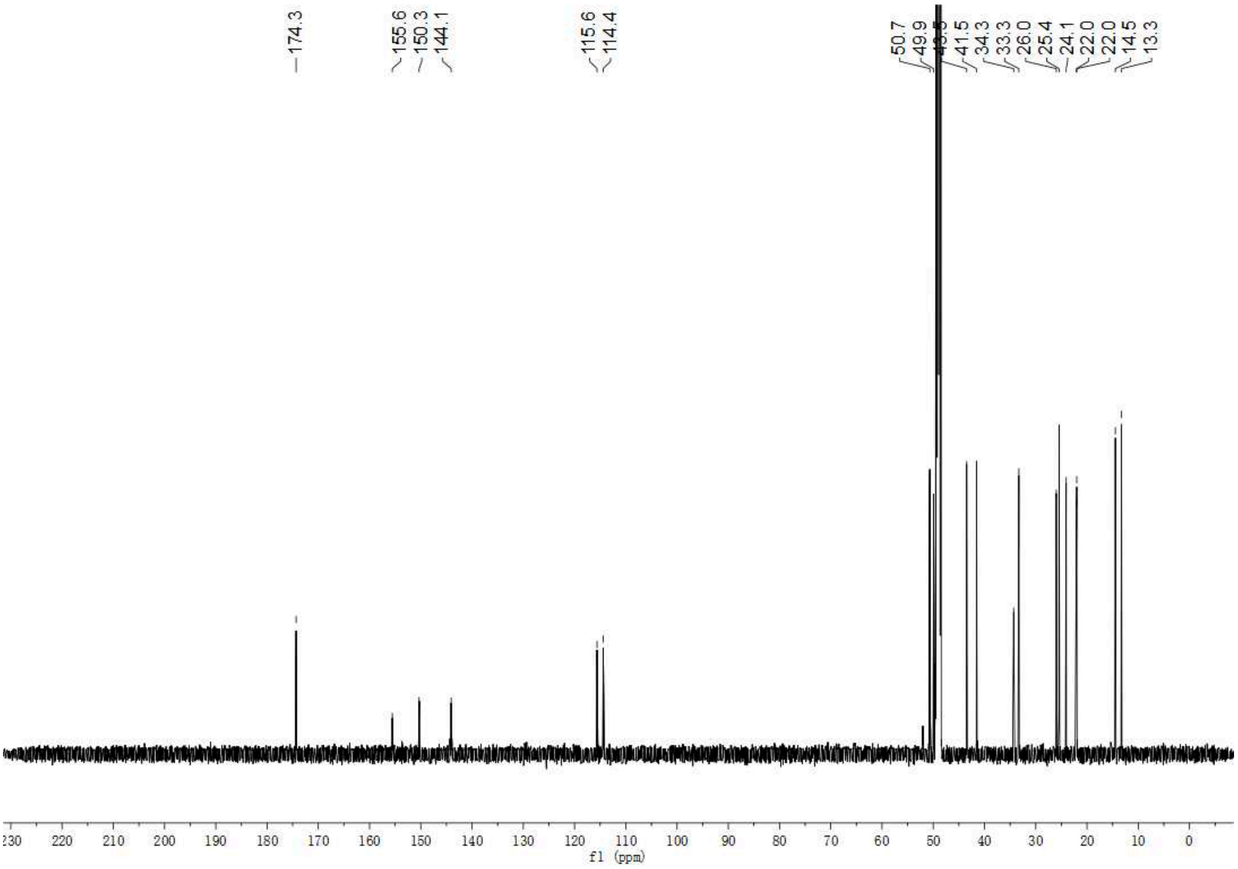

Figure S59. ^{13}C NMR spectrum (150 MHz, CD_3OD) of **4a**

Qualitative Analysis Report

**Data Filename**	A2.d	**Sample Name**	A2
**Sample Type**	Sample	**Position**	P1-E4
**Instrument Name**	Instrument 1	**User Name**	
**Acq Method**	20240114-HRMS-Pos-1.5min-5%H2O.m	**Acquired Time**	2024/4/14 11:47:28
**IRM Calibration Status**	Success	**DA Method**	Default.m
**Comment**			

**Sample Group**		**Info.**	
**Stream Name**	LC 1	**Acquisition SW Version**	6200 series TOF/6500 series Q-TOF B.06.01 (B6172 SP1)

User Spectra

Fragmentor Voltage	Collision Energy	Ionization Mode
175	0	ESI

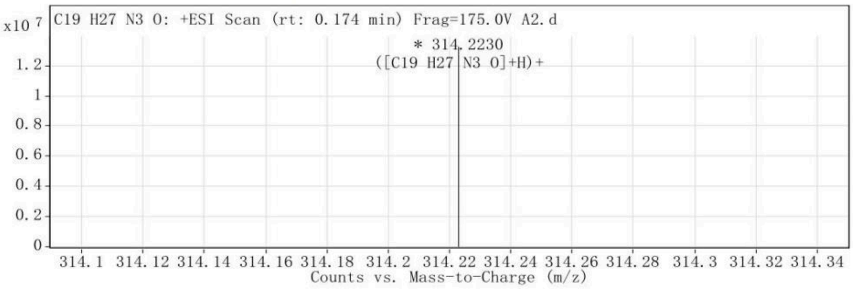

Peak List

m/z	z	Abund	Formula	Ion
243.1497	1	531954.81		
314.223	1	13244784	C19 H27 N3 O	(M+H)+
314.372	1	742546.56		
315.2261	1	2949903.5	C19 H27 N3 O	(M+H)+
315.3755	1	184567.83		
316.23	1	353338.06	C19 H27 N3 O	(M+H)+
336.2056	1	428825.69		
649.4206	1	1778349.88		
650.4259	1	774181.44		
651.4273	1	183521.14		

Formula Calculator Element Limits

Element	Min	Max
C	3	60
H	0	120
O	0	30
N	0	30
S	0	5
Cl	0	3

Formula Calculator Results

Formula	Best	Mass	Tgt Mass	Diff (ppm)	Ion Species	Score
C19 H27 N3 O	TRUE	313.2157	313.2154	-1.03	C19 H28 N3 O	99.58

--- End Of Report ---

Figure S60. HRESIMS analysis of **4b**

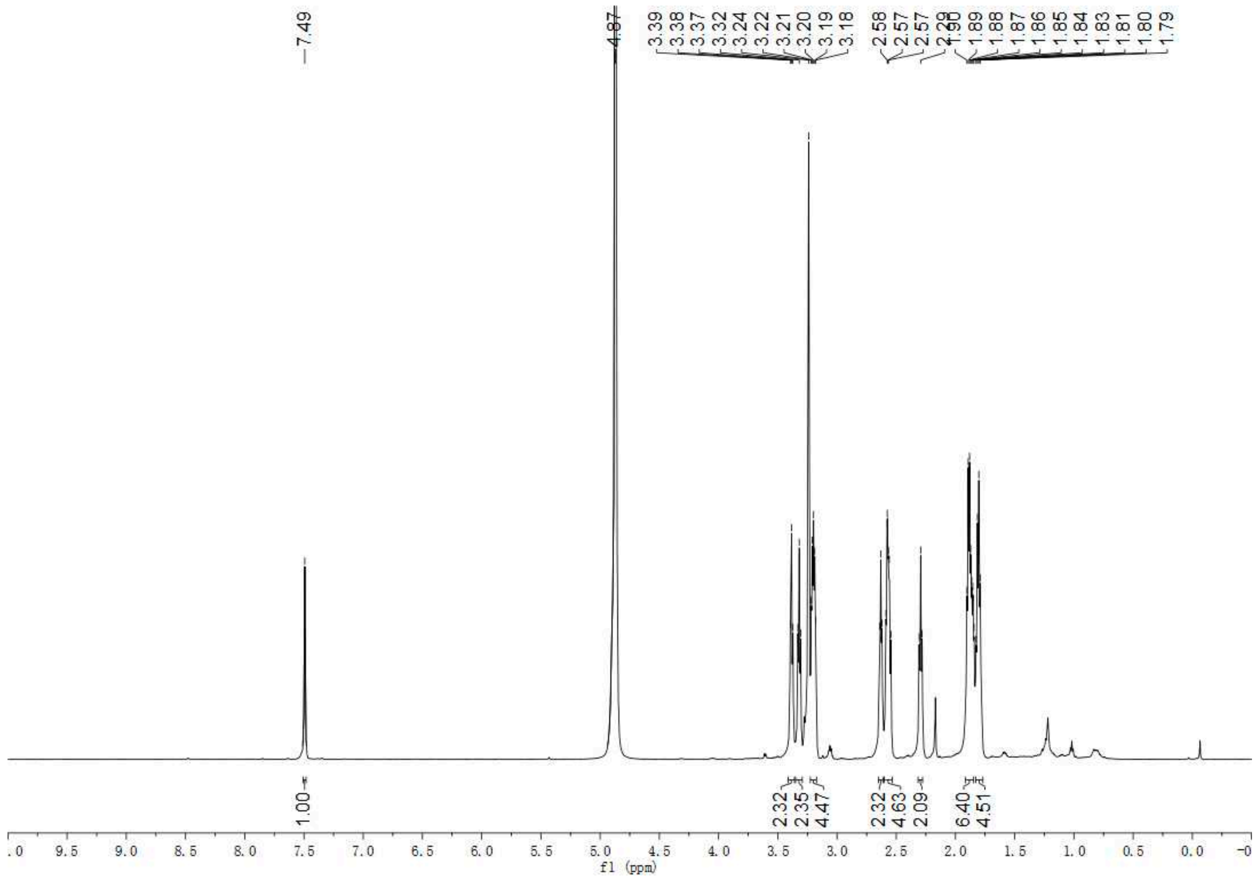

Figure S61. ^{1}H NMR spectrum (600 MHz, CD_3OD) of **4b**

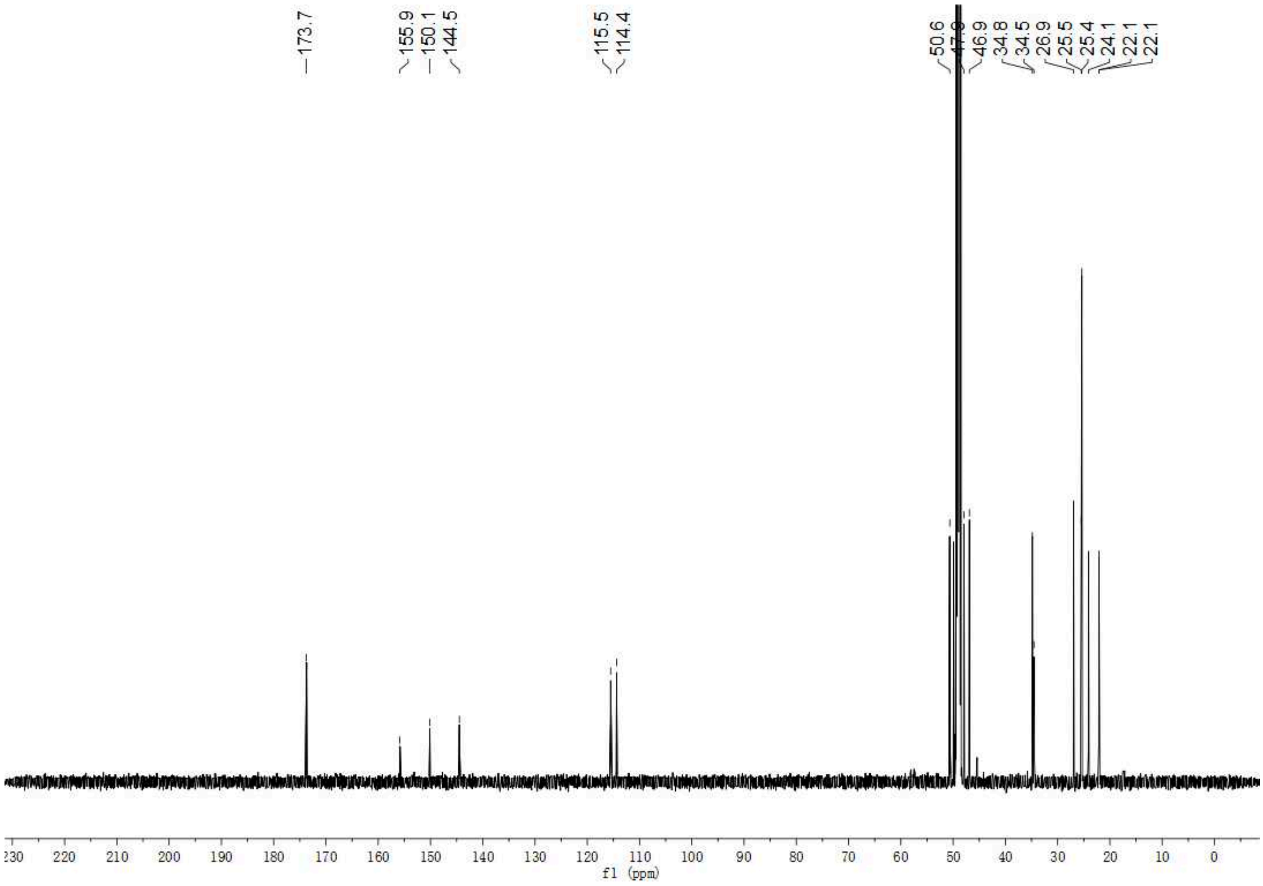

Figure S62. ^{13}C NMR spectrum (150 MHz, CD_3OD) of **4b**

Qualitative Analysis Report

**Data Filename**	A3.d	**Sample Name**	A3
**Sample Type**	Sample	**Position**	P1-E5
**Instrument Name**	Instrument 1	**User Name**	
**Acq Method**	20240114-HRMS-Pos-1.5min-5%H2O.m	**Acquired Time**	2024/4/14 11:49:45
**IRM Calibration Status**	Success	**DA Method**	Default.m
**Comment**			

**Sample Group**		**Info.**	
**Stream Name**	LC 1	**Acquisition SW Version**	6200 series TOF/6500 series Q-TOF B.06.01 (B6172 SP1)

User Spectra

Fragmentor Voltage	Collision Energy	Ionization Mode
175	0	ESI

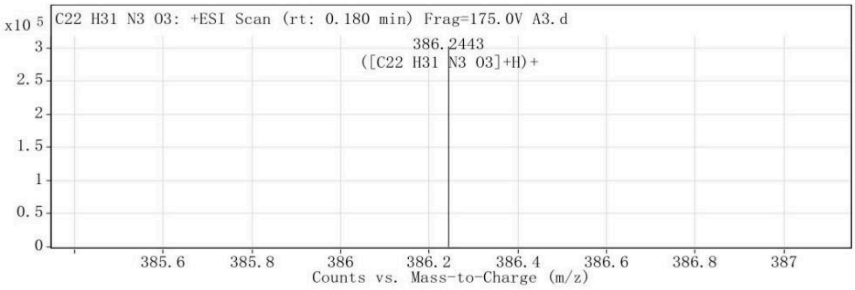

Peak List

m/z	z	Abund	Formula	Ion
86.0971	1	187334.41		
103.1234	1	424164.78		
129.1021	1	4602415		
129.2003		226728.66		
130.1053	1	328519.03		
174.1598	1	1544263.5		
196.1419	1	966152		
229.2387	1	220855.64		
299.2444	1	280104.94		
386.2443	1	302786.28	C22 H31 N3 O3	(M+H)+

Formula Calculator Element Limits

Element	Min	Max
C	3	60
H	0	120
O	0	30
N	0	30
S	0	5
Cl	0	3

Formula Calculator Results

Formula	Best	Mass	Tgt Mass	Diff (ppm)	Ion Species	Score
C22 H31 N3 O3	TRUE	385.237	385.2365	-1.21	C22 H32 N3 O3	98.02

--- End Of Report ---

Figure S63. HRESIMS analysis of **4c**

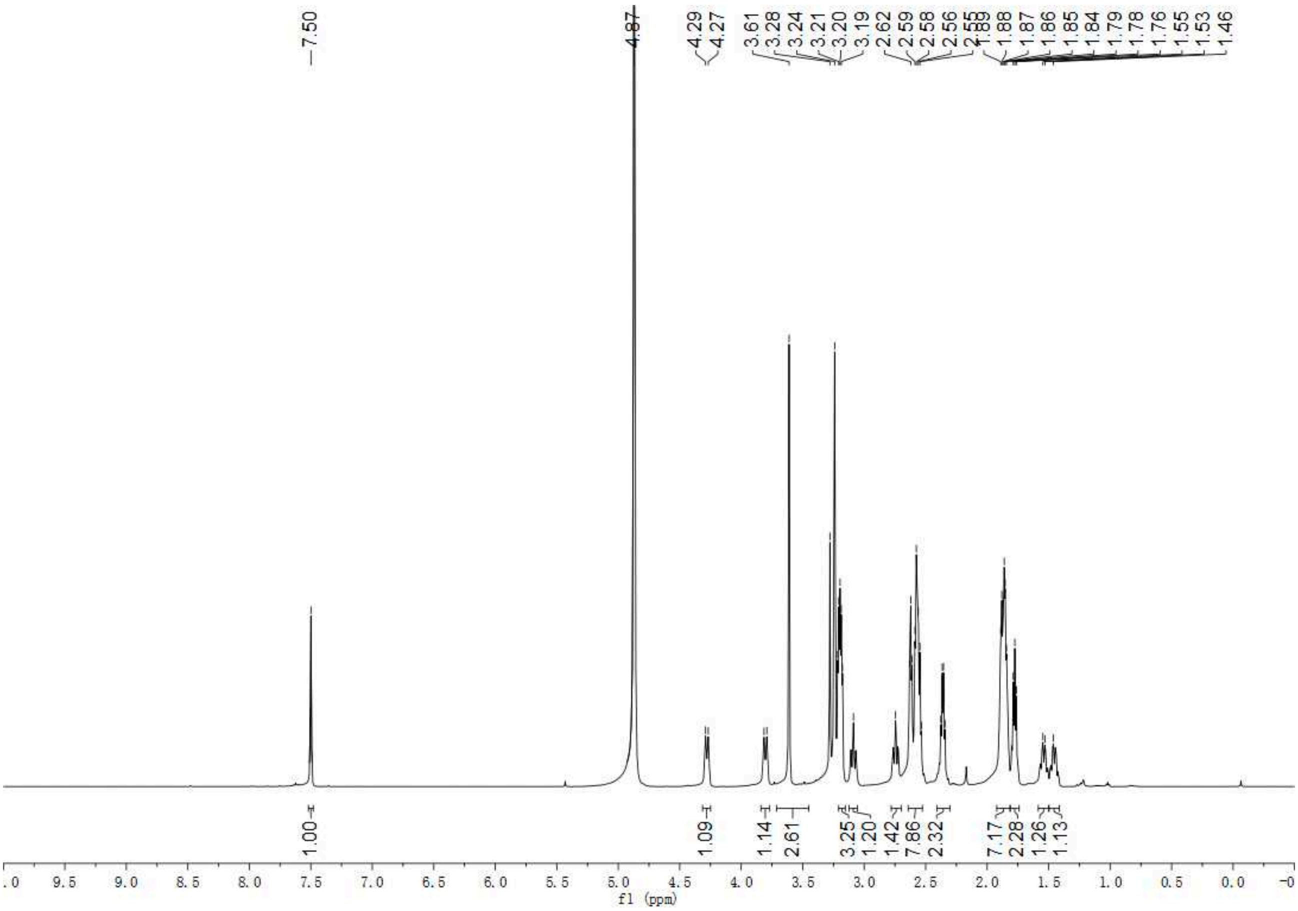

Figure S64. ^{1}H NMR spectrum (600 MHz, CD_3OD) of **4c**

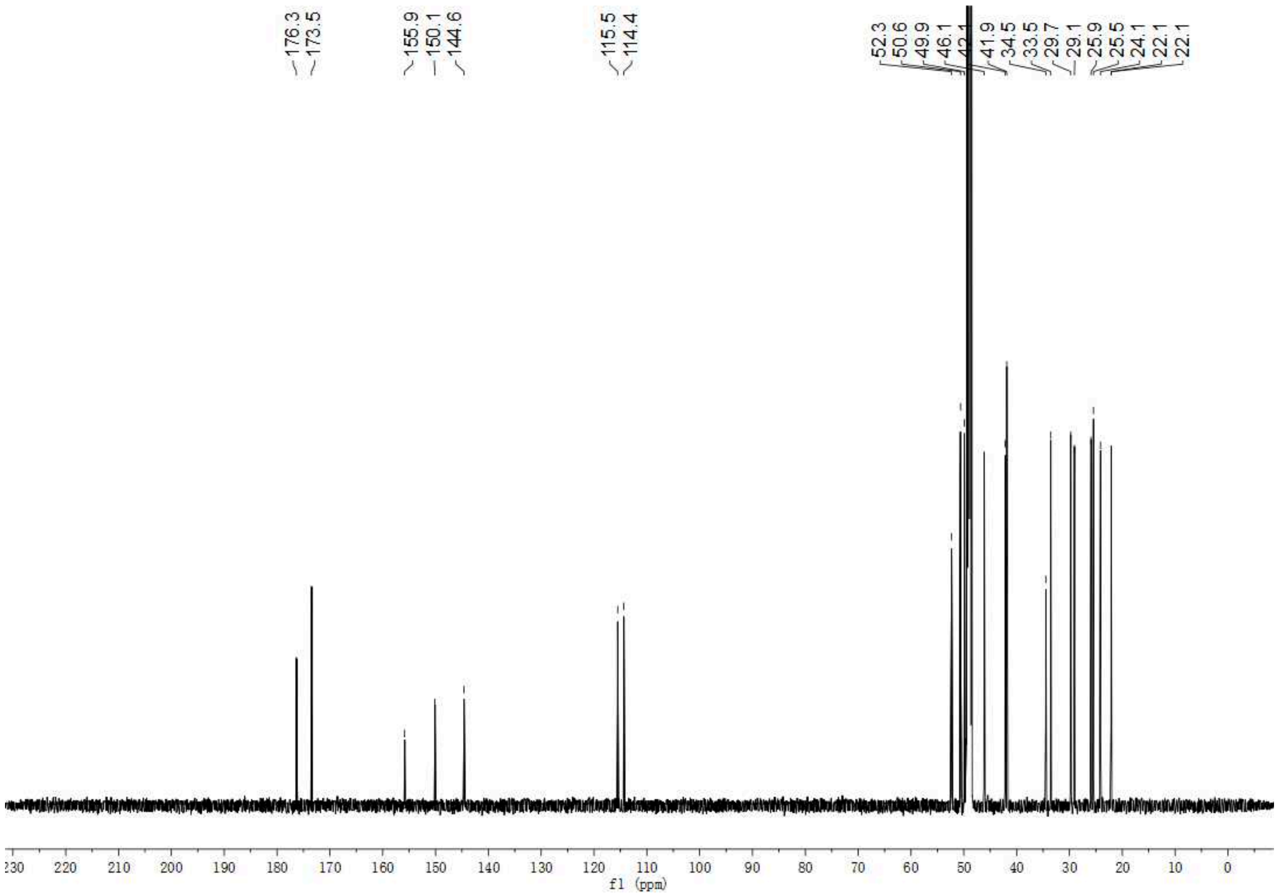

Figure S65. ^{13}C NMR spectrum (150 MHz, CD_3OD) of **4c**

Qualitative Analysis Report

**Data Filename**	A4.d	**Sample Name**	A4
**Sample Type**	Sample	**Position**	P1-E6
**Instrument Name**	Instrument 1	**User Name**	
**Acq Method**	20240114-HRMS-Pos-1.5min-5%H2O.m	**Acquired Time**	2024/4/14 11:52:03
**IRM Calibration Status**	Success	**DA Method**	Default.m
**Comment**			

**Sample Group**		**Info.**	
**Stream Name**	LC 1	**Acquisition SW Version**	6200 series TOF/6500 series Q-TOF B.06.01 (B6172 SP1)

User Spectra

Fragmentor Voltage	Collision Energy	Ionization Mode
175	0	ESI

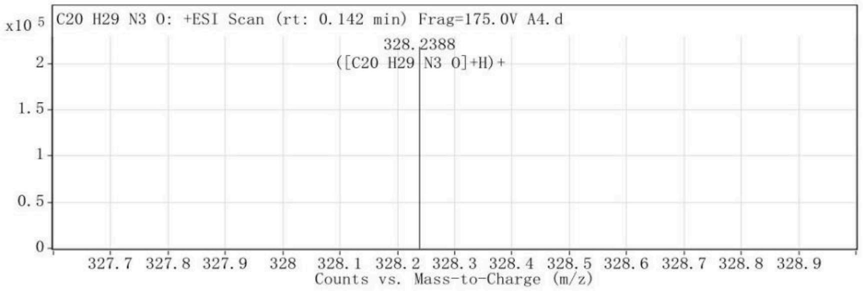

Peak List

m/z	z	Abund
129.1026	1	426390.22
243.1494	1	394304.03
344.2334	1	15878617
344.3884	1	801545.75
345.2363	1	3797059.25
346.24	1	515432.03
366.2158	1	339216.56
382.1897	1	297993.94
709.4416	1	1070635.13
710.4453	1	558454.94

Formula Calculator Element Limits

Element	Min	Max
C	3	60
H	0	120
O	0	30
N	0	30
S	0	5
Cl	0	3

Formula Calculator Results

Formula	Best	Mass	Tgt Mass	Diff (ppm)	Ion Species	Score
C20 H29 N3 O	TRUE	327.2314	327.2311	-0.97	C20 H30 N3 O	95.61

--- End Of Report ---

Figure S66. HRESIMS analysis of **4d**

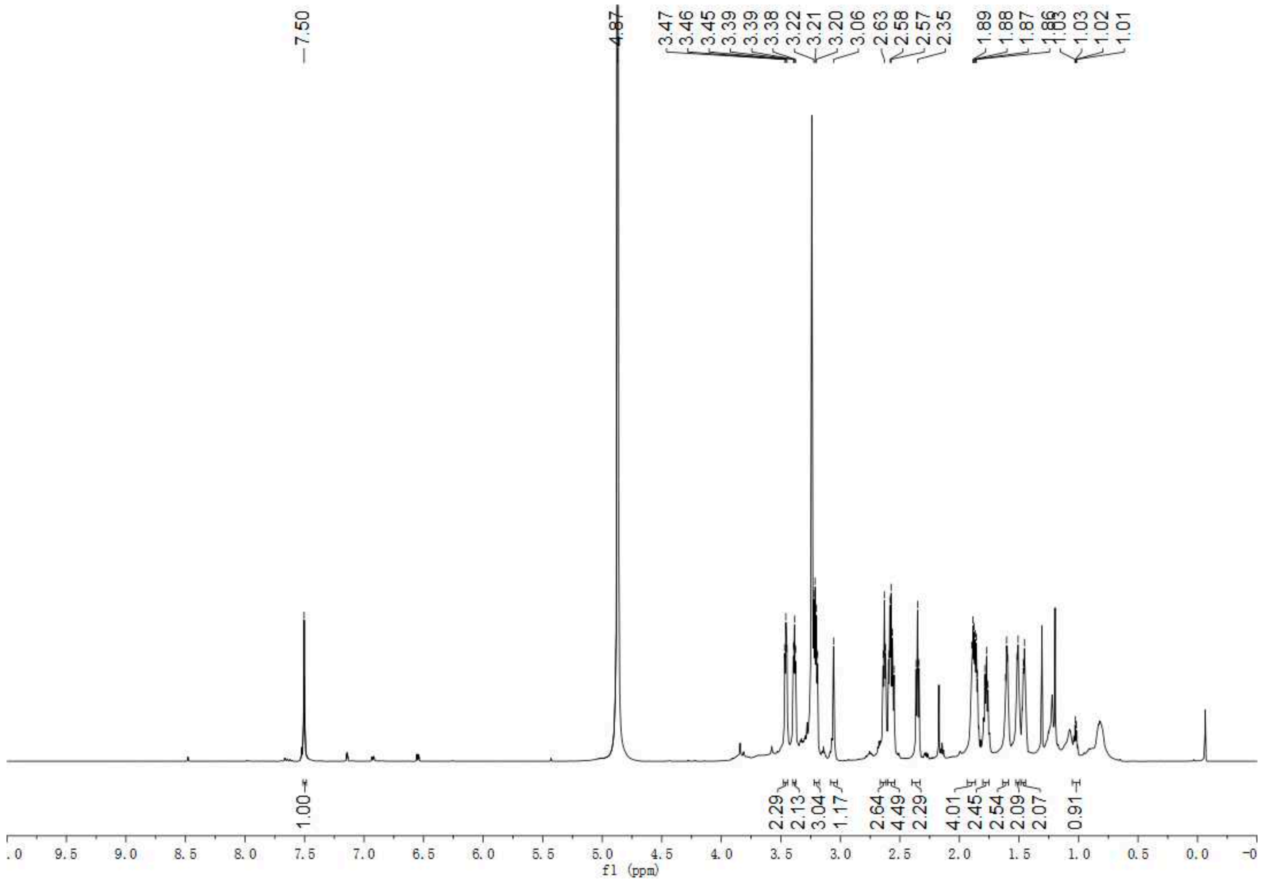

Figure S67. ^{1}H NMR spectrum (600 MHz, CD_3OD) of **4d**

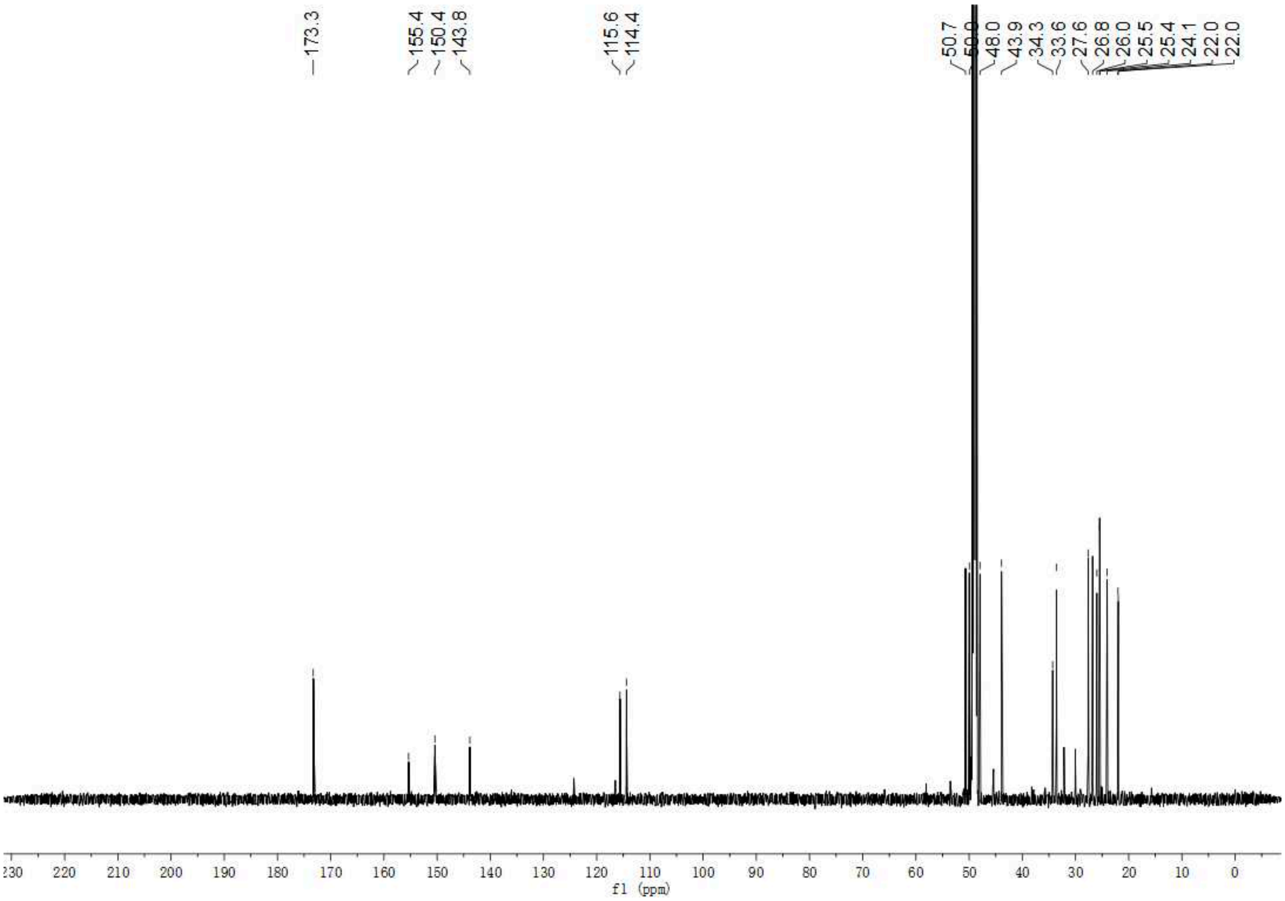

Figure S68. ^{13}C NMR spectrum (150 MHz, CD_3OD) of **4d**

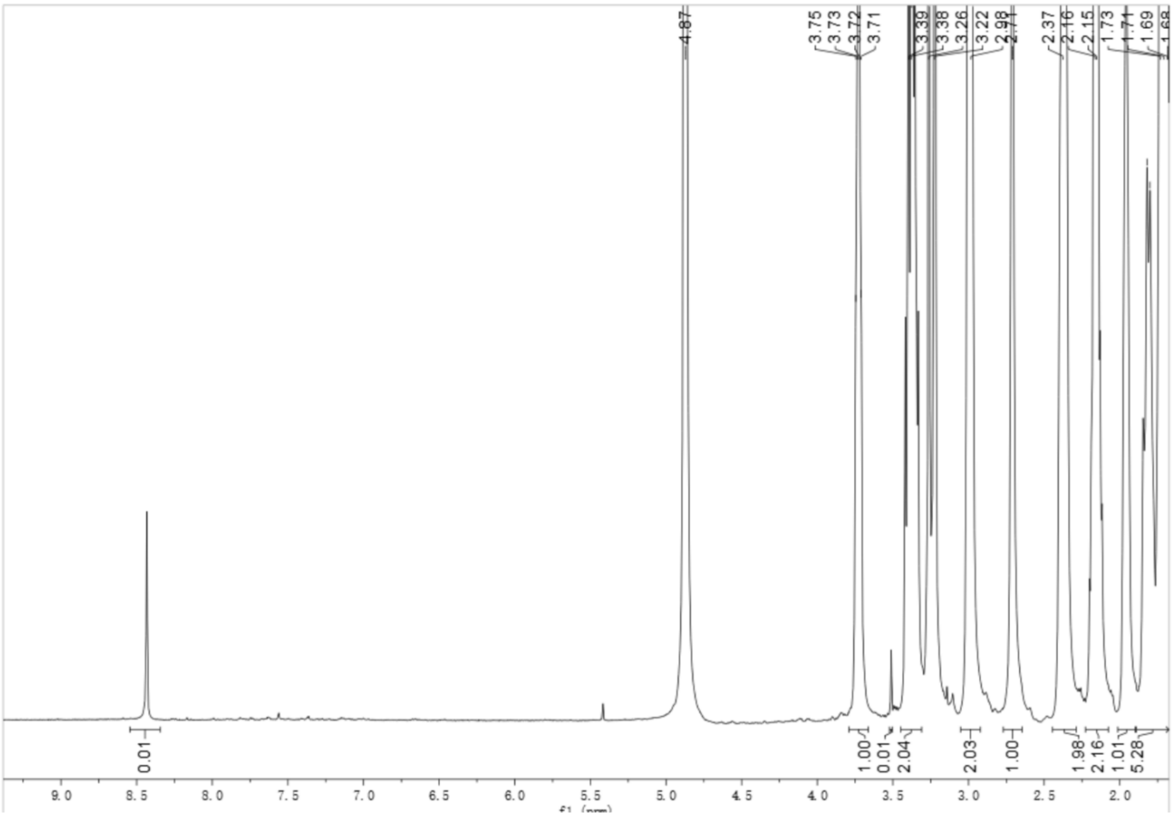

Figure S69. NMR purity of compound **1** (> 99%)

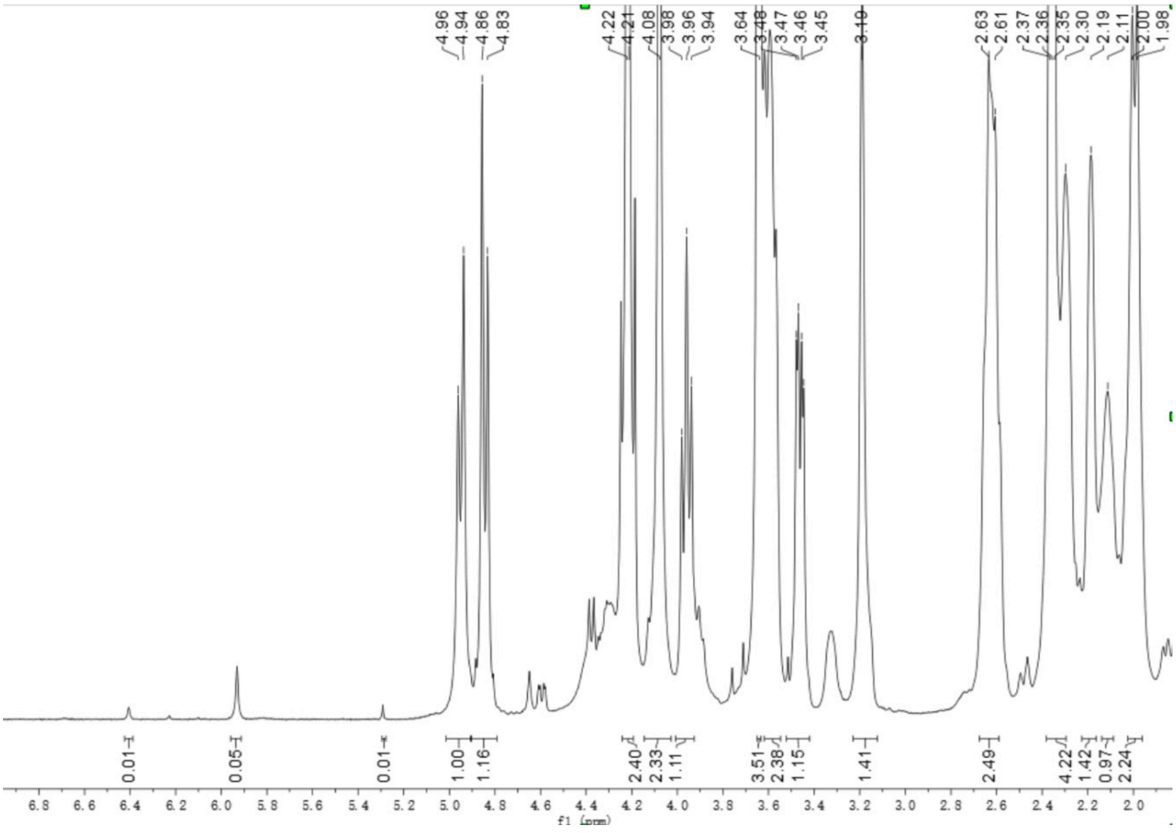

Figure S70. NMR purity of compound **2** (> 95%)

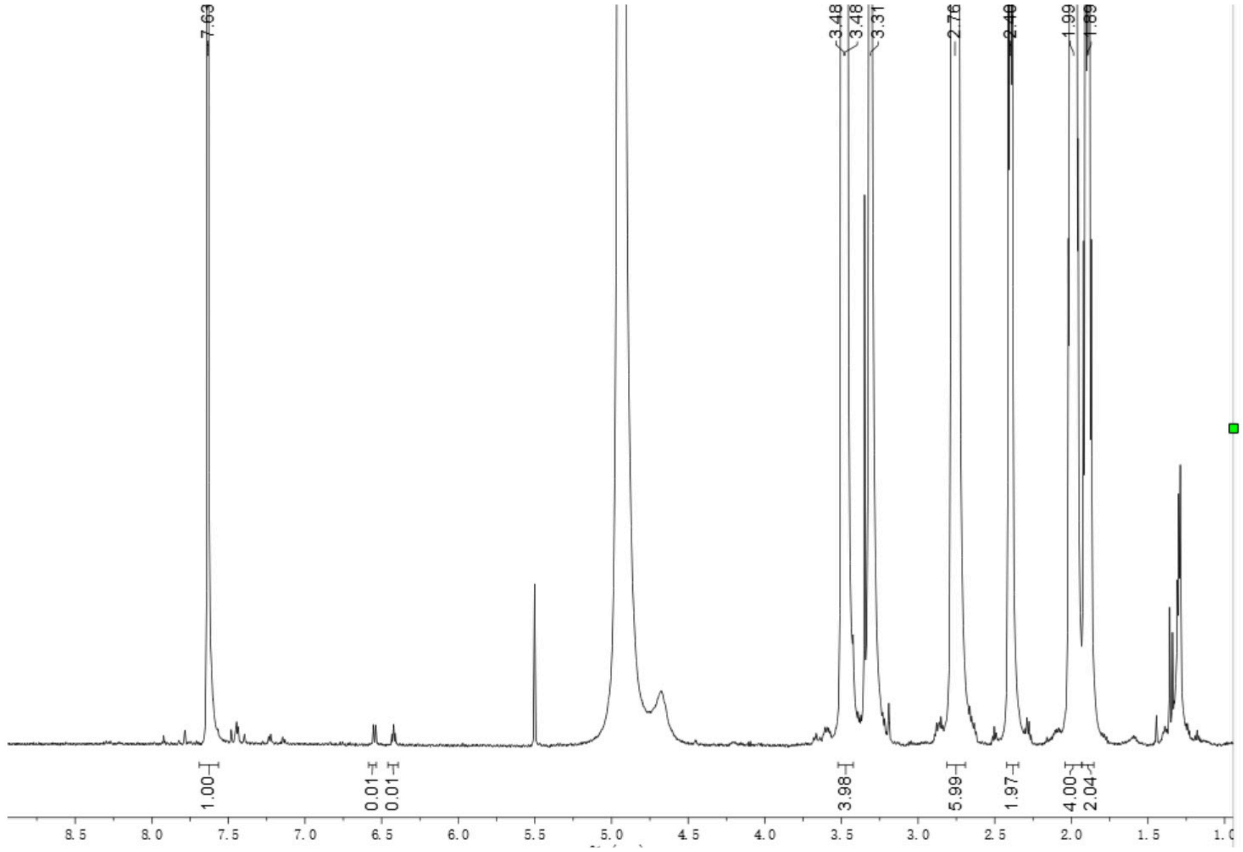

Figure S71. NMR purity of compound **4** (> 99%)

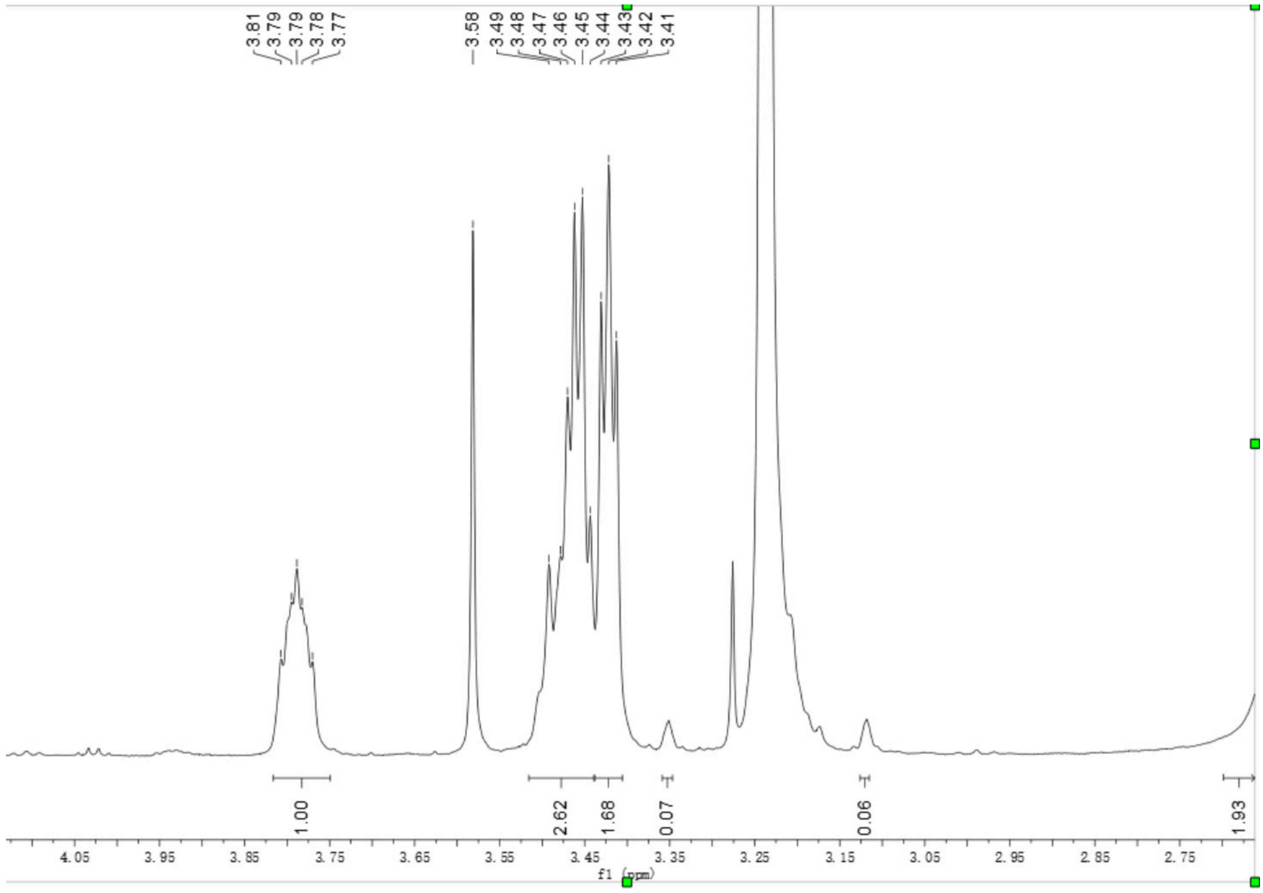

Figure S72. NMR purity of compound **1a** (> 93%)

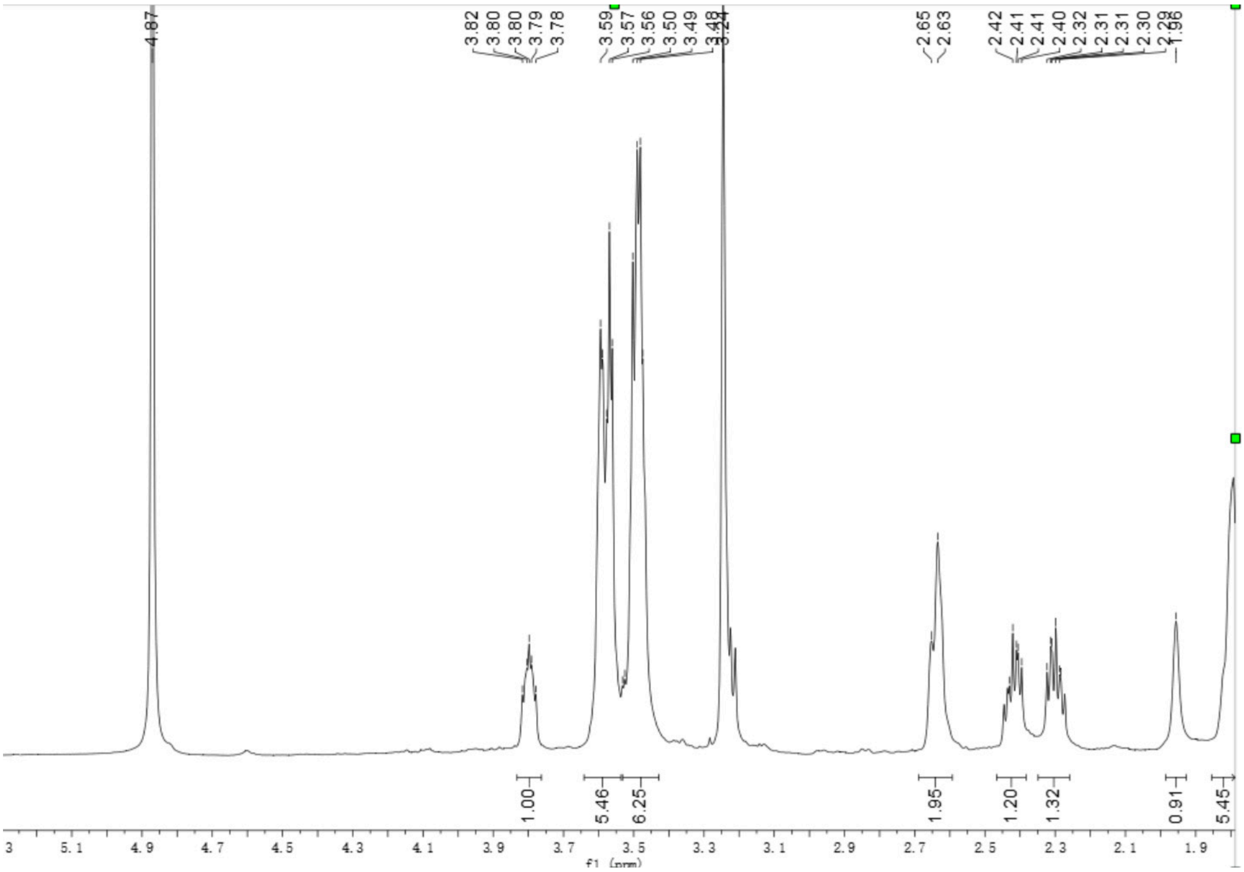

Figure S73. NMR purity of compound **1b** (> 99%)

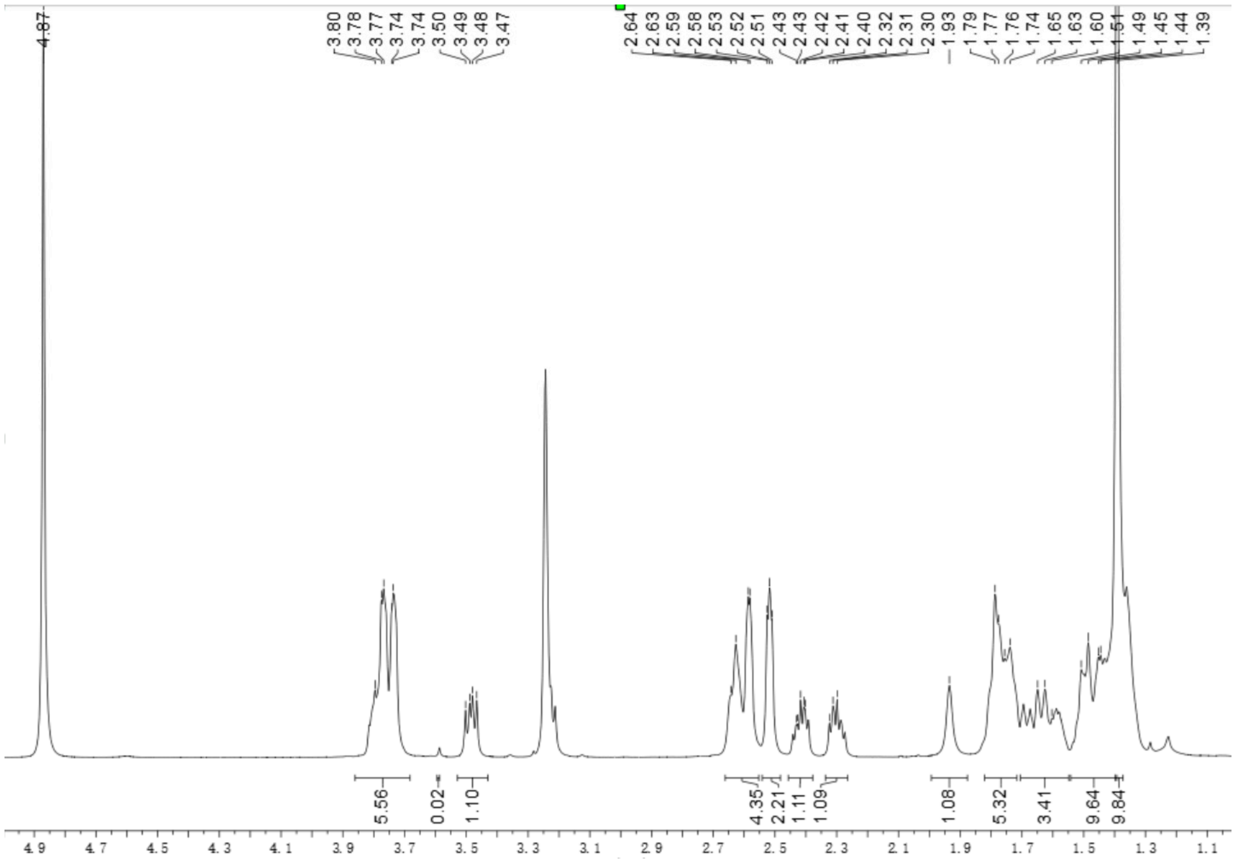

Figure S74. NMR purity of compound **1c** (> 98%)

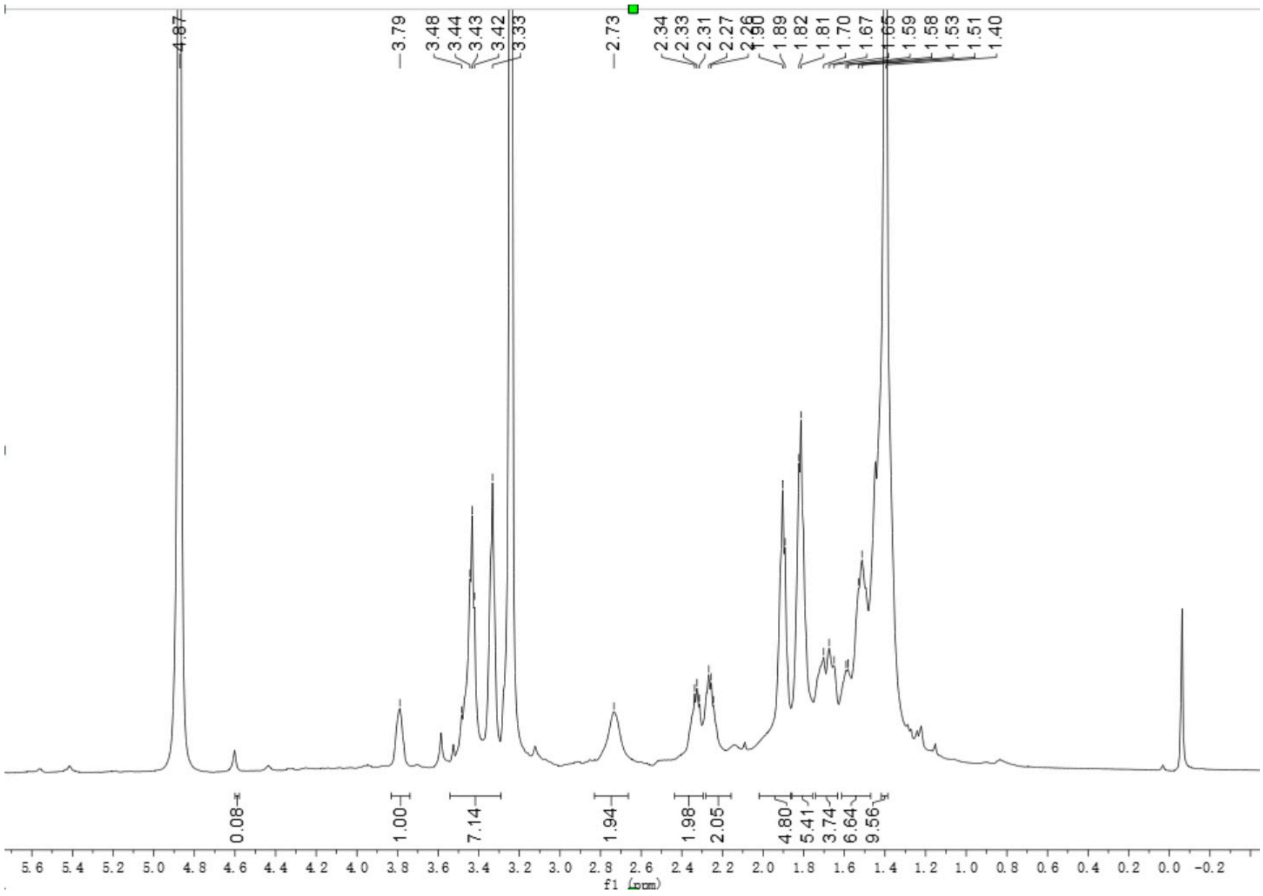

Figure S75. NMR purity of compound **1c** (> 92%)

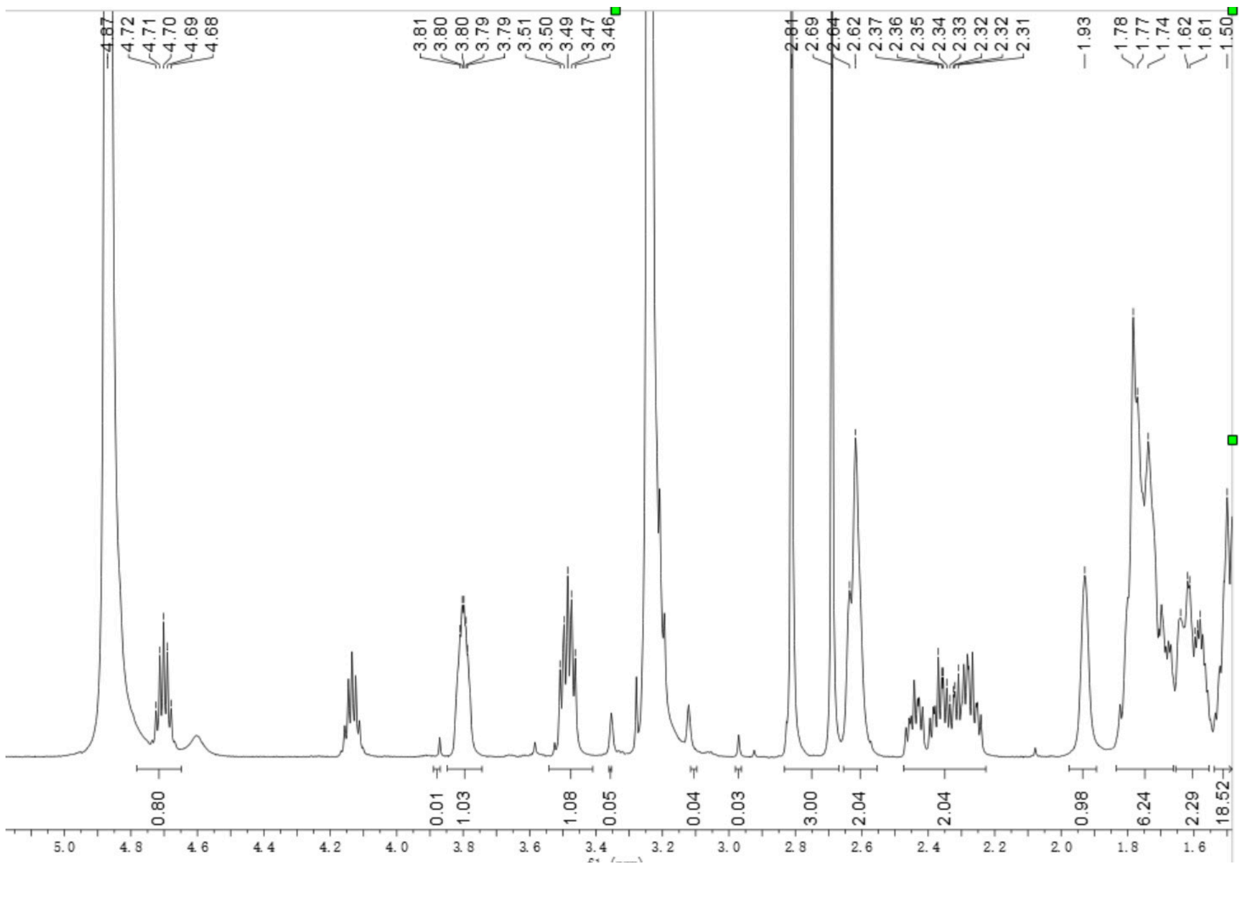

Figure S76. NMR purity of compound **1d** (> 95%)

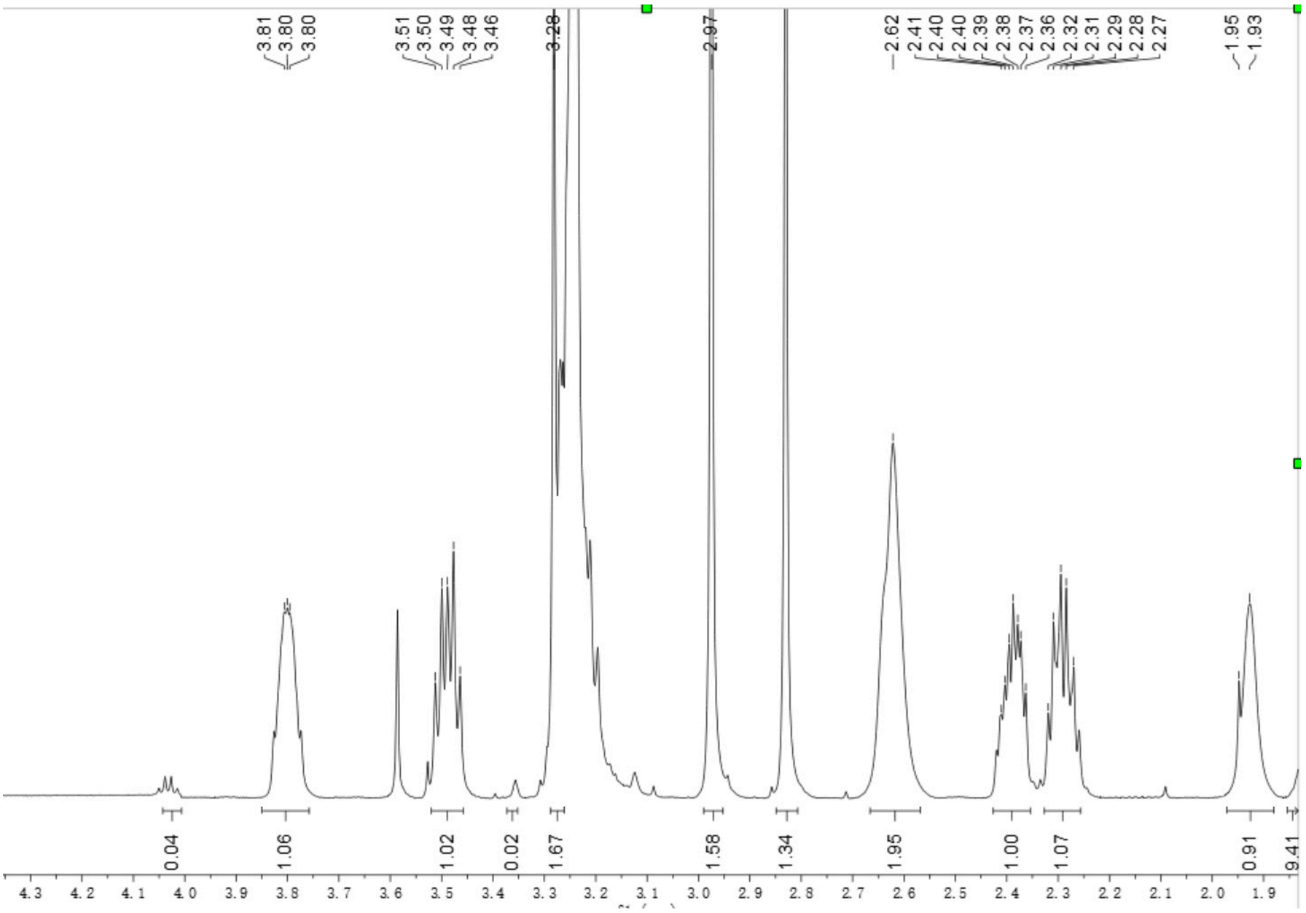

Figure S77. NMR purity of compound **1f** (> 96%)

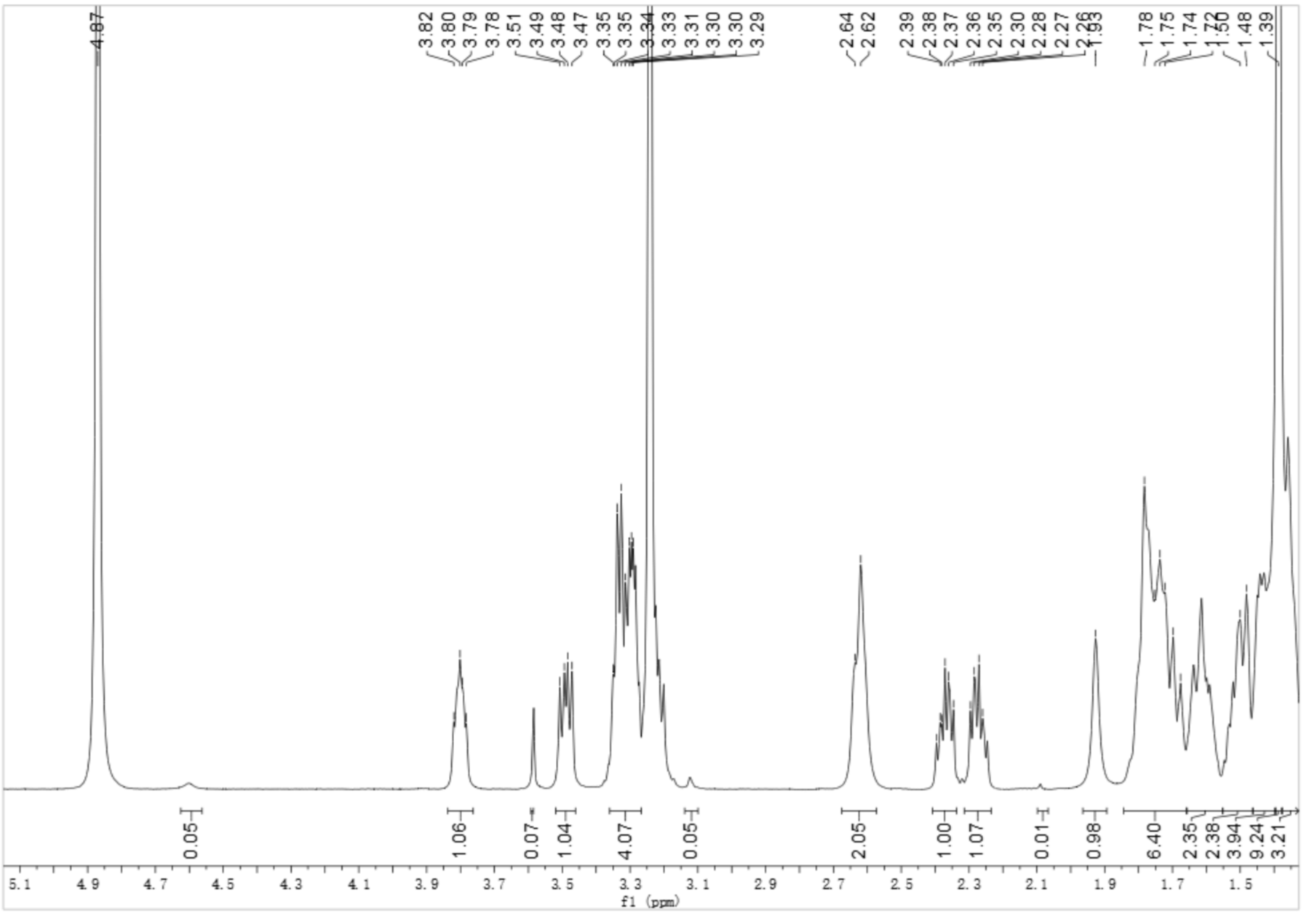

Figure S78. NMR purity of compound **1g** (> 93%)

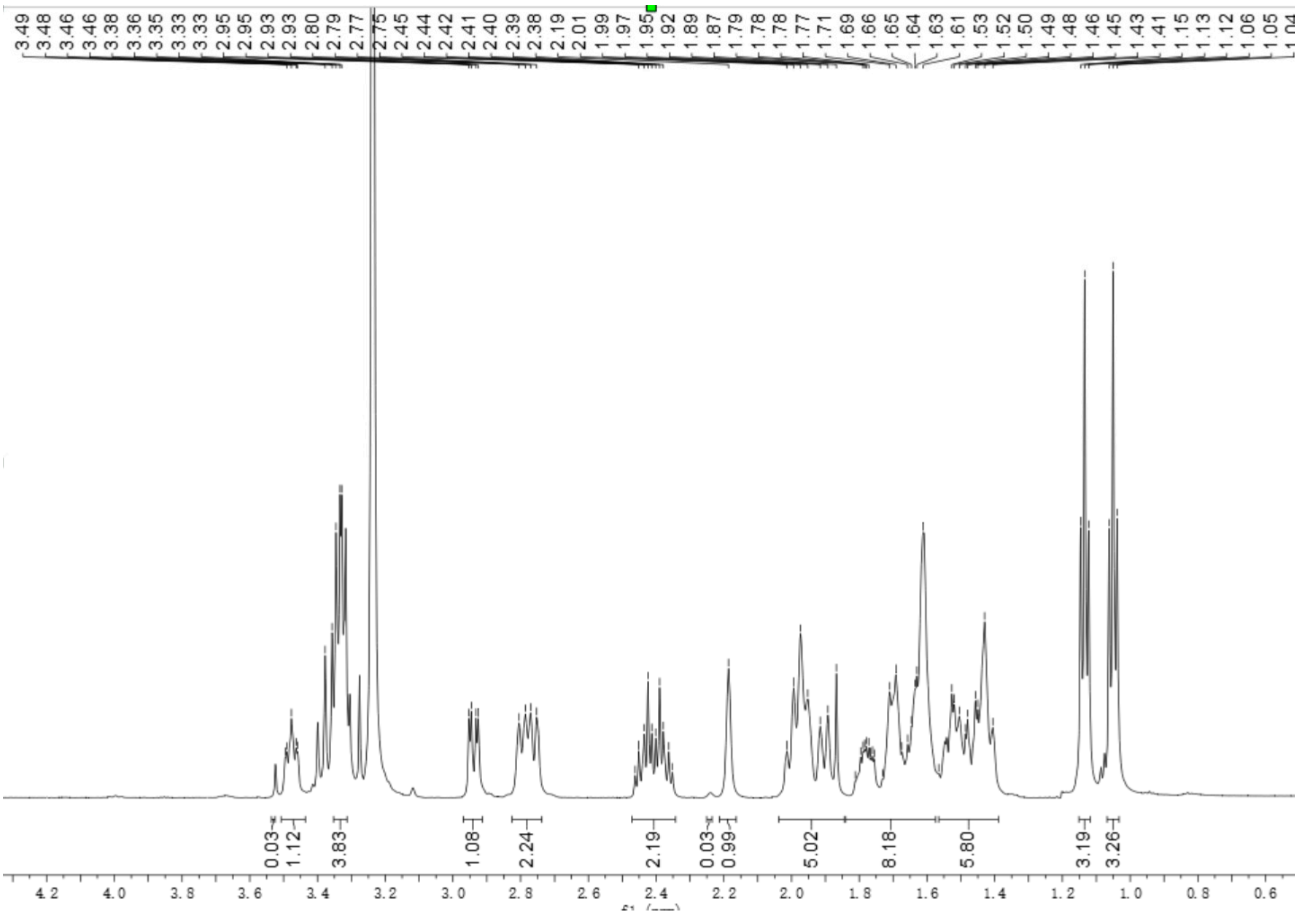

Figure S79. NMR purity of compound **1h** (> 97%)

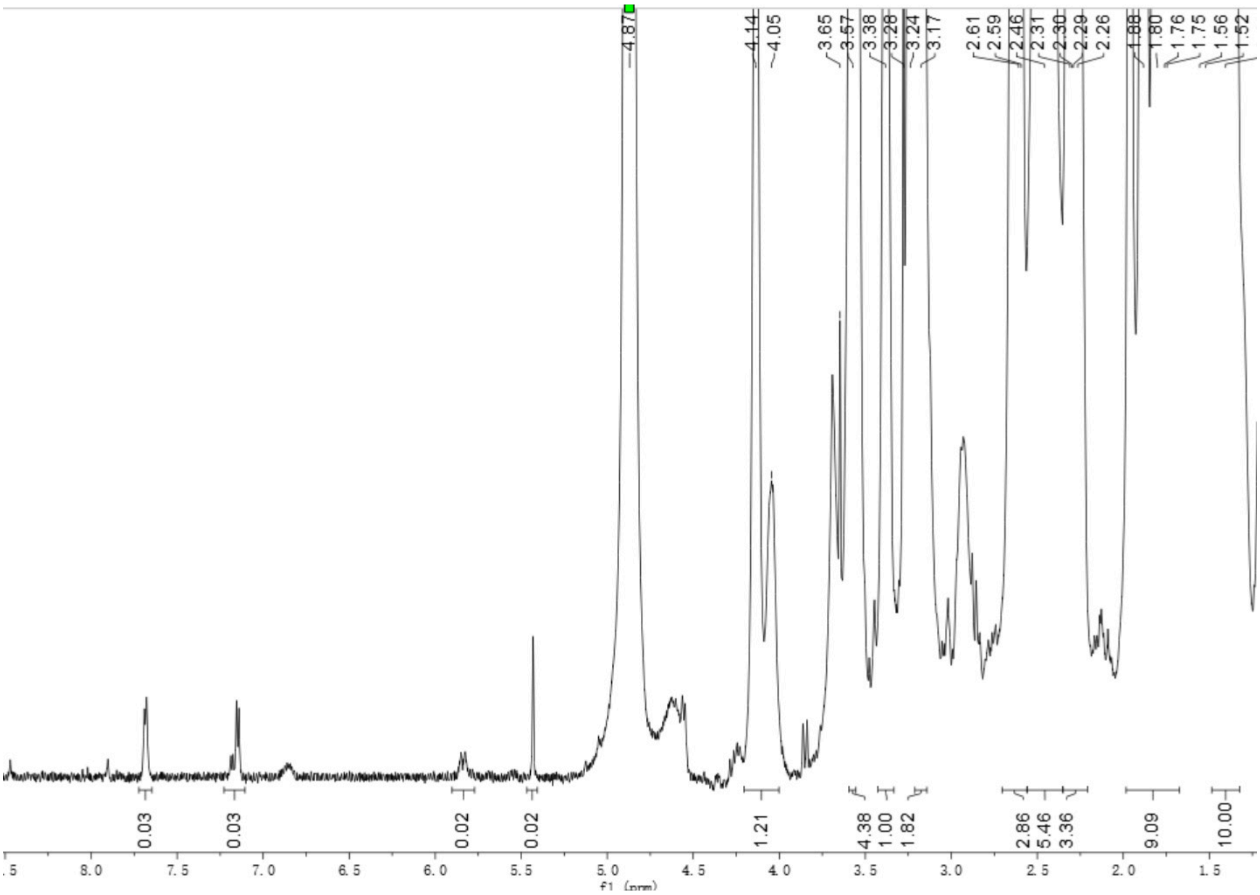

Figure S80. NMR purity of compound **2a** (> 97%)

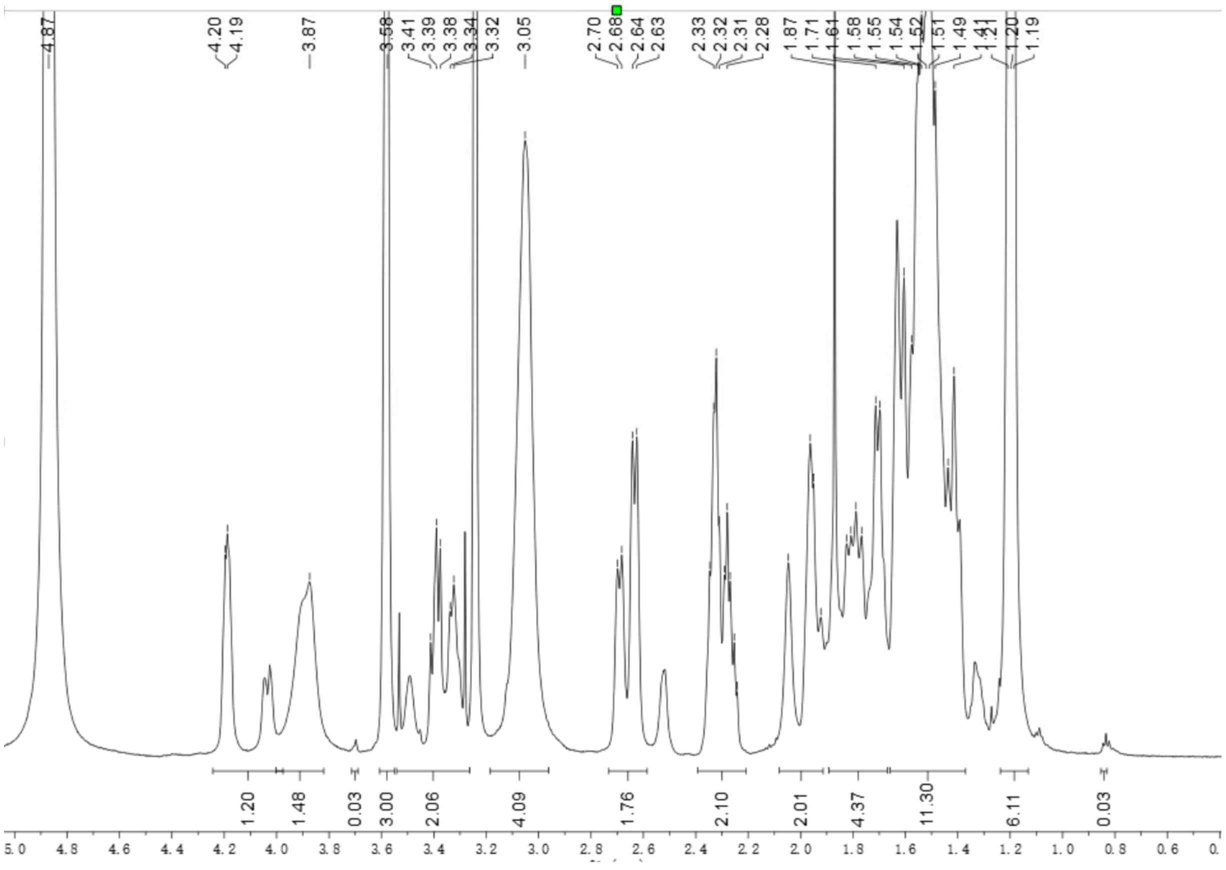

Figure S81. NMR purity of compound **2b** (> 97%)

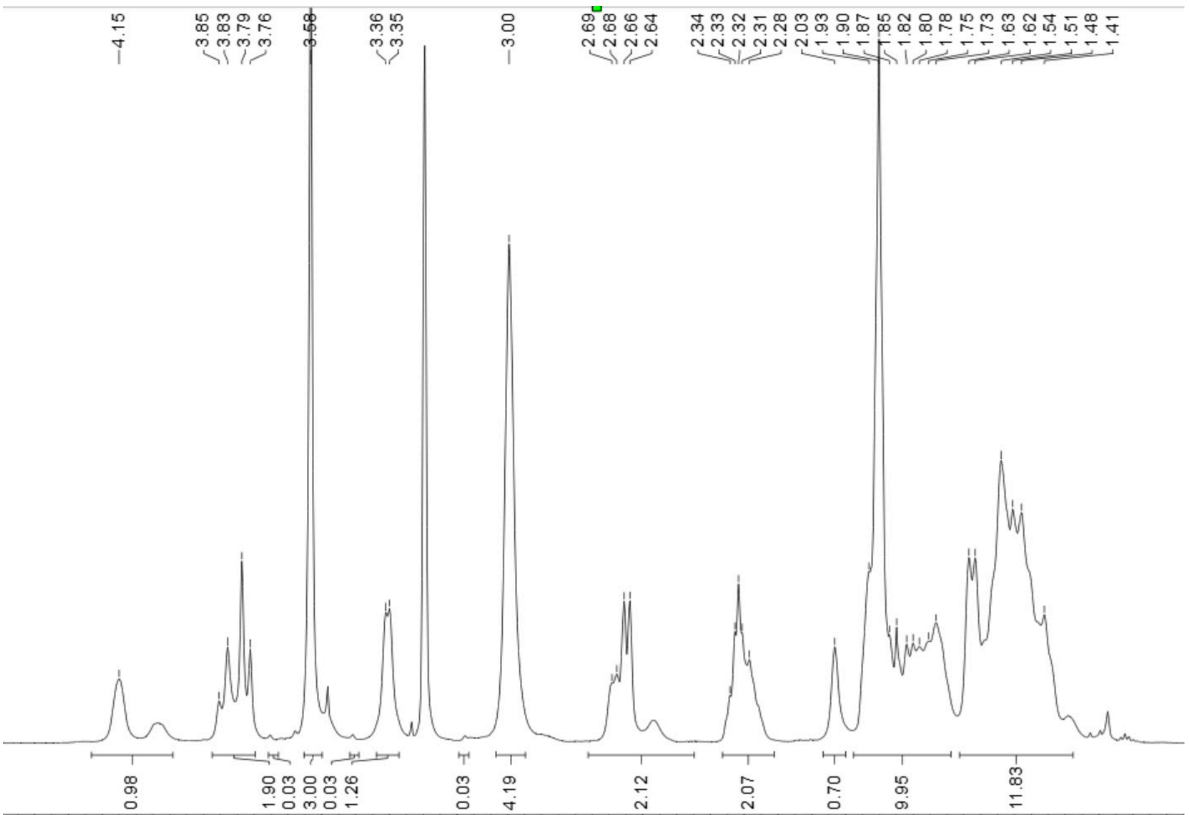

Figure S82. NMR purity of compound **2c** (> 97%)

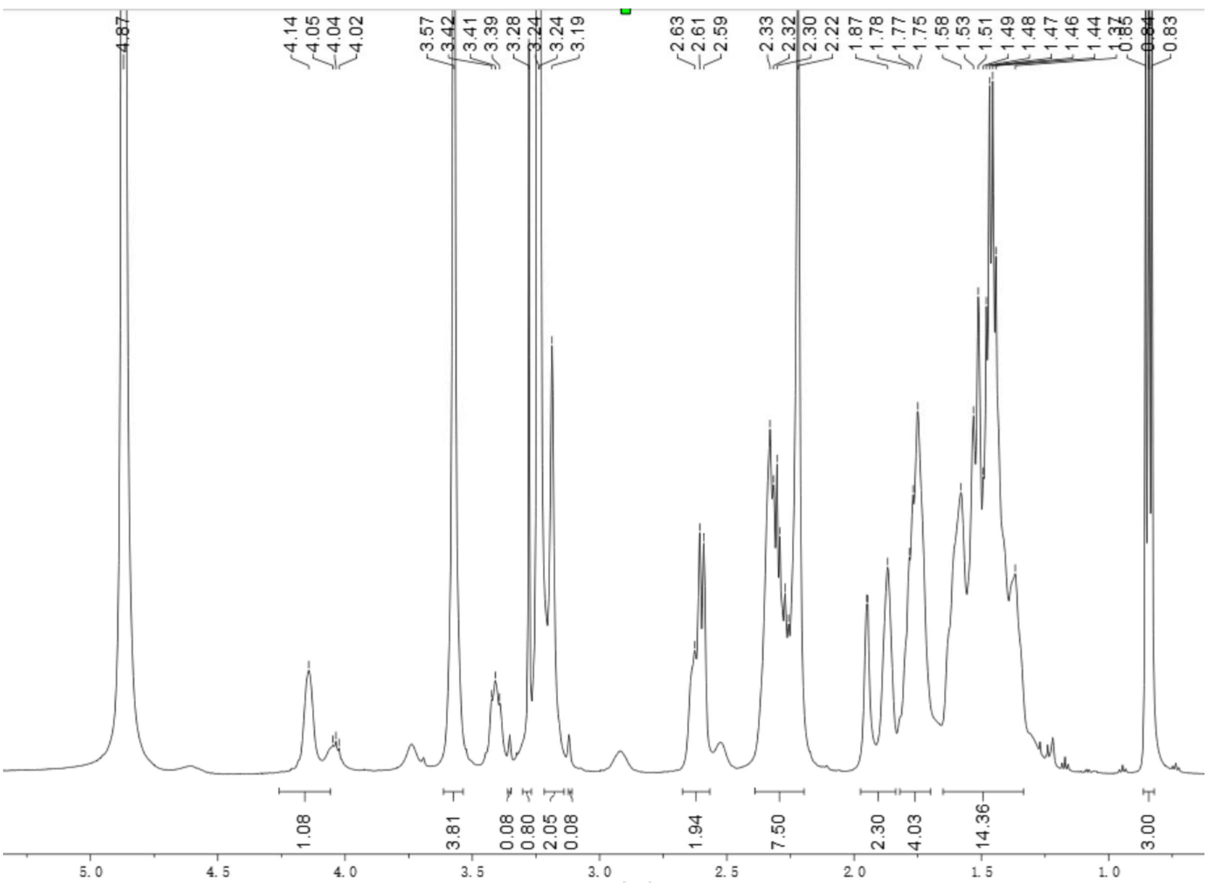

Figure S83. NMR purity of compound **2d** (> 92%)

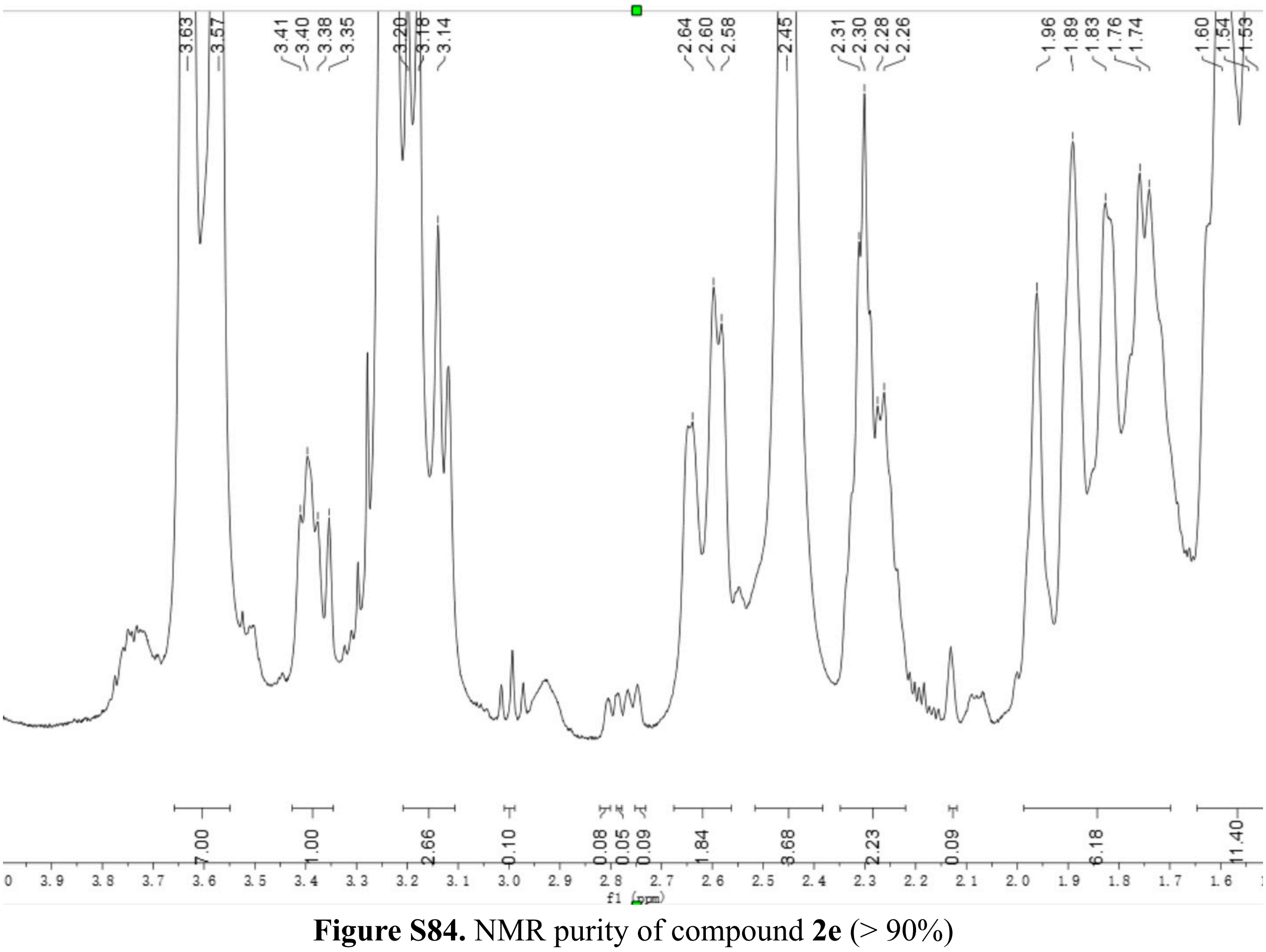

Figure S84. NMR purity of compound **2e** (> 90%)

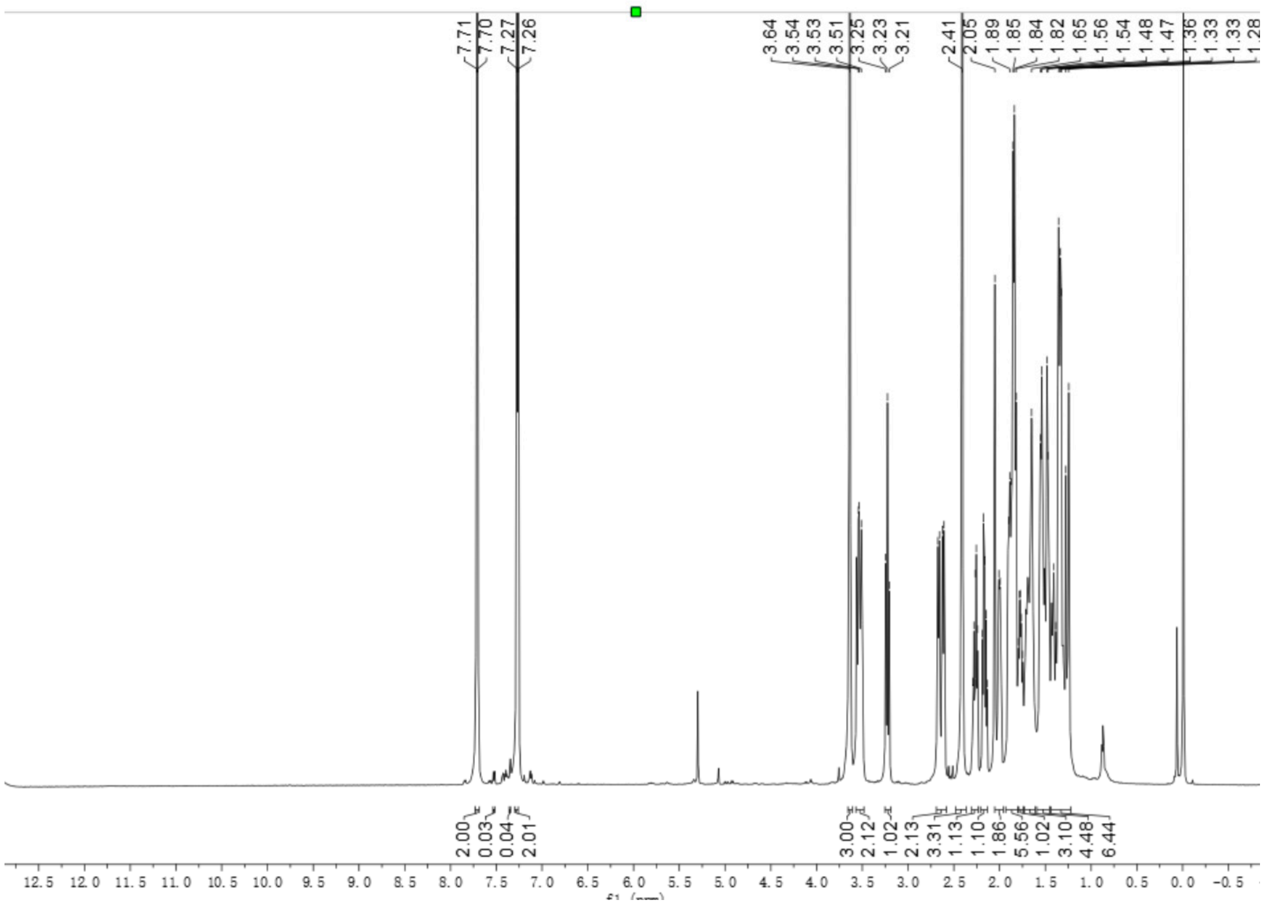

Figure S85. NMR purity of compound **3a** (> 97%)

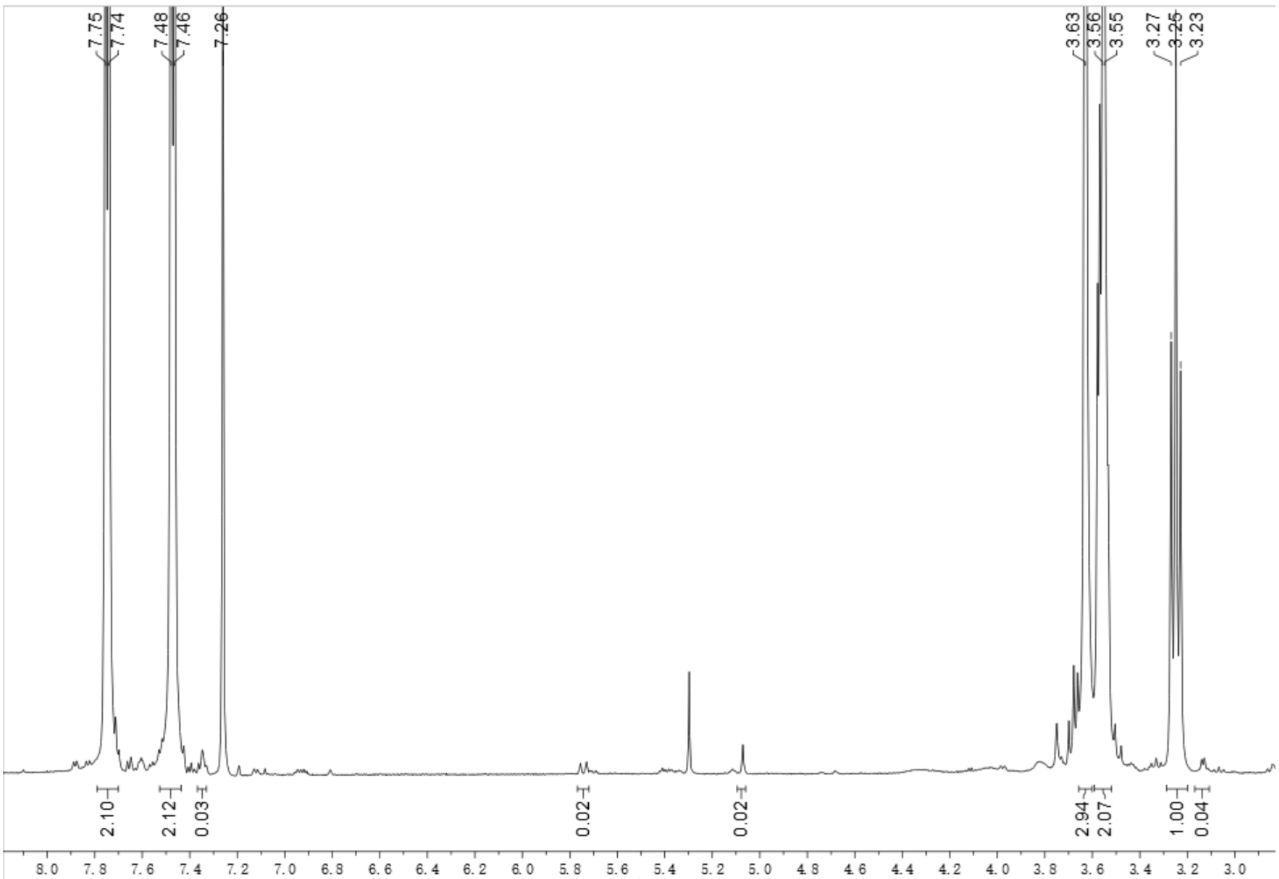

Figure S86. NMR purity of compound **3b** (> 97%)

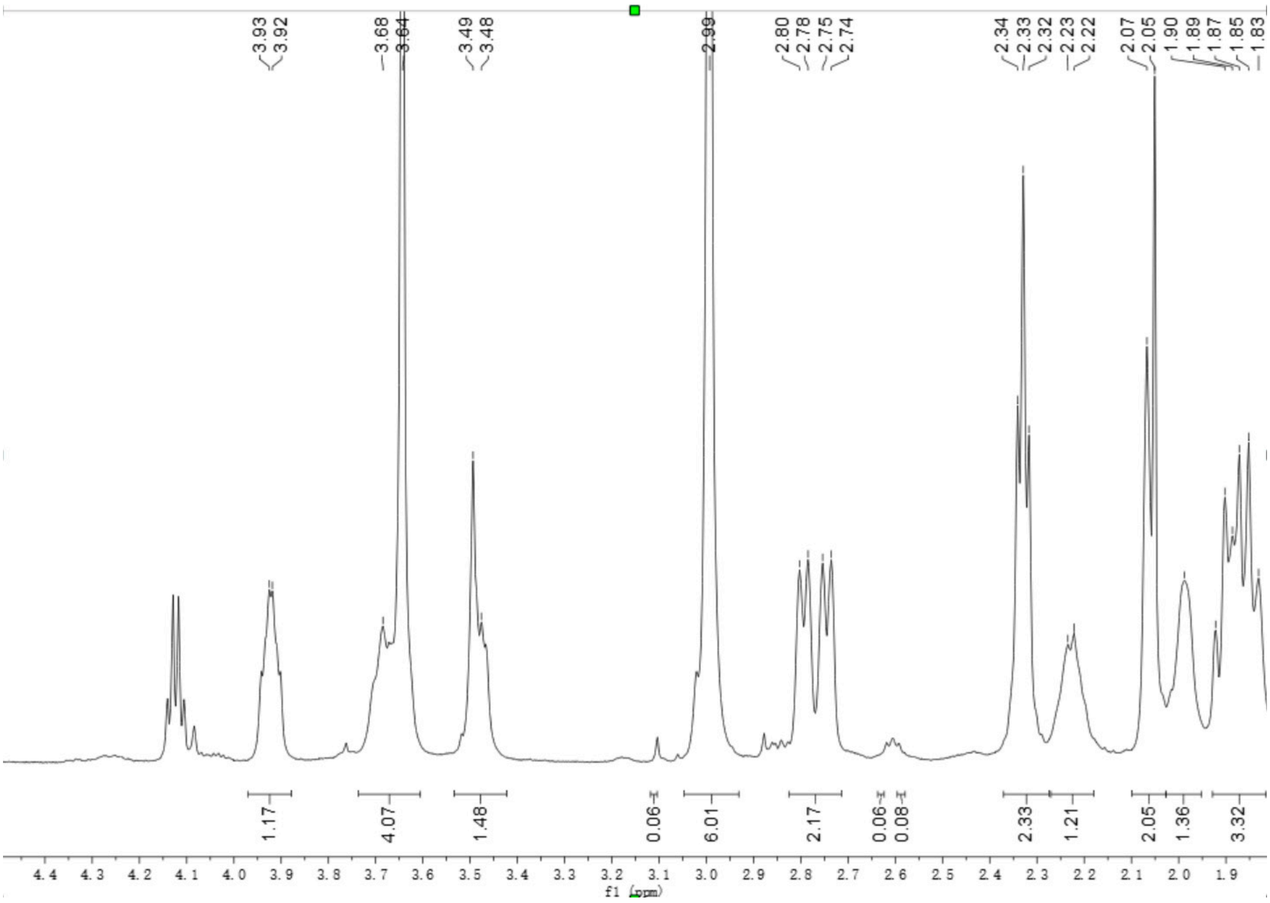

Figure S87. NMR purity of compound **3c** (> 92%)

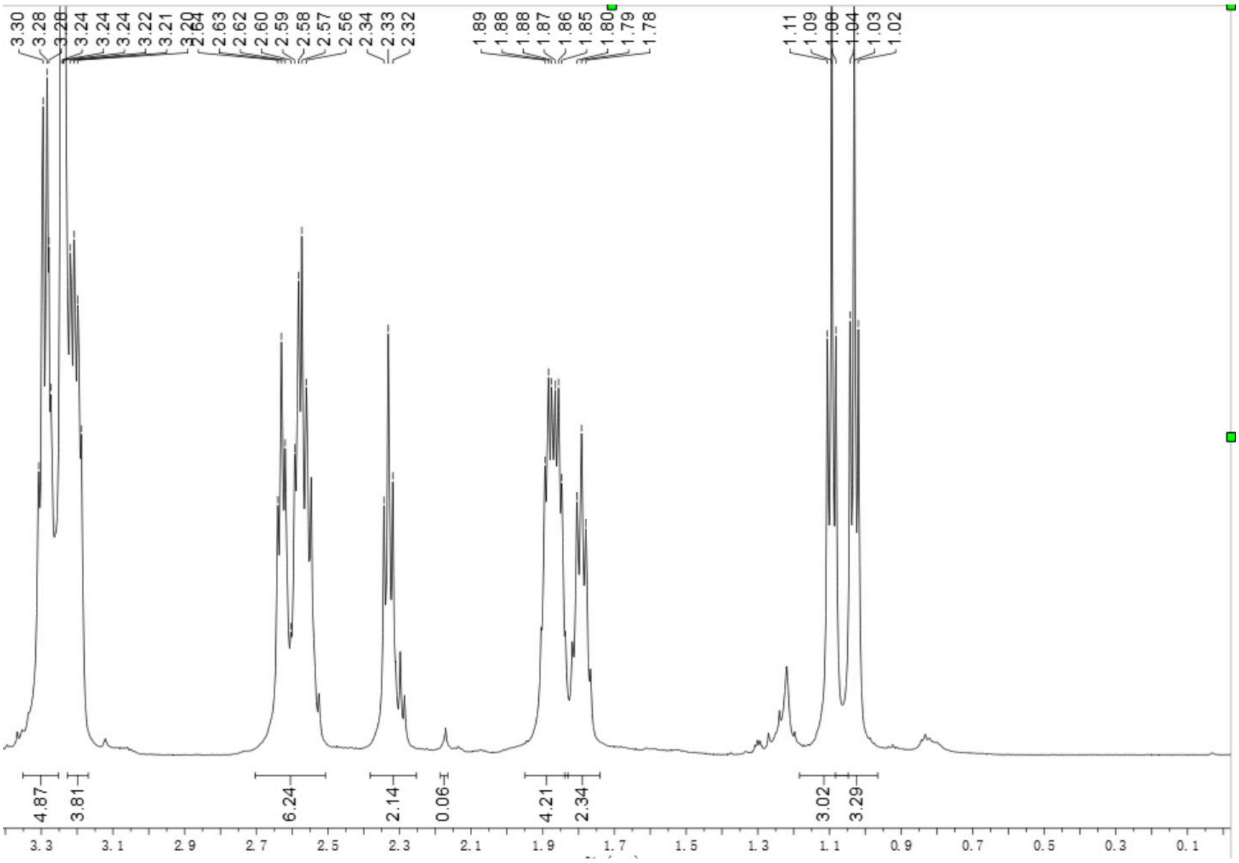

Figure S88. NMR purity of compound **4a** (> 94%)

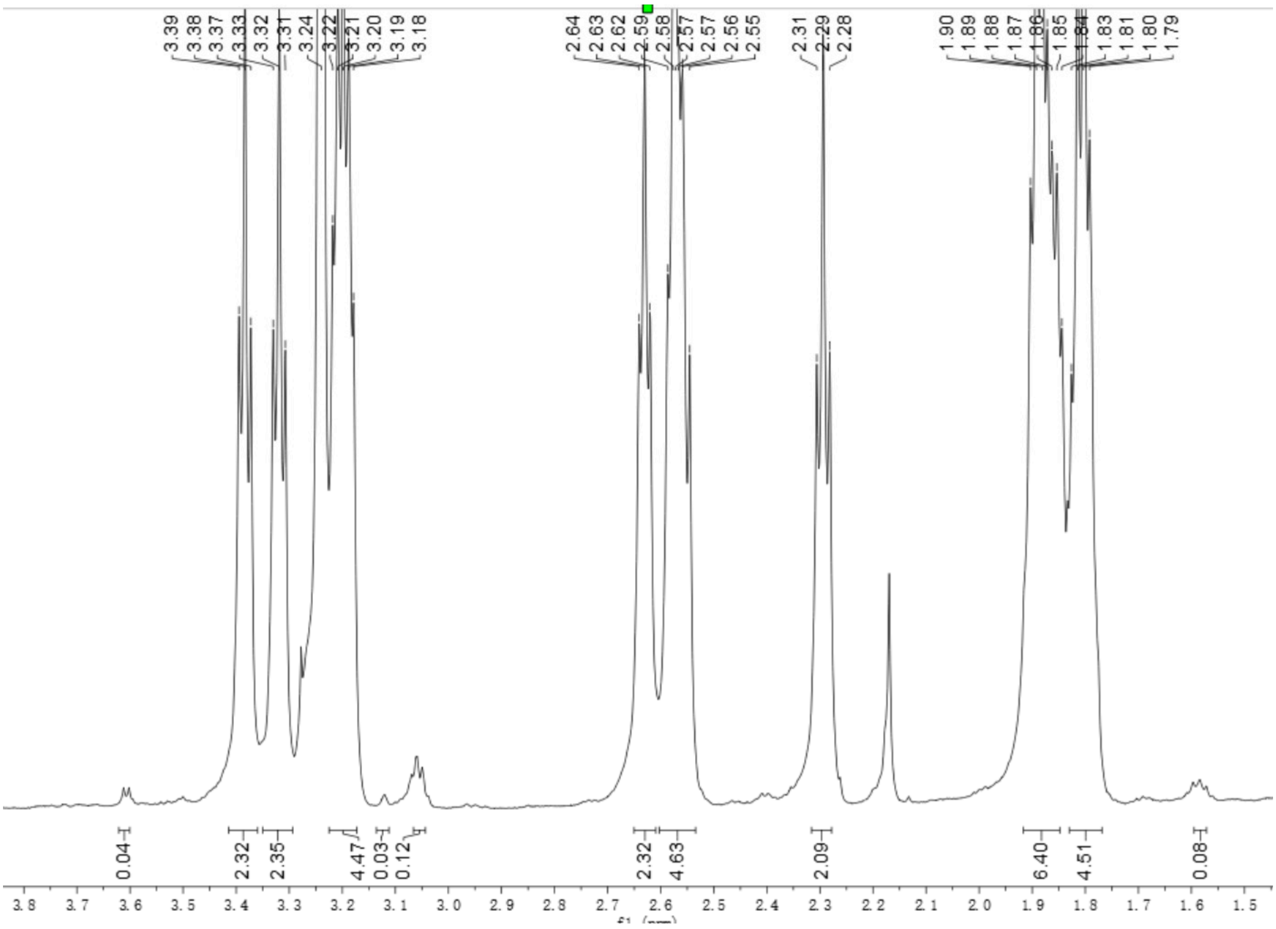

Figure S89. NMR purity of compound **4b** (> 96%)

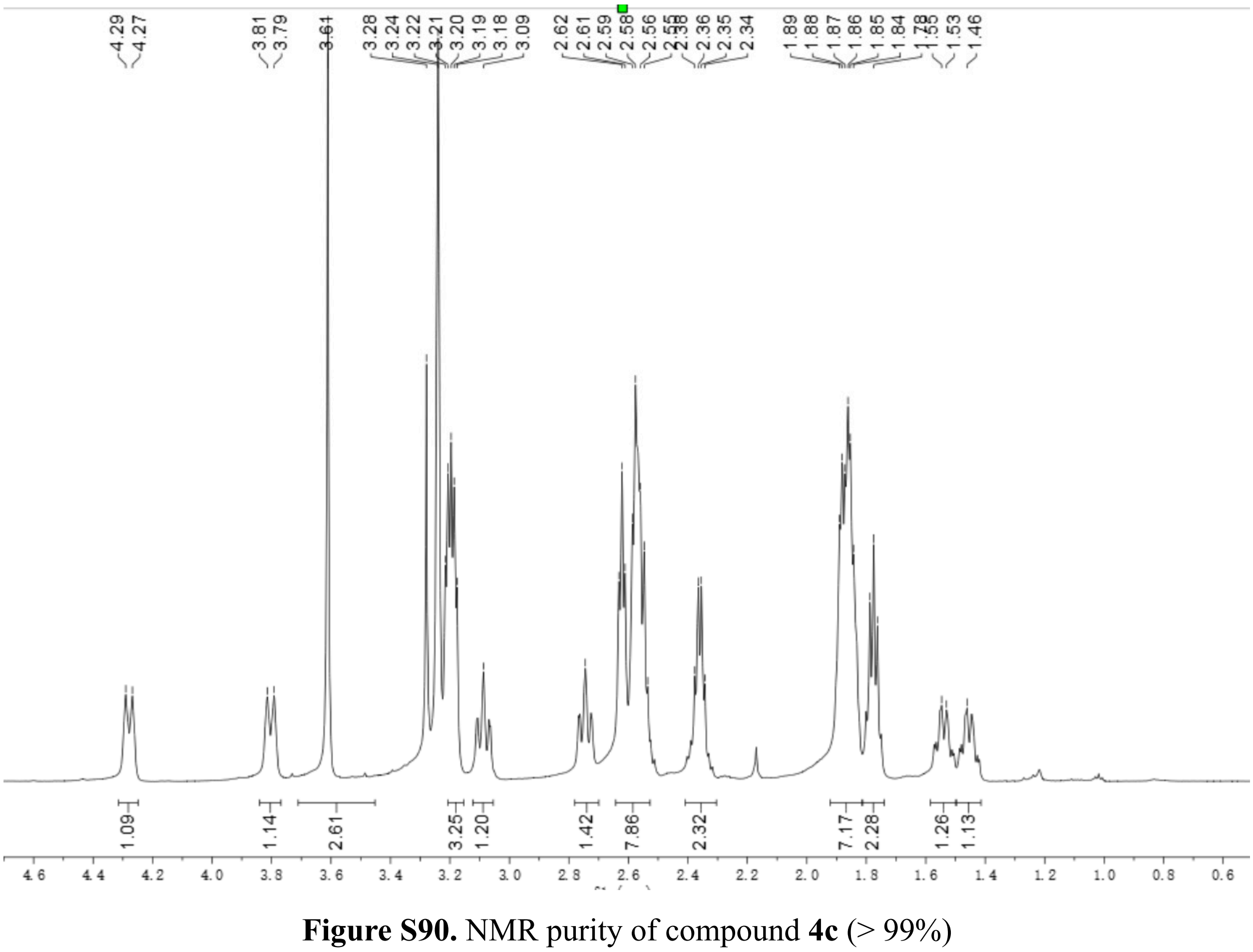

Figure S90. NMR purity of compound **4c** (> 99%)

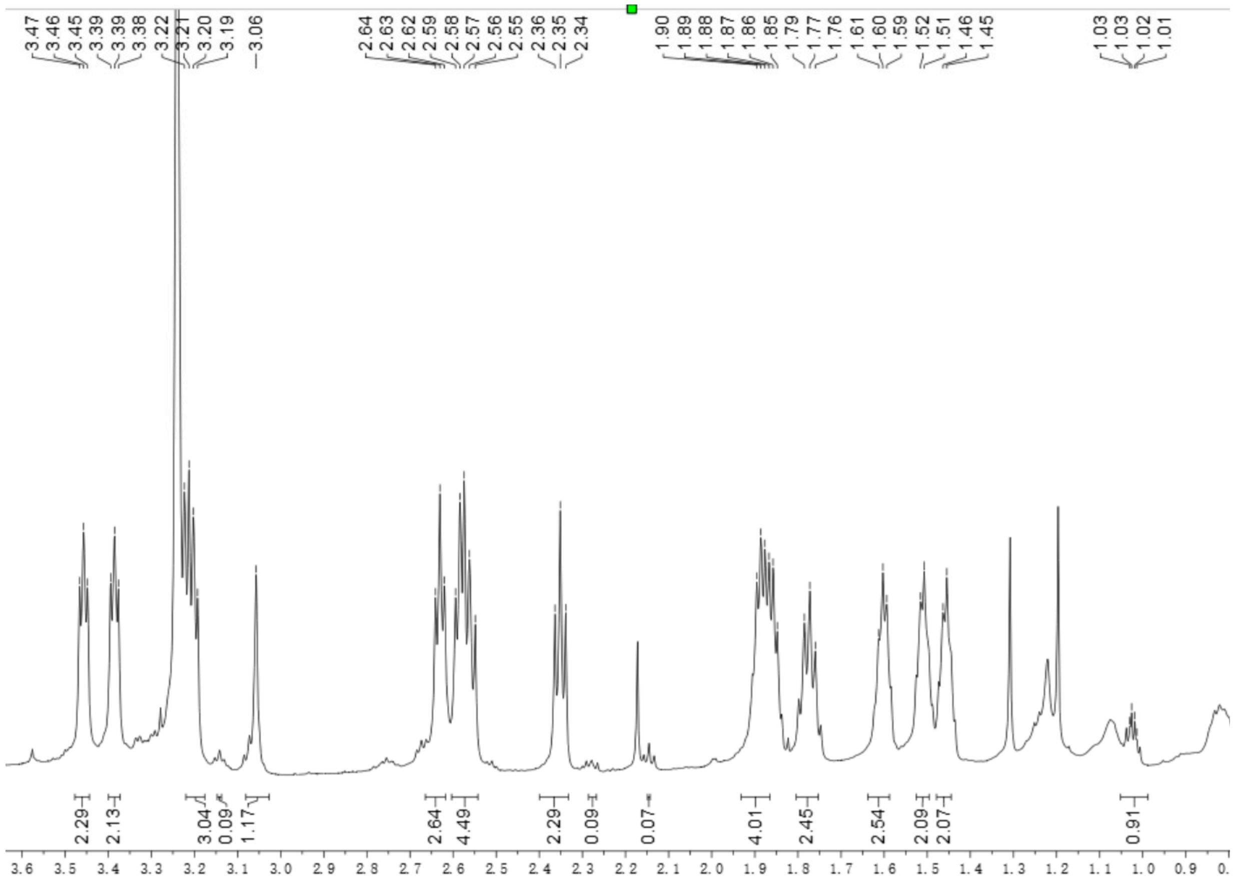

Figure S91. NMR purity of compound **4d** (> 95%)

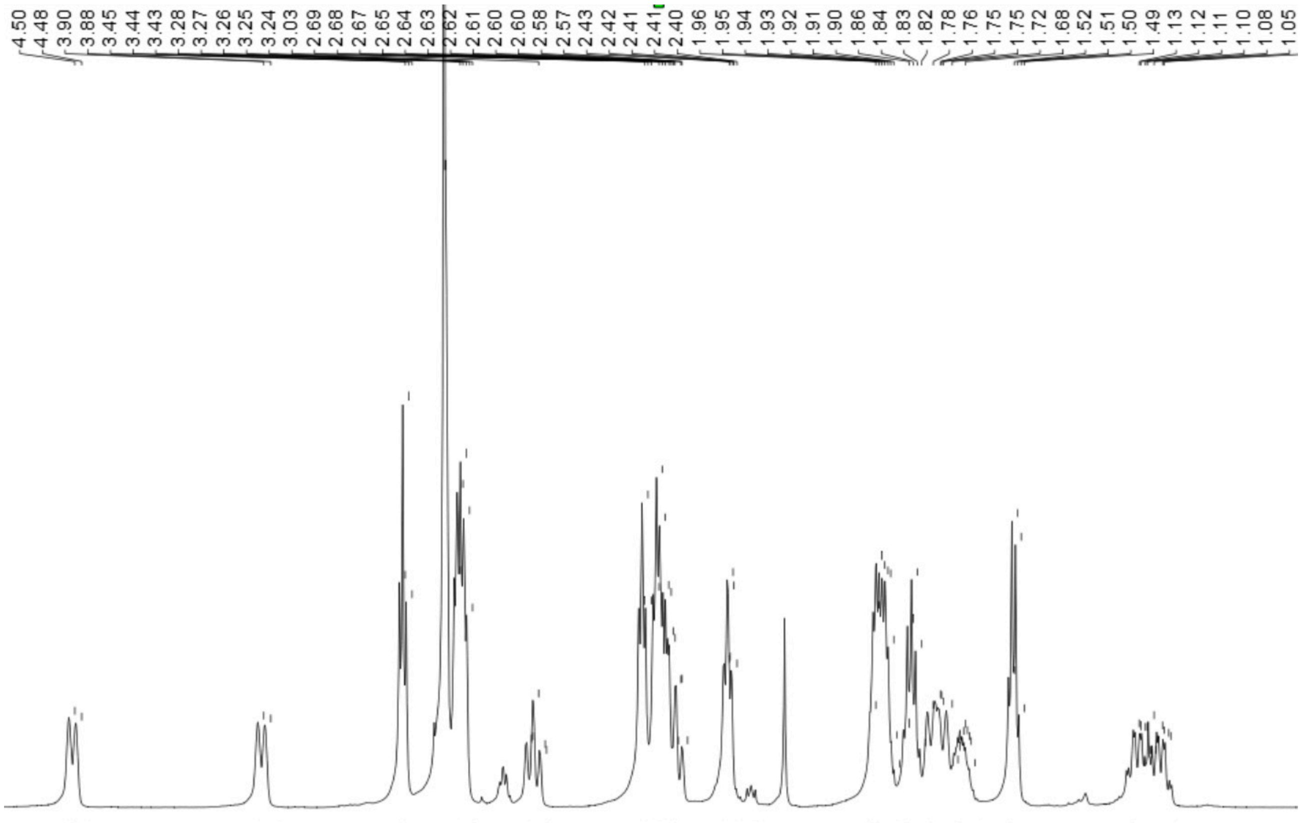

Figure S92. NMR purity of compound **4e** (> 95%)
